# CAMK2D serves as a molecular scaffold for RNF8-MAD2 complex to induce mitotic checkpoint in glioma

**DOI:** 10.1038/s41418-023-01192-3

**Published:** 2023-07-19

**Authors:** You Heng Chuah, Emmy Xue Yun Tay, Oleg V. Grinchuk, Jeehyun Yoon, Jia Feng, Srinivasaraghavan Kannan, Matius Robert, Rekha Jakhar, Yajing Liang, Bernice Woon Li Lee, Loo Chien Wang, Yan Ting Lim, Tianyun Zhao, Radoslaw M. Sobota, Guang Lu, Boon Chuan Low, Karen Carmelina Crasta, Chandra Shekhar Verma, Zhewang Lin, Derrick Sek Tong Ong

**Affiliations:** 1 Department of Physiology, Yong Loo Lin School of Medicine, National University of Singapore, Singapore, 117593 Singapore; 2 NUS Center for Cancer Research, Yong Loo Lin School of Medicine, National University of Singapore, Singapore, Singapore; 3 Bioinformatics Institute, A*STAR (Agency for Science, Technology and Research), Singapore, Singapore; 4 Healthy Longevity Translational Research Programme, Yong Loo Lin School of Medicine, National University of Singapore, Singapore, Singapore; 5 Functional Proteomics Laboratory, SingMass National Laboratory, Institute of Molecular and Cell Biology, Agency for Science, Technology and Research (A*STAR), Singapore, Singapore; 6 Department of Physiology, Zhongshan School of Medicine, Sun Yat-Sen University, Guangzhou, China; 7 Mechanobiology Institute, 5A Engineering Drive 1, National University of Singapore, Singapore, 117411 Singapore; 8 Department of Biological Sciences, 14 Science Drive 4, National University of Singapore, Singapore, 117543 Singapore; 9 University Scholars Programme, 18 College Avenue East, Singapore, 138593 Singapore; 10 Institute of Molecular and Cell Biology (IMCB), Agency for Science, Technology and Research (A*STAR), Singapore, Singapore; 11 School of Biological Sciences, Nanyang Technological University, Singapore, 637551 Singapore; 12 National Neuroscience Institute, Singapore, 308433 Singapore

**Keywords:** Protein-protein interaction networks, Cancer stem cells

## Abstract

MAD2 is a spindle assembly checkpoint protein that participates in the formation of mitotic checkpoint complex, which blocks mitotic progression. RNF8, an established DNA damage response protein, has been implicated in mitotic checkpoint regulation but its exact role remains poorly understood. Here, RNF8 proximity proteomics uncovered a role of RNF8-MAD2 in generating the mitotic checkpoint signal. Specifically, RNF8 competes with a small pool of p31^comet^ for binding to the closed conformer of MAD2 via its RING domain, while CAMK2D serves as a molecular scaffold to concentrate the RNF8-MAD2 complex via transient/weak interactions between its p-Thr287 and RNF8’s FHA domain. Accordingly, RNF8 overexpression impairs glioma stem cell (GSC) mitotic progression in a FHA- and RING-dependent manner. Importantly, low RNF8 expression correlates with inferior glioma outcome and RNF8 overexpression impedes GSC tumorigenicity. Last, we identify PLK1 inhibitor that mimics RNF8 overexpression using a chemical biology approach, and demonstrate a PLK1/HSP90 inhibitor combination that synergistically reduces GSC proliferation and stemness. Thus, our study has unveiled a previously unrecognized CAMK2D-RNF8-MAD2 complex in regulating mitotic checkpoint with relevance to gliomas, which is therapeutically targetable.

## Introduction

Mitotic progression is regulated by kinetochore- (i.e., spindle assembly checkpoint, SAC) and nuclear pore-dependent (that occurs during interphase) checkpoint mechanisms to safeguard chromosome segregation fidelity and genome stability [1, 2]. In particular, MAD2 is crucial for mitotic checkpoint as its MAD1-mediated recruitment to unattached kinetochores and nuclear pores generates the mitotic checkpoint complex (MCC), MAD2-CDC20-BUBR1-BUB3, which serves to inhibit the E3 ubiquitin ligase activity of APC/C^CDC20^, hence preventing the degradation of securin and cyclin B1 that is needed for chromosome segregation and cell cycle progression [2]. At the same time, several competing checkpoint silencing mechanisms exist to restore mitotic progression, including the active disassembly of MCC by TRIP13-p31^comet^, which occurs during mitosis and interphase [3, 4]. Interestingly, there is extensive crosstalk between the DNA damage response (DDR) factors and SAC proteins [5]. For instance, MDC1 is recruited to unattached kinetochores via ATM-mediated H2AX phosphorylation in Nocodazole-treated cells, and this facilitated the localization of MAD2 or CDC20 to kinetochores for SAC activation [6]. Another example is CHK1 which has a mitotic function in enhancing the catalytic activity of Aurora kinase B, and regulating the recruitment of BUBR1 to kinetochores in taxol-mediated mitotic arrested cells [7]. Whether other DDR factors also moonlight for mitotic checkpoint, particularly with relevance to carcinogenesis, remains poorly understood.

RNF8 is an E3 ubiquitin ligase that is recruited to phosphorylated MDC1 in the presence of DNA double-stranded breaks (DSBs), where it works in concert with UBC13 (an E2 conjugating enzyme) to install a K63-linked ubiquitin chain onto histone H1 [8–10]. This modification facilitates the subsequent recruitment of RNF168 to ubiquitinate histone H2A on K15 at DSBs, which is key for the recruitment of 53BP1 and effecting non-homologous end joining [11, 12]. RNF8 is also localized to telomeres where it regulates the stability of Tpp1 (a component of the shelterin complex) and tankyrase 1, and RNF8 loss results in telomere shortening and chromosomal fusions [13, 14]. In addition, RNF8 promotes epithelial-to-mesenchymal transition and chemoresistance in cancer by stabilizing Twist, Akt, and Slug, as well as mammary tumorigenesis by preventing RNF8-mediated Notch1 degradation through RNF8 downregulation [15–18]. It is worth noting that RNF8 invariably modifies its substrates via K63-linked ubiquitination in the aforementioned examples. Curiously, a previous study implicated RNF8 in regulating mitotic exit albeit via poorly understood mechanisms [19].

Glioblastoma (GBM) is the most common and lethal form of diffused astrocytoma in adults. The lack of effective treatment for GBM is attributed to its remarkable intra-tumoral heterogeneity and therapy resistance, which is imparted by GSCs that exhibit stem-cell properties (i.e., can self-renew and differentiate) and extensive plasticity [20]. Thus, patient-derived GSCs represent an invaluable experimental model to study GBM hallmarks, and we have employed the GSCs to discover dependencies of GBM on biotin distribution and H2AZ-mediated chromatin accessibility [21, 22]. Previous studies established that the GSCs are uniquely sensitive to the disruption of chromosome segregation by BUB1B/BUBR1 or BuGZ silencing, as well as inhibition of Aurora kinases and Polo-like kinase 1 (PLK1) [23–26]. Paradoxically, the GSCs exhibit intrinsic chromosomal instability [27] and GBM overexpresses numerous SAC proteins that can interfere with chromosome segregation [1]. A better mechanistic understanding of the mitotic checkpoint regulation in GSC may expand our therapeutic options for GBM patients.

Here, we identified RNF8 as a MAD2 binding protein using BioGRID (https://thebiogrid.org/) and focused on understanding how the RNF8-MAD2 interaction affects mitotic checkpoint response in GSCs. By characterizing the RNF8 proximal proteome, we unraveled a novel mechanism wherein RNF8’s RING domain competes with a small pool of p31^comet^ for c-MAD2 binding, while CAMK2D functions as a molecular scaffold to concentrate the RNF8-MAD2 complex by binding to RNF8’s FHA domain via its p-Thr287, for mitotic checkpoint signal generation. Supporting the physiological relevance of our proposed model, the overexpression of RNF8 compromises GSC proliferation, mitotic progression and genome integrity that is dependent on its FHA and RING domains. In glioma patients, low RNF8 expression is associated with unfavorable patient outcome and RNF8 overexpression suppresses GSC tumorigenicity in an intracranial GBM xenograft model. Using the RNF8 overexpression gene signature for connectivity map analysis (CMA), we further identified PLK1 inhibitors, BI2536 and volasertib, which synergize with 17-AAG in reducing GSC proliferation and stemness, due to the enhanced sensitivity of aneuploid cells to proteotoxic stressors. Our work illuminates a new CAMK2D-RNF8-MAD2-mediated mitotic checkpoint regulatory mechanism with relevance to gliomas, and highlights an indirect approach to target this complex therapeutically in GBM.

## Results

### RNF8 binds to MAD2 to promote MCC formation

**Fig. 1:**
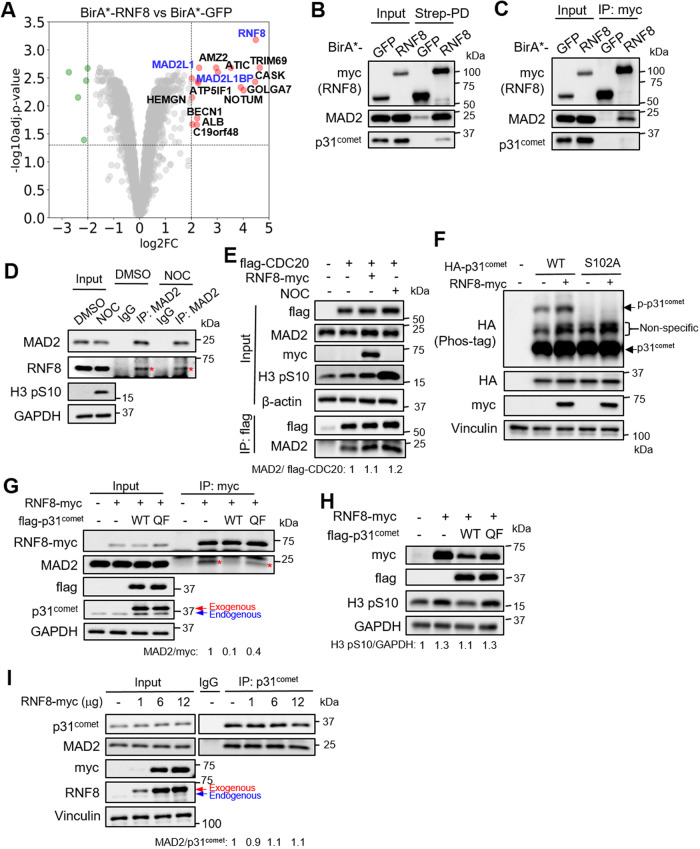
RNF8 binds to MAD2 to promote MCC formation. **A** Volcano plot showing the proteins which were enriched in BirA*-RNF8 compared to BirA*-GFP proximal proteomes. Western blot analysis of MAD2 and p31^comet^ levels in the streptavidin pull down lysates (**B**) or myc immunoprecipitates (IP) (**C**) of HEK293T transfected with BirA*-RNF8 or GFP. **D** Western blot analysis of RNF8 levels in the MAD2 immunoprecipitates of HEK293T, with or without 200 ng/ml NOC (16 h). The band of interest is indicated by *. **E** Western blot analysis of MAD2 and flag-CDC20 levels in the flag IP from HEK293T cells co-transfected with flag-CDC20 and empty vector (EV)/RNF8-myc. Cells with nocodazole (NOC) treatment (200 ng/mL, 16 h) serve as the positive control. The MAD2/flag-CDC20 ratio was normalized to EV control. **F** Phos-tag SDS-PAGE analysis of wild-type or S102A HA-p31^comet^ phosphorylation, with or without RNF8-myc overexpression. **G** Western blot analysis of endogenous MAD2 levels in the myc IP from HEK293T cell lysates overexpressing RNF8-myc, along with or without flag-p31^comet^ or p31^comet(QF)^ overexpression. The band of interest is indicated by *. MAD2/myc ratio was normalized to empty vector control. **H** Western blot analysis of H3 pS10 levels in the cell lysates of HEK293T overexpressing flag-tagged p31^comet^, p31^comet(QF)^, or EV, along with myc-tagged RNF8. GAPDH serves as loading control. H3 pS10/GAPDH ratio was normalized to empty vector control. **I** Western blot analysis of endogenous MAD2 levels in the p31^comet^ immunoprecipitates from HEK293T cell lysates transfected with different amount of RNF8-myc overexpressing plasmids. MAD2/p31^comet^ ratio was normalized to empty vector control.

First, we compared the myc-BirA*-RNF8 vs -GFP proximal proteomes using proximity-dependent biotinylation assay (BioID) coupled with mass spectrometry analysis with the aim of validating the RNF8-MAD2 interaction, as well as identifying RNF8 proximity-dependent protein interactions that may aid in its MAD2 regulation. BioID exploits a promiscuous biotin ligase (BirA*) which biotinylates proteins that are directly or indirectly bound to the bait within a 10 Å radius in the presence of biotin, thereby enabling the isolation of weak/transient protein interactors in a relevant biological context [28]. STRING analysis of the RNF8 interactors showed the enrichment of at least three major pathways, including DDR, SAC and RNA splicing (Fig. 1A; Supplementary Fig. S1A; Supplementary Table S1). While we expected to detect MAD2 (gene symbol *MAD2L1*), we were surprised to find p31^comet^ (gene symbol *MAD2L1BP*), an established MAD2 interactor [29]), among the top RNF8 interactors. Both MAD2 and p31^comet^ were detected in the RNF8 proximal proteome, but only MAD2 was detected in the RNF8 immunoprecipitate, indicating that RNF8 likely associates with MAD2 (Fig. 1B, C). Crucially, we validated the endogenous RNF8-MAD2 interaction in 293T cell lysates, with or without nocodazole treatment, suggesting that this event can occur during interphase and in mitosis (Fig. 1D). Given the critical role of MAD2 in mitotic checkpoint, we next asked if RNF8 overexpression may affect MCC formation. RNF8 overexpression increased the formation of CDC20-MAD2 complex (albeit modestly similar to the nocodazole control) and p31^comet^ phosphorylation at S102 (which is linked to suppression of p31^comet^ activity (with TRIP13) that disassembles MCC [30]) in 293T cells; both supportive of increased MCC formation (Fig. 1E, F; Supplementary Fig. S1B, C). Since the MAD2-p31^comet^ complex is present throughout the cell cycle, we hypothesized that RNF8 may compete with p31^comet^ for MAD2 binding. The co-expression of p31^comet^, but not p31^comet(QF)^ that cannot associate with MAD2 [31], completely abolished MAD2 binding to RNF8 suggesting that RNF8 associates with MAD2 that is partially derived from p31^comet^ (Fig. 1G). Accordingly, the co-expression of wildtype p31^comet^, but not p31^comet(QF)^, blocked the increase in the phosphorylation of histone H3 at Ser10 (H3 pS10) (which is tightly correlated with chromosome condensation during mitosis and represents another widely used readout for mitotic checkpoint [29, 32]) that were afforded by RNF8 overexpression alone (Fig. 1H). Unexpectedly, there was no change in MAD2 levels in the p31^comet^ immunoprecipitates with increasing levels of RNF8 overexpression when compared to the empty vector control, suggesting that RNF8 competes with only a small pool of p31^comet^ for MAD2 binding (Fig. 1I). We conclude that RNF8 binds to MAD2 to promote MCC formation.

### RNF8 associates with c-MAD2 via its RING domain without ubiquitinating MAD2

**Fig. 2:**
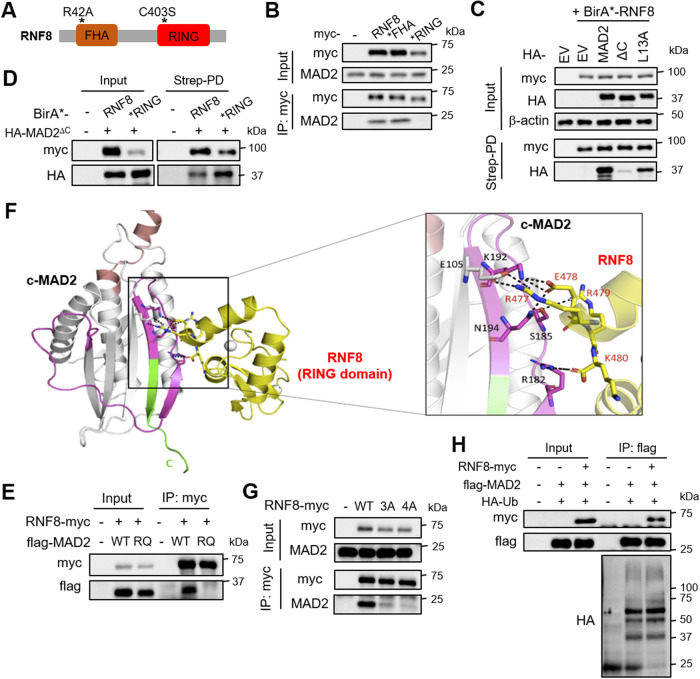
RNF8 associates with c-MAD2 stably via its RING domain without ubiquitinating MAD2. **A** Structure of human RNF8 protein. **B** Western blot analysis of MAD2 levels in the myc IP from HEK293T cell lysates overexpressing myc-tagged RNF8, *FHA, *RING or EV. **C** Western blot analysis of HA-MAD2 levels in the streptavidin pull down lysates of HEK293T transfected with EV, HA-MAD2, -MAD2^Δ^ or –MAD2^L13A^, along with BirA*-RNF8. **D** Western blot analysis of HA-MAD2^∆C^ levels in the streptavidin pull down lysates from HEK293T cell lysates overexpressing BirA*-RNF8 or -*RING, along with HA-MAD2^∆C^ overexpression. **E** Western blot analysis of flag-MAD2 levels in the myc IP from HEK293T cell lysates overexpressing EV, flag-MAD2, or –MAD2^RQ^, along with myc-tagged RNF8. **F** In silico docking of c-MAD2 (gray) and RNF8’s RING domain (yellow) with zinc (gray sphere) using the program HADDOCK. Interacting residues are highlighted in sticks and the interactions are shown as dotted black lines. **G** Western blot analysis of MAD2 levels in the myc IP from HEK293T cell lysates overexpressing myc-tagged RNF8, 3A, or 4A mutants. **H** Western blot analysis of HA levels in flag IP from HEK293T cell lysates overexpressing flag-tagged MAD2 and HA-tagged Ub, with RNF8 overexpression.

The RNF8 protein harbors an N-terminal forkhead-associated (FHA) domain and a C-terminal E3 ubiquitin ligase (RING) domain, and the *FHA (RNF8^R42A^) and *RING (RNF8^C403S^) mutants have been used to study the importance of these domains in DDR [8, 33] (Fig. 2A). To understand how RNF8 may bind to MAD2, we immunoprecipitated myc-tagged RNF8, *FHA, or *RING from 293T lysates, and found that MAD2 binding was specifically abolished with *RING (Fig. 2B). This was confirmed in a reverse co-IP experiment using flag-tagged MAD2 immunoprecipitates (Supplementary Fig. S2A). Indeed, BioID assay using the *RING mutant failed to detect MAD2 and p31^comet^, concordant with our view that RNF8 competes with p31^comet^ for MAD2 binding (Supplementary Fig. S2B). Using established MAD2 mutants, we next addressed the structural basis for MAD2 binding to RNF8. Only HA-tagged MAD2^L13A^ (closed MAD2 conformer, c-MAD2), but not MAD2^ΔC^ (open MAD2 conformer, o-MAD2) [34], was readily detected in the RNF8 proximal proteome, indicating that RNF8 binds more strongly to c-MAD2 compared to o-MAD2 (Fig. 2C). Interestingly, we found more MAD2^ΔC^ in the *RING vs RNF8 proximal proteomes, suggesting that the RNF8’s RING domain samples both c-MAD2 and o-MAD2, but preferentially binds to c-MAD2 (Fig. 2D). Furthermore, RNF8 associates with MAD2 but not MAD2^RQ^ that does not dimerize with itself (i.e., c-MAD2-o-MAD2) or p31^comet^ [35], suggesting that MAD2 can only bind to RNF8 as a homo- or hetero-dimer (Fig. 2E). Molecular modeling using the program HADDOCK (High Ambiguity Driven biomolecular Docking) [36] followed by refinement using Molecular Dynamics simulations showed that the interactions between c-MAD2 and RNF8’s RING domain were primarily mediated by electrostatics, with charged and polar residues (E105, K192, R182, N194, and S185) from c-MAD2 interacting with the complementary charged residues (R477, E478, R479, and K480) from RNF8’s RING domain (Fig. 2F). This predicted mode of interaction was subsequently validated by mutagenesis, whereby a three-point alanine mutation at R477, E478 and R479 in RNF8 (i.e., RNF8^3A^) was sufficient to disrupt the RNF8-MAD2 interaction (Fig. 2G). Notably, neither RNF8 overexpression nor depletion altered MAD2 ubiquitination in HA-Ub western blot analysis of flag-MAD2 immunoprecipitates, consistent with the observation that MAD2 levels are not altered upon RNF8 overexpression (Fig. 2H; Supplementary Fig. S2C). Given that MAD2 must be in a dimeric form to associate with RNF8 and that RNF8 competes with p31^comet^ for MAD2 binding, these data suggest that o-MAD2 may associate with the RNF8-c-MAD2 complex transiently/ weakly, which may increase MAD2-CDC20 complex formation via the MAD2 templating mechanism.

### RNF8 overexpression impairs GSC mitotic progression that is dependent on its FHA and RING domains

**Fig. 3:**
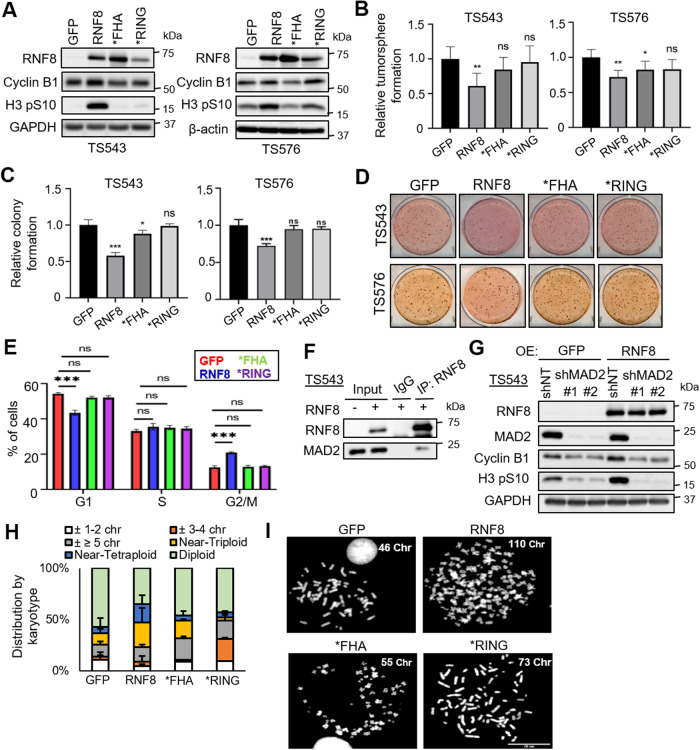
RNF8 overexpression impairs GSC mitotic progression that is dependent on its FHA and RING domains. **A** Western blot analysis of RNF8 and SAC markers, including H3 pS10 and cyclin B1, in GSCs transduced with different RNF8 constructs. RNF8 serves as the positive control whereas GAPDH and β-actin serve as loading control respectively. **B** Tumorsphere formation of two GSC lines overexpressing GFP, RNF8, *FHA, or *RING (*n* = 6) (mean±SD). *P < 0.05; **P < 0.01. **C**, **D** Colony formation of two GSC lines overexpressing GFP, RNF8, *FHA, or *RING (*n* = 4) (mean ± SD). *P < 0.05; ***P < 0.001. **D** Representative images of (**C**). **E** Cell cycle analysis of TS543 overexpressing GFP, RNF8, *FHA or *RING, with 40 µM Z-VAD-FMK treatment (72 h) (*n* = 3) (mean±SD). ***P < 0.001. **F** Western blot analysis of MAD2 levels in the RN**F**8 IP from RNF8 overexpressing GSC TS543 lysates. **G** Western blot analysis of H3 pS10 and cyclin B1 levels of GSC TS543 with GFP or RNF8 overexpression, with or without MAD2 depletion. RNF8 and MAD2 serve as the positive controls, while GAPDH serves as the loading control. **H**, **I** Karyotyping analysis of GSC TS543 overexpressing different RNF8 constructs (*n* = 3). Near triploidy: 60–80 chromosomes; near tetraploidy: 81–110 chromosomes. Minimum of 100 spreads were analyzed per condition. **I** Representative karyotypes from GSCs overexpressing different RNF8 constructs (**H**). Scale bar 20 μm.

To translate our findings of RNF8’s role in mitotic checkpoint into a physiologically relevant setting, we focus on GSCs that are known to be sensitive to mitotic checkpoint disruption and chromosomal instability. The overexpression of RNF8, but not the GFP control, *FHA or *RING, significantly reduced GSC tumorsphere and colony formation, while increasing G2-M frequency (Fig. 3A–E). In independent two GSC lines, the overexpression of RNF8, but not GFP control, *FHA or *RING, robustly increased the levels of H3 pS10 (although cyclin B1 levels only increased in TS543) (Fig. 3A). Notably, RNF8 overexpression did not increase H3 pS10 levels in non-cancerous human neural progenitor cells, underscoring the specificity of RNF8’s effect on GSCs (Supplementary Fig. S3A). The role of endogenous RNF8 in mediating mitotic checkpoint was further strengthened by showing that *RNF8* depletion diminished the increase in H3 pS10 levels that were afforded by nocodazole treatment of GSC (Supplementary Fig. S3B). Similar to that observed in 293T cells, MAD2 is also detected in the RNF8 immunoprecipitates of GSC lysates (Fig. 3F). In agreement with the crucial role of MAD2 in mediating RNF8’s mitotic checkpoint response in GSC, *MAD2* depletion abrogated the increase in H3 pS10 and cyclin B1 levels that was afforded by RNF8 overexpression (Fig. 3G). Cancer cells can exit mitotic arrest via a process known as mitotic slippage to avoid cell death, for instance by degrading cyclin B1, with the consequential increase in chromosomal instability and aneuploidy [30, 37]. As expected, the overexpression of RNF8, but not that of GFP control, *FHA or *RING, led to the greatest increase in GSC aneuploidy and chromosomal abnormalities (e.g., multi-nucleation and micronuclei formation) (Fig. 3H, I; Supplementary Fig. S3C–F). Furthermore, the treatment of RNF8 overexpressing GSC with 17-AAG (an established HSP90 inhibitor) led to the greatest reduction in cell viability and GSC marker expression when compared to single agent treatment (Supplementary Fig. S3G, H), indicating a heightened sensitivity to 17-AAG upon RNF8 overexpression, which is consistent with the view that aneuploid cells are more sensitive to proteotoxic stressors than normal cells [38]. We also considered whether the RNF8-induced mitotic checkpoint may be attributed to a defect in the repair of spontaneous DNA damage. To this end, we examined H3 pS10 levels upon RNF8 overexpression in GSC, with or without ATM inhibition using KU-55933 since ATM kinase activity is required for RNF8 activation in DDR. RNF8 overexpression resulted in increased DNA damage as reflected by elevated γH2AX levels, suggesting that RNF8 overexpression does not facilitate the repair of spontaneous DNA damage (Fig. S3I). This increase in DNA damage may in part be due to genomic instability (micronuclei formation) through mitotic slippage upon RNF8 overexpression. Although ATM inhibition slightly decreased H3 pS10 levels in the setting of RNF8 overexpression, H3 pS10 levels remained higher than that in the GFP control (Fig. S3I). Thus, RNF8 overexpression can activate mitotic checkpoint, independent of its role in DDR. Collectively, we show that RNF8 overexpression impairs GSC proliferation, mitotic progression and genomic integrity that is dependent on its FHA and RING domains.

### CAMK2D associates with RNF8’s FHA domain weakly/ transiently via p-Thr287 to mediate RNF8-induced mitotic checkpoint

**Fig. 4:**
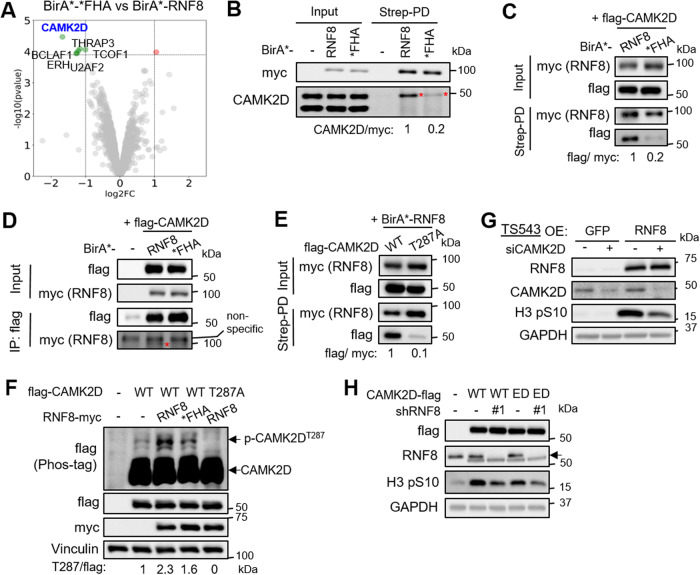
CAMK2D associates with RNF8’s FHA domain weakly/transiently via p-Thr287 to mediate RNF8-induced mitotic checkpoint. **A** Volcano plot showing the proteins which are significantly depleted in *FHA vs RNF8 proximal proteomes (i.e., minus log_2_fold change (FC)). **B** Western blot analysis of endogenous CAMK2D levels in the streptavidin pull down lysates of HEK293T overexpressing BirA*-RNF8 or *FHA. CAMK2D/myc ratio was normalized to BirA*-RNF8 group. Western blot analysis of flag-CAMK2D levels in the streptavidin pull down lysates (**C**), or flag IP (**D**) of HEK293T co-transfected with flag-CAMK2D and BirA*-RNF8/*FHA. The band of interest is indicated by *. **E** Western blot analysis of flag levels in streptavidin pulldown lysates from HEK293T cell lysates overexpressing flag-CAMK2D or -CAMK2D^T287A^ mutant, along with BirA*-RNF8. **F** Phos-tag SDS-PAGE analysis of flag-CAMK2D and -CAMK2D^T287A^ mutant, along with co-expression of EV or myc-tagged RNF8/*FHA. The p-T287/flag ratio was normalized to empty vector control. **G** Western blot analysis of H3 pS10 levels in GFP or RNF8 overexpressing GSC TS543, with or without CAMK2D KD. RNF8 and CAMK2D serve as positive control whereas GAPDH serve as the loading control. **H** Western blot analysis of H3 pS10 levels in wildtype CAMK2D or CAMK2D^ED^ overexpressing GSC TS543, with or without *RNF8* KD. Flag and RNF8 serve as the positive controls, while GAPDH serves as the loading control.

The observation that RNF8’s FHA domain plays a key role in eliciting the mitotic checkpoint response in GSC prompted us to compare the myc-BirA*-*FHA vs -RNF8 proximal proteomes using BioID-mass spectrometry analysis for the discovery of FHA domain-specific proteins. Interestingly, CAMK2D emerged as the top depleted protein in the *FHA vs RNF8 proximal proteomes (Fig. 4A; Supplementary Table S2). We validated this result by showing that both endogenous and exogenously expressed CAMK2D were preferentially enriched in the streptavidin pulldown lysates of RNF8 vs *FHA overexpressing 293T cells (Fig. 4B, C). Since RNF8 cannot be detected in the flag-CAMK2D immunoprecipitates, this suggests a weak/ transient RNF8-CAMK2D protein interaction (Fig. 4D). That CAMK2D binds to RNF8’s FHA domain was further strengthened by using CAMK2D^T287A^, which cannot be phosphorylated and thus binds poorly to RNF8 (NB: RNF8’s FHA domain binds to phospho-Thr peptides) [39, 40] (Fig. 4E, F). Unexpectedly, we found greater phosphorylation of CAMK2D upon RNF8 overexpression, which was reduced with *FHA, suggesting that the binding of RNF8 to CAMK2D has enhanced the kinase activity of CAMK2D (Fig. 4F) (NB: CAMKII proteins undergo autophosphorylation [41]). Next, we asked if CAMK2D may phosphorylate RNF8 at S157, which lies within a highly conserved CAMK2D motif (Supplementary Fig. S4A). Phos-tag analysis showed that RNF8 was indeed phosphorylated at S157 as the phospho band was absent with RNF8^S157A^, but not RNF8^T198A^ (previously linked to CDK1-mediated phosphorylation [42]) (Supplementary Fig. S4B). Notably, p-RNF8^S157^ was severely reduced with *FHA (about 90% reduction of wildtype level) (Supplementary Fig. S4B). To ascertain that RNF8 is a CAMK2D substrate, we co-expressed myc-RNF8 along with wildtype CAMK2D, catalytically inactive CAMK2D^ED^ or CAMK2D^T287A^, and assessed RNF8 phosphorylation using Phos-tag analysis. Only the overexpression of wildtype CAMK2D, but not CAMK2D^ED^ or CAMK2D^T287A^, dramatically increased p-RNF8^S157^ (about 5-fold) (Supplementary Fig. S4C). Conversely, the treatment of 293T cells with KN93 (a pan-CAMK2 inhibitor) blocked p-RNF8^S157^ (up to 70% reduction relative to DMSO control) (Supplementary Fig. S4D). To determine if p-RNF8^S157^ contributed to mitotic checkpoint, we overexpressed the RNF8^S157A^ phosphomutant in GSC and found that this did not block H3 pS10 increase (Supplementary Fig. S4E). Nonetheless, the genetic knockdown of CAMK2D abrogated the increase in H3 pS10 levels that were afforded by RNF8 overexpression in GSC (Fig. 4G). Furthermore, the overexpression of either wildtype CAMK2D or CAMK2D^ED^ increased H3 pS10 levels in GSC, and that *RNF8* depletion partially reduced H3 pS10 levels in both cases (Fig. 4H). Thus, our data support the binding of CAMK2D to RNF8, but not the kinase activity CAMK2D on RNF8 phosphorylation, to be critical for mitotic checkpoint activation in GSC.

### CAMK2D serves as a molecular scaffold for RNF8-MAD2 complex

**Fig. 5:**
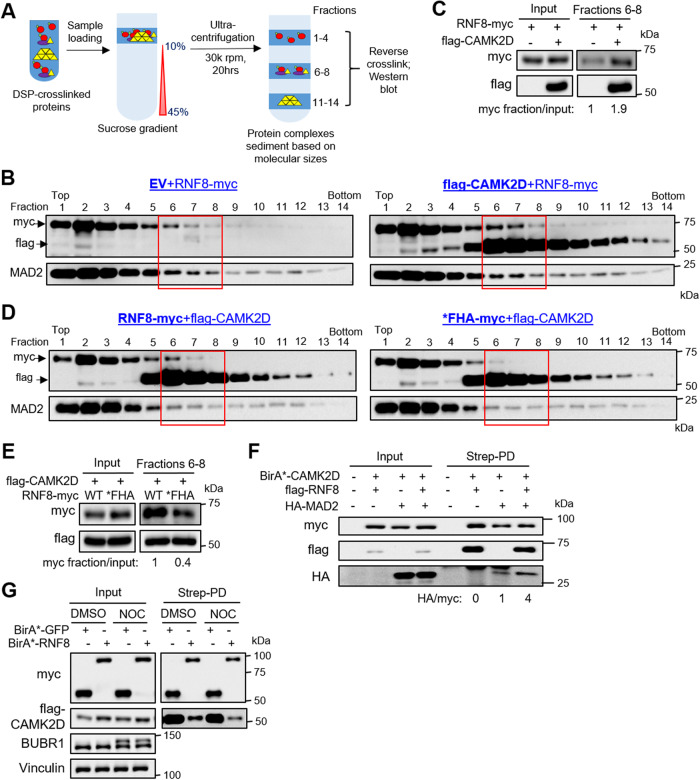
CAMK2D serves as a molecular scaffold for RNF8-MAD2 complex. **A** Schematic diagram to illustrate the workflow for sucrose density gradient ultracentrifugation to assess RNF8 protein distribution in various fractions. **B** Western blot analysis of myc-RNF8, flag-CAMK2D, and MAD2 levels, with or without CAMK2D overexpression, in various fractions after sucrose density gradient ultracentrifugation of 293T cell lysates. **C** Western blot analysis of myc-RNF8 levels, with or without CAMK2D overexpression, in pooled fractions 6 to 8 from (**B**). The myc/input ratio was normalized to the EV control. **D** Western blot analysis of myc-RNF8/*FHA, flag-CAMK2D and MAD2 levels, with CAMK2D overexpression, in various fractions after sucrose density gradient ultracentrifugation of 293T cell lysates. **E** Western blot analysis of myc-RNF8/*FHA and MAD2 levels, with CAMK2D overexpression, in pooled fractions 6–8 from (**D**). The myc/input ratio was normalized to the RNF8 control. **F** Western blot analysis of flag-RNF8 and HA-MAD2 levels in the streptavidin pull down lysates of HEK293T overexpressing BirA*-CAMK2D, along with or without flag-RNF8 and HA-MAD2 overexpression. **G** Western blot analysis of flag-CAMK2D levels in the streptavidin pulldown lysates of BirA*-RNF8 or GFP-overexpressing HEK293T, with or without 200 ng/ml NOC (16 h).

Besides serving as a kinase for specific substrates, CAMKII proteins can also confer a scaffolding role by bundling F-actin for the maintenance of dendritic spine structure [43]. Since the kinase activity of CAMK2D is not critical for RNF8-mediated mitotic checkpoint, we explored the possibility that CAMK2D may act as a scaffold for the RNF8-MAD2 complex. To test this hypothesis, we evaluated the distribution of RNF8, with or without CAMK2D overexpression, across a 10–45% sucrose gradient after ultracentrifugation which would allow us to fractionate DSP-crosslinked protein complexes based on molecular sizes (Fig. 5A). Notably, CAMK2D overexpression increased RNF8 levels in fractions 6–8, which likely corresponds to higher order CAMK2D structures (Fig. 5B, C) (NB: CAMKII proteins can form dodecamers [44]). Conversely, there was less *FHA (which binds poorly to CAMK2D) detected in fractions 6–8 when compared to RNF8 using analogous experimental setup (Fig. 5D, E). Next, we addressed whether CAMK2D, RNF8 and MAD2 can exist as a protein complex by employing a CAMK2D proximity labeling approach, given the weak/ transient CAMK2D-RNF8 interaction (Fig. 4C, D). As expected, there were more HA-MAD2 proteins detected in the CAMK2D proximal proteome with the co-expression of RNF8, indicating that RNF8 facilitates MAD2 binding to CAMK2D (Fig. 5F). Similar to MAD2, the RNF8-CAMK2D interaction could be detected in asynchronous and mitotic arrested cells, suggesting that the CAMK2D-RNF8-MAD2 complex may exist throughout the cell cycle (Fig. 5G). Taken together, we show that CAMK2D serves as a molecular scaffold for the RNF8-MAD2 complex.

### Gliomas with high HER2-EGFR signaling tend to avoid high RNF8 expression as RNF8 overexpression impedes GSC tumorigenicity

**Fig. 6:**
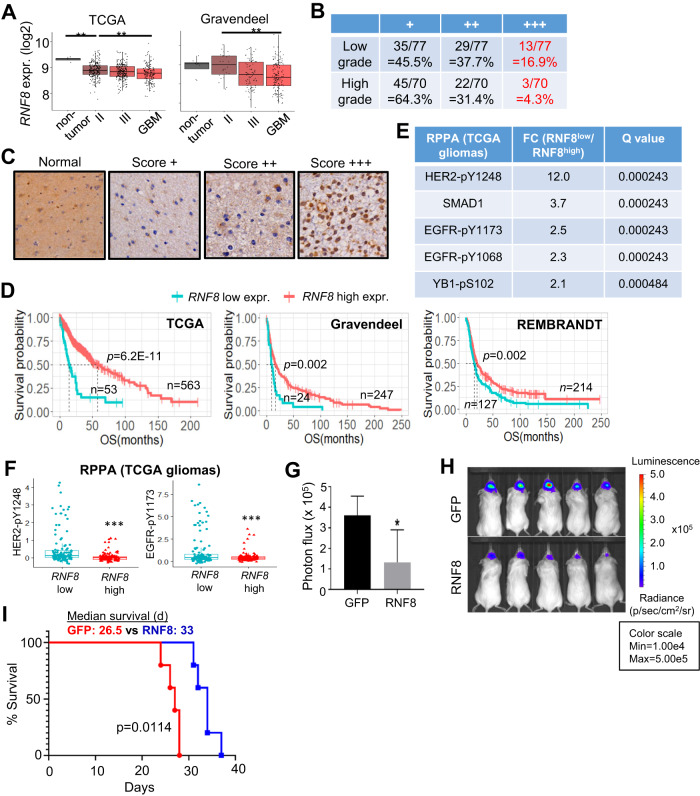
GBM with high HER2-EGFR signaling tends to avoid high RNF8 expression as RNF8 overexpression impedes GBM tumorigenicity. **A** Correlative analysis of *RNF8* mRNA levels with glioma grades in two independent glioma patient cohorts. Wilcoxon-Mann-Whitney exact test. **P < 0.01. **B**, **C** Immunohistochemistry (IHC) scores of RNF8 in different grades of gliomas using glioma patient tissue microarray. **C** Representative images of the IHC scores of RNF8 in (**B**). **D** Correlative analysis of *RNF8* mRNA levels with glioma patient survival in multiple glioma patient cohorts. OS: overall survival. Wald test. **E** Top 5 proteins that are upregulated in RNF8^low^ vs RNF8^high^ gliomas in the TCGA RPPA dataset using GlioVis (http://gliovis.bioinfo.cnio.es/). **F** RPPA analysis of HER2-pY1248 and EGFR-pY1173 in RNF8^low^ versus RNF8^high^ gliomas using TCGA cohort. ***P < 0.001, t-test. **G**, **H** In vivo bioluminescence-based imaging of post-orthotropic injection of TS543 overexpressing GFP or RNF8. **H** Representative images of the tumor-bearing NSG mice in (**G**) (*n* = 5) (mean±SD). *P < 0.05. **I** Kaplan Meier curves of mice implanted with GFP or RNF8 overexpressing TS543. Log rank test.

Next, we interrogated the relevance of RNF8 dysregulation in gliomagenesis. We observed significantly lower *RNF8* expression in GBM when compared to the non-tumor tissues (Fig. 6A). That high-grade gliomas (including GBM) tend to avoid high RNF8 expression was further validated at the protein level using glioma microarray (~4-fold less; score +++: 4.3% high-grade vs 16.9% low-grade) (Fig. 6B, C). Consistently, low *RNF8* expression portended inferior patient outcome in multiple glioma cohorts (Fig. 6D). As *RNF8* dysregulation appears to be more strongly linked to a subset of gliomas, we explored The Cancer Genome Atlas (TCGA) reverse phase protein array (RPPA) dataset to identify molecular attributes that are associated with RNF8^low^ tumors. This revealed that RNF8^low^ gliomas expressed significantly higher levels of HER2-pY1248 and EGFR-pY1173 than RNF8^high^ gliomas, consistent with the role of receptor tyrosine kinase signaling being a core regulatory circuitry in gliomagenesis (Fig. 6E, F) [45]. Importantly, there was no significant correlation between *RNF8* mRNA levels and the protein levels of DDR markers in the TCGA RPPA dataset, including pCHK2_pT68, pCHK1_pS296, pCHK1_pS345, 53BP1 and MRE11, suggesting that RNF8’s prognostic value in glioma may not be related to its role in DDR (Fig. S5A). In agreement with the clinical data supporting a tumor-suppressing role of RNF8 in gliomagenesis, xenotransplantation experiments showed that RNF8 overexpressing GSCs generated significantly smaller tumor volume than their GFP counterpart in immunocompromised mice, which was accompanied by an extended survival (median survival of 33 vs 26.5 days) (Fig. 6G–I). Thus, a subset of gliomas that is associated with high HER2-EGFR signaling preferentially downregulates RNF8 expression as RNF8 overexpression impedes GSC tumorigenicity.

### Indirectly activating the RNF8-associated mitotic checkpoint using a PLK1i synergizes with HSP90i to reduce GSC proliferation and stemness

**Fig. 7:**
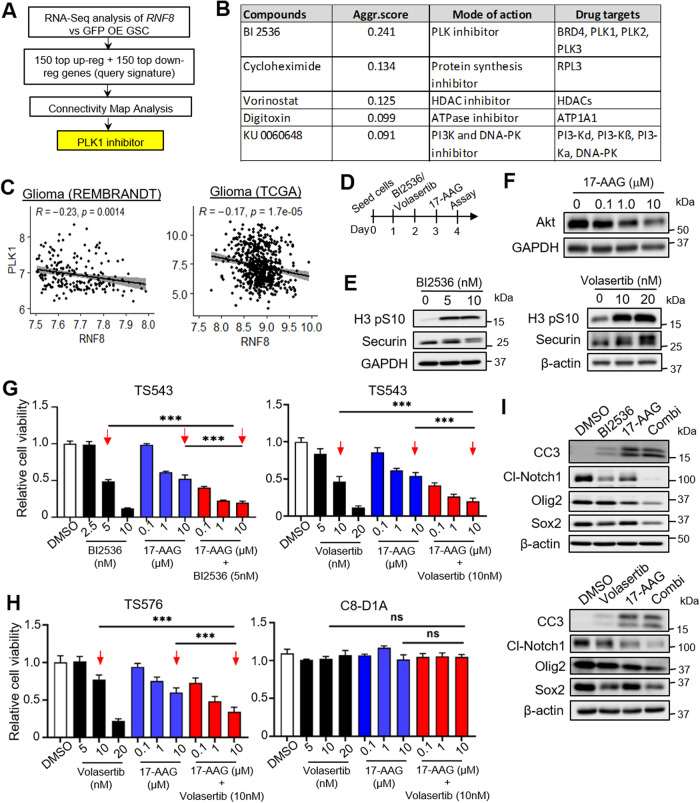
CMA using the RNF8 overexpression gene signature identifies PLK1i that synergizes with HSP90i to reduce GSC proliferation and stemness in vitro. **A** CMA using a RNF8 overexpression query signature identifies PLK1 inhibitor. **B** Top 5 compounds identified with CMA by using the RNF8 overexpression query signature. **C** Correlative analysis of *PLK1* and *RNF8* mRNA levels in glioma patients. **D** Schematic diagram showing the drug treatment regimen to be used in (**G–I**). **E** Western blot analysis of H3 pS10 and securin levels in TS543 lysates upon treatment with DMSO or BI 2536/Volasertib at the indicated concentrations for two days. GAPDH and β-actin serve as loading controls. **F** Western blot analysis of Akt levels in TS543 lysates upon treated with DMSO or 17-AAG at the indicated concentrations for 1 day. GAPDH serves as loading control. **G** Cell viability assay of TS543 upon treatment with BI 2536/Volasertib, 17-AAG, or combination of BI 2536/ Volasertib and 17-AAG at the indicated concentrations (*n* = 6) (mean ± SD). ***P < 0.001. **H** Cell viability assay of TS576 and non-cancerous mouse astrocytes upon treatment with Volasertib, 17-AAG, or Volasertib/17-AAG combination at the indicated concentrations (*n* = 6) (mean ± SD). ***P < 0.001. **I** Western blot analysis of cleaved-caspase 3 (CC3) and select GSC stemness marker levels in TS543 lysates upon treatment with BI 2536/ Volasertib, 17-AAG, or combination of BI 2536/ Volasertib and 17-AAG. β-actin serves as loading control.

Given that no small molecule RNF8 activators exist, we explored pharmacologic agents that mimicked RNF8 overexpression by using CMA [46, 47] and identified BI2536 (an established PLK1 inhibitor) as the top hit (Fig. 7A, B; Supplementary Table S3). Reassuringly, *RNF8* expression is also significantly anti-correlated with that of *PLK1* in multiple glioma datasets, supporting our CMA hit (Fig. 7C). Since *RNF8* overexpression induces GSC aneuploidy and sensitizes GSC to 17-AAG (Fig. 3H; Supplementary S3G), we sought to determine if pretreatment with PLK1 inhibitors, including BI2536 and volasertib, would similarly synergize with 17-AAG to mitigate GSC proliferation and stemness. Strikingly, the pretreatment of GSC with either BI2536 or volasertib for two days, followed by a one day 17-AAG treatment, led to a synergistic reduction in cell viability and GSC stemness marker expression when compared to single agent treatment, similar to that observed with RNF8 overexpression (Fig. 7D–I; Supplementary Fig. S3G, H). In contrast, there was no significant change in the cell viability of non-cancerous mouse astrocytes even with the volasertib/ 17-AAG combination (Fig. 7H). These in vitro data provide proof-of-concept that the PLK1/HSP90 inhibitor combination may be further developed for treating RNF8^low^ GBM in the clinic.

## Discussion

Based on our mechanistic insights, we propose the following model to describe the molecular basis of the CAMK2D-RNF8-MAD2 complex in mitotic checkpoint regulation of GSC (Supplementary Fig. S6A). From our RNF8 proximity proteomics, we subsequently provide evidence that RNF8 competes with a small pool of p31^comet^ for MAD2 binding, and that RNF8’s RING domain likely binds to the c-MAD2-o-MAD2 dimer (Figs. 1 and 2). Equally crucial for mitotic checkpoint is the transient/weak interactions between the p-Thr287 of CAMK2D and RNF8’s FHA domain (Fig. 4B, D). Although CAMK2D binds to and phosphorylates RNF8 at S157, the kinase activity of CAMK2D is dispensable for the RNF8-induced mitotic checkpoint (Fig. 4H; Supplementary Fig. S4E). Using sucrose gradient co-sedimentation analysis to assess the co-migration profiles of RNF8 and CAMK2D, we found that the majority of CAMK2D resides in fractions 6 to 8, and CAMK2D overexpression increases the amount of RNF8 in these fractions in a FHA-dependent manner, indicating that CAMK2D interacts with RNF8 as oligomers and plays a scaffolding role for RNF8 (Fig. 5A–E) (NB: CAMKII forms dodecamers in cells [44]). Such a scaffolding function of CAMKII has also been reported in synapses, including how CAMKII-α acts to recruit proteasomes to the post-synaptic density to regulate synaptic protein degradation; and how CAMKII-β plays a structural role in maintaining synaptic structure by regulating F-actin bundling [48, 49].

Interestingly, the overexpression of RNF8, but not *FHA, increases the phosphorylation of CAMK2D at T287 (Fig. 4D). The T287 residue lies within the regulatory loop domain of CAMK2D, which associates with the kinase domain to block substrate access [41, 50]. Upon an increase in intracellular calcium (Ca^2+^) levels, the Ca^2+^-calmodulin complex binds to the regulatory loop to relieve the inhibitory intramolecular interactions, thereby allowing CAMK2D to transphosphorylate its adjacent subunits at T287 residue to achieve autonomous activity [41, 50]. Thus, the binding of RNF8 to CAMK2D at p-Thr287 may lock CAMK2D in an active state, hence augmenting its ability to autophosphorylate. Consistent with this idea, the binding of the C-terminal tail of NMDA receptor to CAMKII has been reported to activate CAMKII’s kinase activity through occupying the T site regions [51].

We also considered the temporal and spatial regulation of the CAMK2D-RNF8-MAD2 complex for its role in mitotic checkpoint. Our data showed that RNF8 is likely to interact with CAMK2D and MAD2 throughout the cell cycle (Figs. 1D, 5G). This is further supported by a previous study that found no change in T287 phosphorylation on CAMK2D throughout the cell cycle, although intracellular Ca^2+^ levels oscillate [52–54]. The majority of CAMK2D isoforms display cytoplasmic localization, besides CAMKIIδ_B_ that contains a nuclear localization signal within the linker region [55, 56]. Based on the Human Protein Atlas ((https://www.proteinatlas.org/), malignant glioma tissues showed CAMK2D localization to the cytoplasm. Thus, we propose that CAMK2D interacts with the RNF8-MAD2 complex in the cytoplasm to elicit mitotic checkpoint. Consistent with this idea, RNF8 localizes to the cytoplasm where it interacts with HERC2 and NEURL4 in neurons, IKKα/β, as well as Akt in lung cancer [57–59]. Similarly, MAD2 is also present in the cytoplasm where it interacts with p31^comet^ during interphase [60, 61].

In contrast to the well-established roles of RNF8 in regulating its substrates through ubiquitination, our study revealed an E3 ligase-independent function of RNF8 in activating the mitotic checkpoint. Our data support a role of RNF8’s RING domain in selecting the type of MAD2 conformer for stable interaction, instead of its canonical E3 ligase function (Fig. 2). It is not uncommon for E3 ubiquitin ligases to exert their function independently of their ligase activity. For instance, HERC3 directly associates with β-TrCP to prevent it from binding to YAP/TAZ, leading to increased protein stability of YAP/TAZ [62]. Another E3 ligase, HACE1, promotes the ubiquitination-independent proteosomal degradation of Spindlin-1 by functioning as a molecular scaffold, which bridges Spindlin-1 and 20S proteasome [63]. Hence, our findings provide yet another example of an E3 ligase that may affect its client protein function without effecting substrate ubiquitination, highlighting the biological complexity of E3 ubiquitin ligases.

While a previous study proposed a role of RNF8 in promoting GBM tumorigenicity by mediating histone H3 ubiquitination and degradation [64], we find that RNF8 mRNA and protein levels are preferentially downregulated in gliomas, particularly those that are associated with high HER2-EGFR signaling. RNF8 overexpression interrupted GSC cell cycle progression, leading to mitotic slippage-associated chromosomal instability and impaired tumorigenesis. Although no RNF8 activators currently exist, our CMA approach identified PLK1 inhibitor that mimics RNF8 overexpression in GSC. Volasertib has shown remarkable efficacy as monotherapy or chemo/radiosensitizers in preclinical models of GBM [65–67]. However, it has achieved limited success in GBM clinical trials due to poor blood-brain-barrier penetrance [66, 68]. To improve the potency of PLK1 inhibitors, we provide proof-of-concept that a drug combination involving volasertib pretreatment, followed by 17-AAG administration, synergistically impairs GSC viability and stemness in vitro due to volasertib-induced aneuploidy that increases GSC sensitivity to proteotoxic stressors (Fig. 7; Supplementary Fig. S6A). Moving forward, we envision the development of GBM-specific nanocarriers that effectively deliver the volasertib/17-AAG combination as an alternative treatment option for RNF8^low^ GBM [69, 70]. In summary, our work highlights RNF8 downregulation as a strategy exploited by GSC to prevent mitotic checkpoint, and more importantly, a previously unrecognized CAMK2D-RNF8-MAD2 complex that can generate mitotic checkpoint signal in glioma.

## Materials and methods

### Cell lines and compounds

Patient-derived GSCs and human neural progenitor cells (hNPC) were cultured in human neural stem cell maintenance media (Millipore), 1% penicillin-streptomycin (PS), and supplemented with EGF and β fibroblast growth factor (20 ng/ml each). hNPC was induced from human embryonic stem cells H1 as described previously [71]. Noncancerous mouse astrocytes (C8-D1A) were cultured with Dulbecco’s modified Eagle’s medium/nutrient mixture F12 (DMEM/F12) with 10% fetal bovine serum (FBS) and 1% PS. Human embryonic kidney (HEK) 293T cells were cultured with DMEM-high glucose with 10% FBS and 1% PS. The following inhibitors were used in this study: 17-AAG (MedChemExpress), Z-VAD-FMK (MedChemExpress), KN-93 (MedChemExpress), Volasertib (Selleckchem), BI 2536 (MedChemExpress), KU-55933 (MedChemExpress), and Nocodazole (Sigma).

### Tumorsphere formation assay

The tumorsphere formation assay involved seeding transduced GSCs at a density of 1–2 cell per µl, and the number of tumorspheres in each well was quantified after 5 days. Data presented are from six replicates.

### Anchorage independent growth assay

Anchorage-independent growth assays were performed in replicates of 4 in 6-well plates. Transduced GSCs were seeded (1 × 10^4^ cells per well) in stem cell proliferation media with EGF and FGF containing 0.5% low-melting agarose on the top of bottom agar containing 1% low-melting agarose stem cell proliferation media with EGF and FGF. After 14–21 days, colonies were stained with Iodonitrotetrazolium chloride (Sigma) and counted.

### Lentiviral transduction

Lentiviruses were generated by co-transfecting HEK293T cells with pMD2.G, pRSV-Rev, pMD-VSVG and overexpression/shRNA plasmids. The media was collected 72-hour post-transfection, concentrated using ultracentrifugation (Optima XL-100K) and the lentiviral particles were resuspended in DMEM/F12. GSCs were transduced with lentivirus in the presence of 0.4 µg/ml polybrene (Sigma) and the knockdown efficiency was validated using Western blot analysis 72-h post-transduction. shRNAs against human MAD2 (shMAD2#1, TRCN0000273382 and shMAD2#2, TRCN0000006566) and human RNF8 (shRNF8#1, TRCN0000003438 and shRNF8#2, TRCN0000003441) were purchased from Sigma.

### RNA interference

siRNAs against human CAMK2D (J-004042-08-0005 and J-004042-11-0005) were purchased from Dharmacon. The siRNAs were pooled and transfected into GSCs using Lipofectamine RNAiMAX (Invitrogen) according to the protocol specified by the manufacturer. After 72 h, the cells were harvested and knockdown efficiency was analyzed using Western blot.

### Plasmid construction

pHAGE-EF1a-RNF8-IRES-GFP lentiviral construct was obtained from DePinho lab. pHAGE-*FHA and -*RING were generated using QuickChange II site-directed mutagenesis kit. pHAGE-S157A mutant was generated using Q5 site-directed mutagenesis kit. myc-tagged RNF8, *FHA and *RING were generated by cloning the respective open reading frames (ORFs) into pcDNA4.1/TO/myc-HisA vector. flag-tagged RNF8, MAD2, p31^comet^, Cdc20 and CAMK2D were generated by cloning the respective ORFs into pCMV-Tag2B vector. HA-tagged MAD2 and p31^comet^ were generated by cloning the respective ORFs into pCMV4-HA vector. BirA*-GFP, RNF8, *FHA, *RING, -CAMK2D were generated by cloning the respective ORFs into pcDNA3.1-myc-BioID vector. myc-RNF8^3A^, -RNF8^4A^, -RNF8^S157A^, -RNF8^T198A^, flag-p31^QF^, HA-p31^S102A^, flag-MAD2^RQ^, flag-CAMK2D^ED^, and –CAMK2D^T287A^ mutants were generated using overlap extension PCR-based site-directed mutagenesis. flag-tagged CAMK2D or CAMK2D^ED^ overexpressing lentiviral vectors were generated by cloning the respective ORFs into plenti-c-MYC-DDK-P2A-Puro vector (Origene). HA-MAD2^WT^, -MAD2^∆C^, and -MAD2^L13A^ were kindly provided by Song-Tao Liu [34]. HA-Ub expressing plasmid was a gift from Han-Ming Shen lab. The primers used for cloning were listed in Supplementary Table S4.

### Cell viability assay

GSCs were seeded on 96 wells and transduced or treated with BI 2536/Volasertib. After 48-h post-transduction/drug treatment, the cells were treated with 17-AAG for 24 h. The cell viability was measured using the CellTitre-Glo luminescent cell viability assay (Promega) as specified by the manufacturer’s instructions. The experiments were performed with six replicates and the data was normalized to the DMSO control.

### Propidium iodide (PI) staining

GSCs were transduced with lentiviruses overexpressing GFP, RNF8, *FHA or *RING and fresh media containing 40 µM Z-VAD-FMK (MedChemExpress) was replenished after 4-hour post-transduction. After 72 h, the transduced cells were harvested, washed with ice-cold 1× PBS and fixed in 70% ethanol overnight at 4 °C. Cells were washed with ice-cold 1× PBS and resuspended in PI (Sigma) staining solution (50 µg/mL PI, 0.3% Triton-X100, 1 mg/mL RNaseA) and incubated at room temperature for 45 min. PI positive cells were sorted using Fortessa Cell Analyzer and cell cycle distribution were analyzed using FlowJo software. Data presented was calculated from three replicates.

### Karyotyping

Transduced GSC TS543 cells were grown in medium supplemented with 0.1 μg/ml colcemid (KaryoMAX, Gibco) for 2–3 h. Mitotic cells were collected by shake-off, washed thoroughly in PBS and processed for fixation. Cells were pelleted and re-suspended in a hypotonic solution of 0.075 M KCl for 18 min, fixed in Carnoy’s fixative (3:1 methanol:glacial acetic acid) and washed four times with Carnoy’s fixative. All fixed samples were spread on slides by dropping from a height of 0.5–1 cm and subsequently dried completely on a hotplate at 37 °C. Samples were then stained with Hoechst and visualized by wide-field microscopy for chromosome number counts.

### Nuclear abnormality scoring

TS543 cells were seeded on cover slips and transduced with GFP, RNF8, *FHA, and *RING overexpression lentivirus. Transduced cells were fixed with 4% PFA and were stained with Hoechst 3342 (Invitrogen). Cover slips were mounted into slides and images were acquired at 40× objective on Nikon ECLIPSE Ti microscope. Cells were categorized based on specific types of nuclear abnormality. Micronucleated cells may contain one or multiple nuclear compartments that are external to the primary nucleus. External nuclear compartments that are less than 1/3 of the size of the main nucleus are considered as micronucleus. Multinucleated cells contain multiple nuclear compartments that are of about equal sizes. These cells may also contain micronuclei. At least 200 cells were scored for each condition.

### Intracranial tumor formation in vivo

GSCs (1 × 10^5^ viable cells) were grafted intracranially into NSG (NOD scid gamma mouse) mice (InVivos) aged 6–8 weeks. Tumor incidence was determined at indicated time points by luciferase imaging of mice using Xenogen IVIS (PerkinElmer) according to the manufacturer’s instructions. Animals were maintained until neuro-logical signs were apparent, at which point they were euthanized. All mouse manipulations were performed with the approval of National University of Singapore Institutional Animal Care and Use Committee.

### Immunoblotting

Cell pellets were lysed with RIPA buffer (Thermo Scientific) supplemented with protease inhibitor and phosphatase inhibitor cocktails (Roche). Equal amount of lysates were loaded into each well and the samples were resolved by SDS-PAGE, followed by transfer onto nitrocellulose membrane (Bio-Rad) for antibody incubation, and the blots were developed using the ChemiDoc imaging system (Bio-Rad). Information about the primary antibodies was listed below: RNF8 (Santa Cruz, sc-271462); MAD2 (Bethyl Laboratories, A300-301A); MAD2 (BD Transduction Laboratories, 610678); p-H3 (Ser10) (Cell Signaling Technology, 3377S); p31^comet^ (Millipore, MABE451); Myc-tag (Cell Signaling Technology, 2276S); Flag-M2 (Sigma, F1804); HA-tag (Cell Signaling Technology, 3724S); HA-tag (Proteintech, 51064-2-AP); GAPDH (Cell Signaling Technology, 2118S); Vinculin (Sigma, V9131); CAMK2D (Santa Cruz, sc-100362); CAMK2D (Proteintech, 20667-1-AP); Akt (pan) (Cell Signaling Technology, 4691S); Olig2 (Millipore, AB9610); Sox2 (abcam, ab97959); cleaved Notch1 (Cell Signaling Technology, 4147S); cleaved caspase 3 (Asp175) (Cell Signaling Technology, 9661S); β-actin (Sigma, A5316); BubR1 (BD Transduction Laboratories, 61250); Cyclin B1 (Cell Signaling Technology, 4135S); Securin (abcam, ab3305). γH2AX (Millipore, 07-164); p-Chk2 (Thr68) (Cell Signaling Technology, 2197); Chk2 (Cell Signaling Technology, 2662).

For the analysis of phosphorylation, proteins were resolved on polyacrylamide gels containing Phos-tag (Wako) and MnCl_2_.

### Immunoprecipitation

Cells were lysed in IP lysis buffer (50 mM Tris, 150 mM NaCl, 1% Triton X-100) supplemented with protease inhibitor and phosphatase inhibitor cocktails (Roche). 2–3 mg of lysates were pre-cleared with protein A/G agarose beads (Santa Cruz) for 30 min. Pre-cleared lysates were incubated with antibody together with Protein A/G agarose beads (Pierce^TM^) overnight at 4 °C with shaking. The beads were washed four times with IP wash buffer (50 mM Tris, 150 mM NaCl, 1% Triton X-100) and boiled with 2× SDS sample loading buffer. The samples were analyzed using Western blot.

### BioID and streptavidin pulldown assay

HEK293T cells were transfected with BioID vector (pcDNA3.1-myc-BioID) expressing BirA^*^-GFP, RNF8, *FHA or *RING respectively. After 48-hour post-transfection, cells were replenished with fresh media containing 50 µM biotin (Sigma) and incubated for 24 h. Transfected cells were lysed in IP lysis buffer and 2–3 mg of lysates were aliquoted for the streptavidin pull down assay. The lysates were incubated with streptavidin agarose beads (Pierce^TM^) overnight at 4 °C with gentle rotation. The beads were washed four times with IP wash buffer and streptavidin bound proteins were eluted with 2× SDS sample loading dye. The samples were analyzed using Western blot.

### Tissue microarray immunohistochemistry

Brain tumor tissue arrays (GL2083c and GL806f, US Biomax) were used to correlate RNF8 expression with tumor grades. Immunohistochemistry (IHC) analysis of RNF8 expression was performed with anti-RNF8 antibody (Proteintech, 14112-1-AP).

### In silico docking and molecular dynamics simulations

Available experimental atomic structures of MAD2 in its open (referred to as MAD2^O^) (pdb code: 1DUJ), and closed (referred to as MAD2^C^) (pdb code: 2V64) conformations and the E3 ligase domain of RNF8 (referred to as RNF8^E3^) (residues 391 to 480; pdb code: 4WHV) were used for the Molecular Dynamics (MD) simulations and in-silico docking calculations. Protein-protein docking was carried out with the program HADDOCK [23]. To build a complex between MAD2^C^ and RNF8^E3^ the binding region on MAD2^C^ was defined from residues from 195 to 205 at its C- terminus, while all the exposed residues in RNF8^E3^ were defined as its binding site.

Molecular Dynamics (MD) simulations were carried out on the modeled MAD2^C^–RNF8^E3^ complexes. The Xleap module of AMBER 18 was used to prepare the system for the MD simulations.  The simulation system was neutralized with appropriate numbers of counter ions.  The neutralized system was solvated in an octahedral box with TIP3P [51] water molecules, leaving at least 10 Å between the solute atoms and the boundaries of the box.  MD simulations were carried out with the pmemd.cuda module of the AMBER 18 package in combination with the ff14SB force field [52]. MD simulations were carried out in explicit solvent at 300 K. During the simulations the long-range electrostatic interactions were treated with the particle mesh Ewald [53] method using a real space cut off distance of 9 Å. The settle [54] algorithm was used to constrain bond vibrations involving hydrogen atoms, which allowed a time step of 2 fs during the simulations. Solvent molecules and counter ions were initially relaxed using energy minimization with restraints on the protein atoms. This was followed by unrestrained energy minimization to remove any steric clashes.  Subsequently the system was gradually heated from 0 to 300 K using MD simulations with positional restraints (force constant: 50 kcal mol^−1^ Å^−2^) applied to the protein atoms over a period of 0.25 ns allowing water molecules and ions to move freely followed by gradual removal of the positional restraints and a 2 ns unrestrained equilibration at 300 K. The resulting system was used as the starting structure for the production phase and three independent (using different initial random velocities) MD simulations were carried out for 100 ns each. Simulation trajectories were visualized using VMD and figures were generated using Pymol.

### Identification of phosphorylation sites by mass spectrometry

HEK293T cells were co-transfected with plasmids expressing myc-tagged RNF8 and flag-tagged p31^comet^. After 72-hour post-transfection, the cells were collected, washed with 1x PBS and lysed in IP lysis buffer. flag-p31^comet^ was immunoprecipitated using flag-M2 antibody and eluted with 2× SDS sample loading dye. The flag immunoprecipitate was resolved using SDS-PAGE and the polyacrylamide gel was stained with 0.25% Coomassie Brilliant Blue G-250 (Bio-Rad) to visualize the protein bands. The protein band corresponding to the molecular weight of flag-p31^comet^ was excised, trypsin digested and analyzed by liquid chromatography-mass spectrometry. The data were processed using ProteinPilot 5.02 (SCIEX) and visualized using PEAKS Viewer.

### Proteomics sample preparation

RNF8 (GFP vs RNF8 and RNF8 vs *FHA) samples were prepared for proteomics analysis from the streptavidin pull down lysates of HEK293T cells transfected with BirA*-GFP, -RNF8, or -*FHA. The streptavidin agarose beads were first washed four times with IP wash buffer and thrice with PBS. The supernatant was removed completely and the beads were then resuspended in 50% (v/v) trifluoroethanol (TFE) in 50 mM triethylammonium bicarbonate (TEAB), pH 8.5 containing 10 mM final concentration of tris(2-carboxyethyl)phosphine (TCEP) and incubated for 20 min at 55 °C for disulfide bridge reduction. Samples were cooled to 25 °C and alkylated with 55 mM 2-chloroacetamide in the dark for 30 min, followed by on-bead digestion with endoproteinase LysC (2 µg final amount) for 3 h and subsequently by trypsin (2 µg final amount) at 37 °C overnight. Once completed, beads were removed and the peptides were transferred to new tubes. Digestion was terminated by adding 1% (v/v) final concentration of trifluoroacetic acid to the samples, followed by desalting using C18 StageTips. Desalted peptides were dried by centrifugal evaporation, resuspended in 25 µl of TEAB, pH 8.5, and individually labeled using isobaric 6-plex tandem mass tags (TMT6-plex, Thermo Fisher Scientific) at 25 °C overnight. For GFP vs RNF8 samples, only 126, 127N, 128C, 130C, and 131 tags were used. For RNF8 vs *FHA, TMT-127N, 127C, 128C, 130C, and 131 tags were used. After labeling was completed, the reaction was quenched by addition of 30 µl of 1 M ammonium formate, pH 10 into each tube before pooling the samples into a new low-binding 1.5-ml microfuge tube. Pooled sample was desalted and fractionated on a self-packed spin column containing C18 beads (Dr Maisch GmbH) using 14%, 18%, 21%, 24%, 27%, 32%, and 60% acetonitrile in 10 mM ammonium formate, pH 10 as the step gradients. Fractions were dried by centrifugal evaporation and further washed and dried twice by addition of 60% acetonitrile in 0.1% formic acid to further remove residual ammonium formate salts.

### BioID interactomics by tandem mass spectrometry analysis

Dried fractions were resuspended in 10 µl of 2% (v/v) acetonitrile containing 0.06% (v/v) trifluoroacetic acid and 0.5% (v/v) acetic acid and transferred to an autosampler plate. Online chromatography was performed in an EASY-nLC 1000 (Thermo Fisher Scientific) liquid chromatography system using a single-column setup and 0.1% formic acid in water and 0.1% formic acid in 99% acetonitrile as mobile phases. Fractions were injected and separated on a reversed-phase C18 analytical column (Easy-Spray, 75 µm inner diameter ×50 cm length, 2 µm particle size, Thermo Fisher Scientific) maintained at 50 °C and using a 2–33% (v/v) acetonitrile gradient over 55 min, followed by an increase to 45% over the next 5 min, and to 95% over 5 min. The final mixture was maintained on the column for 4 min to elute all remaining peptides. Total run duration for each sample was 70 min at a constant flow rate of 300 nl/min.

For GFP vs RNF8 samples, data were acquired using an Orbitrap Fusion Lumos mass spectrometer (Thermo Fisher Scientific) using data-dependent mode. Samples were ionized using 2.1 kV and 300 °C at the nanospray source. Positively-charged precursor signals (MS1) were detected using an Orbitrap analyzer set to 60,000 resolution, automatic gain control (AGC) target of 800,000 ions, and maximum injection time (IT) of 50 ms. Precursors with charges 2–7 and having the highest ion counts in each MS1 scan were further fragmented using higher-energy collision dissociation (HCD) at 42% normalized collision energy. Fragment signals (MS2) were analyzed by the Orbitrap analyzer at a resolution of 50,000, AGC of 100,000 and maximum IT of 80 ms. Precursors used for MS2 scans were excluded for 45 s to avoid re-sampling of high abundance peptides. The MS1–MS2 cycles were repeated every 3 s until completion of the run. For RNF8 vs *FHA, data was also acquired on Orbitrap Fusion Lumos and acquisition settings were identical except for the following: MS1 AGC target was set to 400,000 and maximum IT at 100 ms; and MS2 AGC target at 75,000 and maximum IT at 300 ms.

### Proteomics data analysis

Proteins were identified using Proteome Discoverer™ (v2.4, Thermo Fisher Scientific). Raw mass spectra were searched against human primary protein sequences retrieved from Swiss-Prot (11 June 2019). Carbamidomethylation on Cys and TMT6-plex on Lys and N-terminus were set as a fixed modification; deamidation of asparagine and glutamine, acetylation on protein N termini, and methionine oxidation were set as dynamic modifications for the search. Trypsin/P was set as the digestion enzyme and was allowed up to three missed cleavage sites. Precursors and fragments were accepted if they had a mass error within 10 ppm and 0.06 Da, respectively. Peptides were matched to spectra at a false discovery rate (FDR) of 1% (strict) and 5% (relaxed) against the decoy database and quantitated using TMT6-plex method. Search result was exported and further processed for differential analysis using an in-house R-based script that was built upon the *limma* package from Bioconductor. Proteins with differential expression were identified by comparing the treatment with the control with a log_2_ fold change (log_2_ FC) cutoffs of 1 and –1 and *p* value adjusted using the Benjamini-Hochberg method of <0.05 as significant hits.

### Protein sequence analysis and predictions

RNF8 protein sequence (O76064-1) was downloaded from Uniprot and CAMKII motifs were predicted using PhosphoMotif Finder software in Human Protein Reference Database (HRRD).

RNF8 protein sequences from different species were downloaded from Uniprot and multiple sequence alignment was performed using Clustal Omega (EMBL-EBI) (https://www.ebi.ac.uk/Tools/msa/clustalo/).

### Public datasets and data analyses

Processed tumor gene expression and clinical data for TCGA (https://www.cancer.gov/tcga), REMBRANDT and Gravendeel glioma patients cohorts have been obtained from GlioVis portal (http://recur.bioinfo.cnio.es).

TCGA glioma patients RPPA level 4 data have been downloaded from TCPA portal: https://tcpaportal.org/tcpa/download.html. RNF8^low^ and RNF8^high^ glioma patients subgroups have been generated as the bottom 25% lowest RNF8 mRNA expressors and the top 25% highest mRNA RNF8 expressors in TCGA glioma tumors. For correlative analysis of RNF8 mRNA expression with protein expression levels of MRE11, CHK2, CHK2pT68, CHK1pS296, CHK1pS345, and 53BP1, reverse phase protein array (RPPA) data (level 3) for patients from TCGA glioma cohort was accessed through TCPA (v3.0) portal: https://tcpaportal.org/tcpa/download.html. R package ggpubr was used for generation of correlation plots.

One‐dimensional data‐driven grouping method was used to estimate whether the expression of gene of interest was significantly associated with cancer patient’s survival. After sorting the patients’ data by the gene expression values, the values were fitted to survival times and events using the Cox proportional hazards model; goodness-of-fit analysis was applied to get the separation between the sorted patients into low- and high-risk subgroups. The Cox hazards model and Wald test statistic were used to compute the differences between the Kaplan-Meier survival curves. Survival curves were visualized using R package survminer.

### RNA-Seq analysis

GSC TS543 were transduced with or RNF8 overexpression lentivirus. Total mRNA samples were sent to Axil Scientific (Axil Scientific Pte Ltd, Singapore) for RNA-Seq analysis. Transcriptomic sequencing (RNA-Seq) was performed on the Illumina HiSeq platform according to the standard paired-end protocol. RNA-seq data quality was monitored via FASTQC package (https://www.bioinformatics.babraham.ac.uk/projects/fastqc/). Adapters and overrepresented sequences have been removed using cutadapt software (https://cutadapt.readthedocs.io/en/stable/). Further reads preprocessing was performed by Trimmomatic (version 0.39) with the parameters: LEADING:3 TRAILING:3 SLIDINGWINDOW:4:15 MINLEN:50. Mapping of RNA-seq reads was done using STAR_2.5.0a with default parameters for RNA-seq data; RSEM software were used to quantify the gene-level expression. Deseq2 (http://www.bioconductor.org/packages/release/bioc/html/DESeq2.html) package was utilized for differential gene expression analysis. Gene set/pathway enrichment analysis was performed using the ConsensusPathDB (http://consensuspathdb.org/).

### Connectivity map analysis

To identify candidate upstream regulators of RNF8 expression we used L1000CDS2 drug screening database (https://maayanlab.cloud/L1000CDS2/#/index). We assumed that gene expression signature reflecting correlation pattern of a given gene could be used to shortlist candidate drugs mimicking the gene expression pattern in drug perturbation experiments. Gene symbols for top 150 genes either upregulated or downregulated upon RNF8 overexpression (Supplementary Table S3) were used as “up-regulated” and “down-regulated” input genes lists in the L1000CDS2 search engine, respectively. The L1000CDS2 calculates the pair-wise cosine distance between the directions of the disease-drug characteristics and provides ranked lists of scores for the candidate compounds. First, the search engine prioritized small-molecules that were predicted to mimic expression pattern of the RNF8 overexpression signature. Then, we calculated the aggregated score of each compound which took into account the compound gene pattern consistency in multiple cell lines: i) average score value for original L1000CDS2 cell line scores for each compound hit was calculated; ii) average score value for each drug hit was multiplied by number of independent cell line hits to obtain the aggregated score for each compound.

### Sucrose density gradient sedimentation analysis

After 48-h post-transfection, cells were harvested and resuspended in ice-cold 1× PBS. Cells were cross-linked with 0.4 mM DSP (dithiobis(succinimidyl propionate)) (Thermo Scientific) for 1 h at 4 °C with rotation. The crosslinking reaction was stopped by adding Tris (pH 7.5) to a final concentration of 50 mM. The cells were centrifuged, washed once with ice-cold 50 mM Tris PBS and pelleted again. Cells were lysed in IP lysis buffer for 10 min at 4 °C with rotation. The supernatant was applied to a 12 mL 10–45% (w/w) sucrose density gradient (20 mM HEPES pH 7.4, 100 mM potassium acetate, 5 mM magnesium acetate) and centrifuged at 4 °C in a SW41 Beckman rotor for 20 h at 30,000 rpm. After ultracentrifugation, the gradients were fractionated and reverse crosslinked by boiling the fractions in SDS sample loading dye (containing DTT at final concentration of 50 mM). The co-migration of target proteins was analyzed by Western blot.

### Statistical analyses

All the quantitative data was presented as mean±standard deviations as described in the figure legends. For computing the statistical significance, Student *t*-test and One-way ANOVA were performed using Graph Pad Prism (Version 9.3.1) built-in analysis. Significance was defined as *P* < 0.05. For public datasets, used Man-Whitney test and Kaplan-Meier estimate were performed using either R3.4.1 or Cytel Studio (Version 9.0.0). Significance was defined as *P* < 0.05. For multiple testing correction Benjamini-Hochberg statistic was applied to estimate the FDR. A set of R packages (e.g, ggplot2, ggpubr and surminer) was used for plots generation.

## Supplementary information

Original Data File for Figures

Supplementary Table

Figure S1

Figure S2

Figure S3

Figure S4

Figure S5

Figure S6

Supplementary Materials

Original Data File for Supp Figures

## Data Availability

All data, supplemental data, and data in repositories are available. Raw data from RNA-Seq of RNF8 vs GFP overexpressing GSC TS543 is available on the Gene Expression Omnibus database (GSE190638). Raw data from BioID-mass spectrometry analysis can be accessed using URI: https://repository.jpostdb.org/preview/11041796556371b651ba41b (Access key: 3293).
