# Supplementary material for: CAMK2D serves as a molecular scaffold for RNF8-MAD2 complex to induce mitotic checkpoint in glioma
Source: Cell Death Differ. 2023 Jul 19;30(8):1973–87. doi: 10.1038/s41418-023-01192-3 (PMC10406836; doi:10.1038/s41418-023-01192-3)
Supplement: Supplementary file 1 — Original Data File for Figures [file 41418_2023_1192_MOESM1_ESM.pdf]

Figure 1

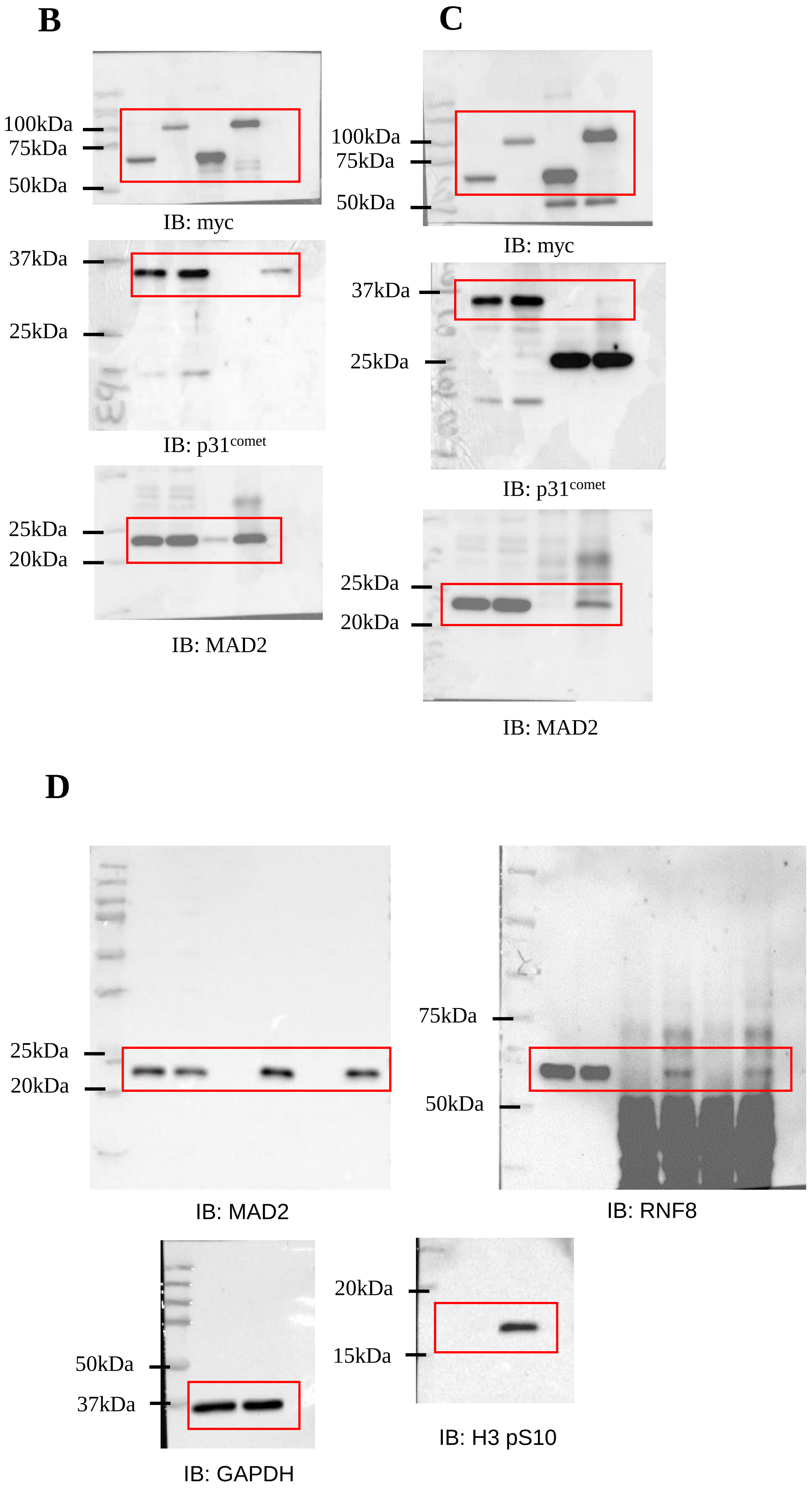

B
100kDa
75kDa
50kDa
IB: myc
37kDa
25kDa
IB: p31comet
25kDa
20kDa
IB: MAD2
C
100kDa
75kDa
50kDa
IB: myc
37kDa
25kDa
IB: p31comet
25kDa
20kDa
IB: MAD2
D
25kDa
20kDa
IB: MAD2
75kDa
50kDa
IB: RNF8
50kDa
37kDa
IB: GAPDH
20kDa
15kDa
IB: H3 pS10

Figure 1

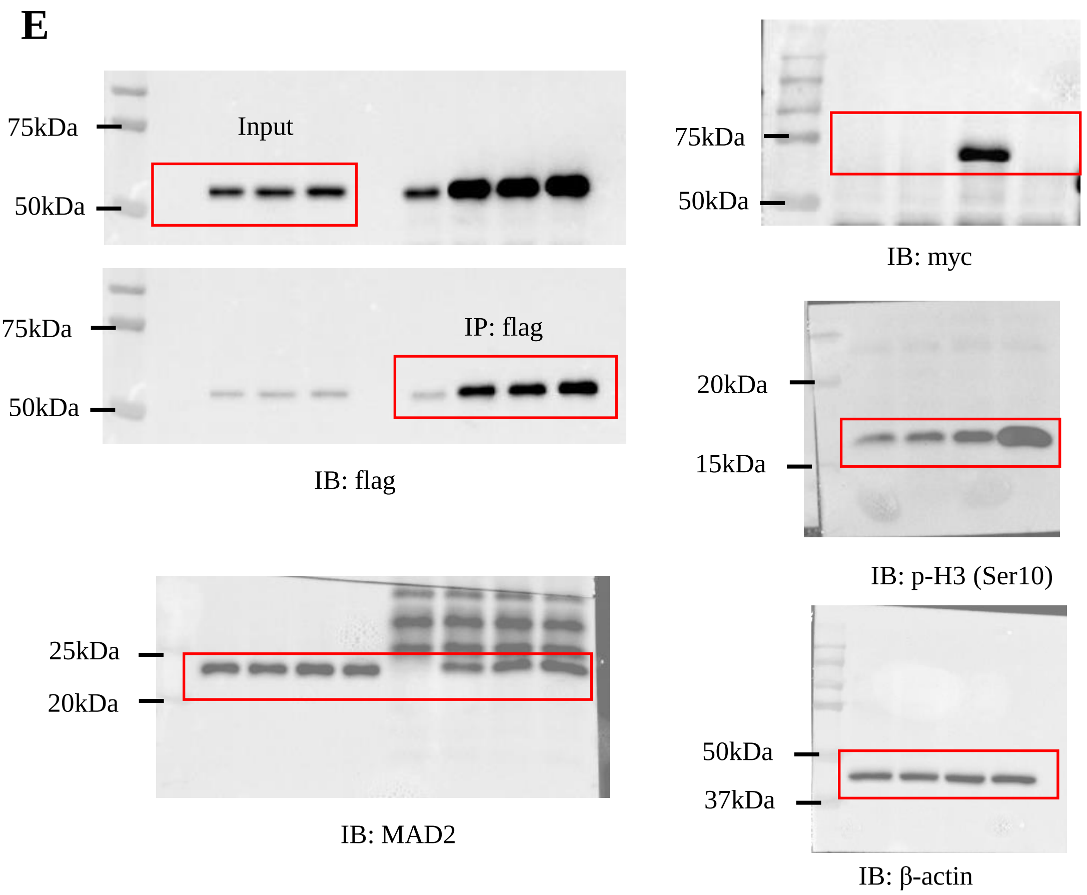
E
75kDa
50kDa
Input
75kDa
50kDa
IP: flag
IB: flag
75kDa
50kDa
IB: myc
20kDa
15kDa
IB: p-H3 (Ser10)
25kDa
20kDa
IB: MAD2
50kDa
37kDa
IB: β-actin

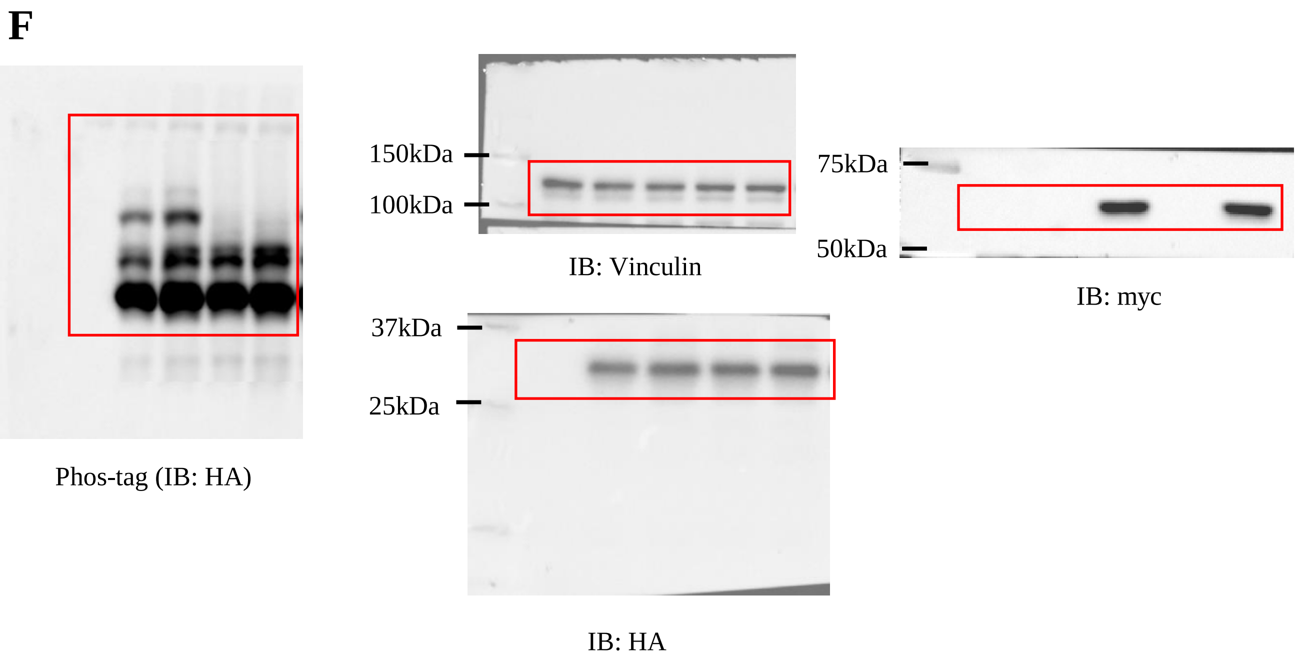
F
Phos-tag (IB: HA)
150kDa
100kDa
IB: Vinculin
75kDa
50kDa
IB: myc
37kDa
25kDa
IB: HA

Figure 1

G

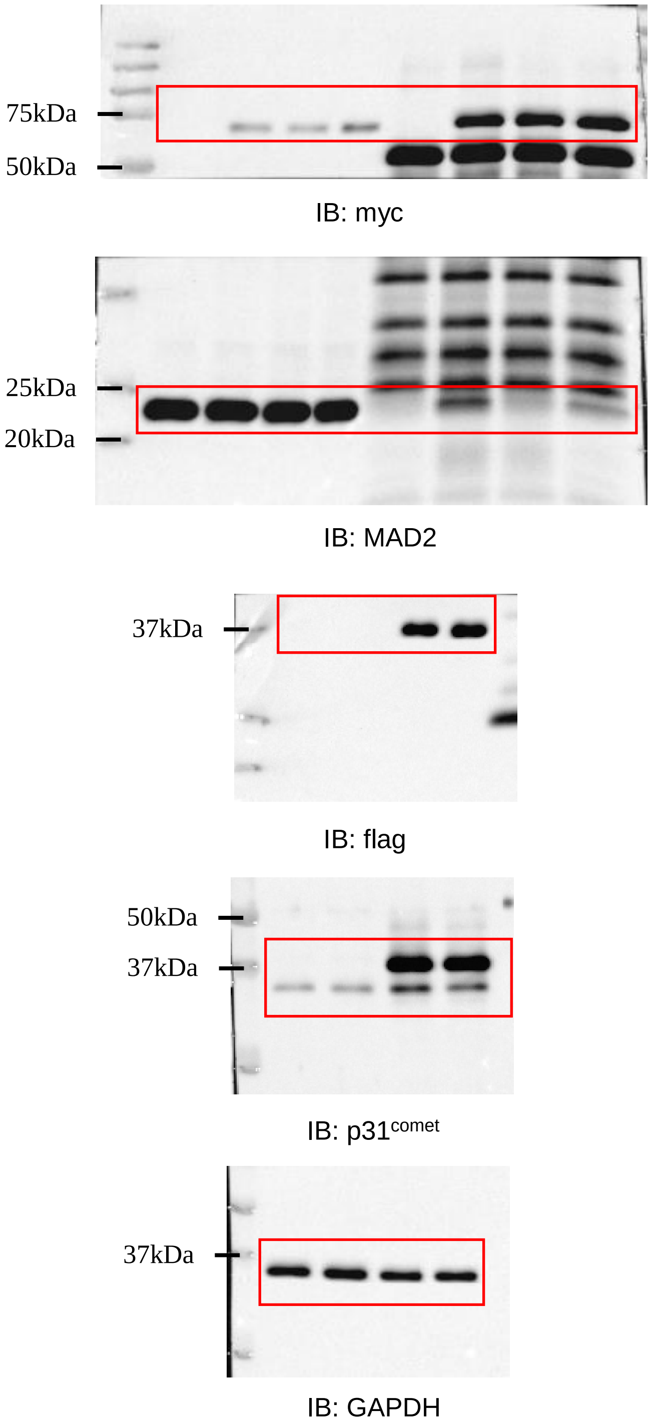

75kDa
50kDa
IB: myc
25kDa
20kDa
IB: MAD2
37kDa
IB: flag
50kDa
37kDa
IB: p31comet
37kDa
IB: GAPDH

H

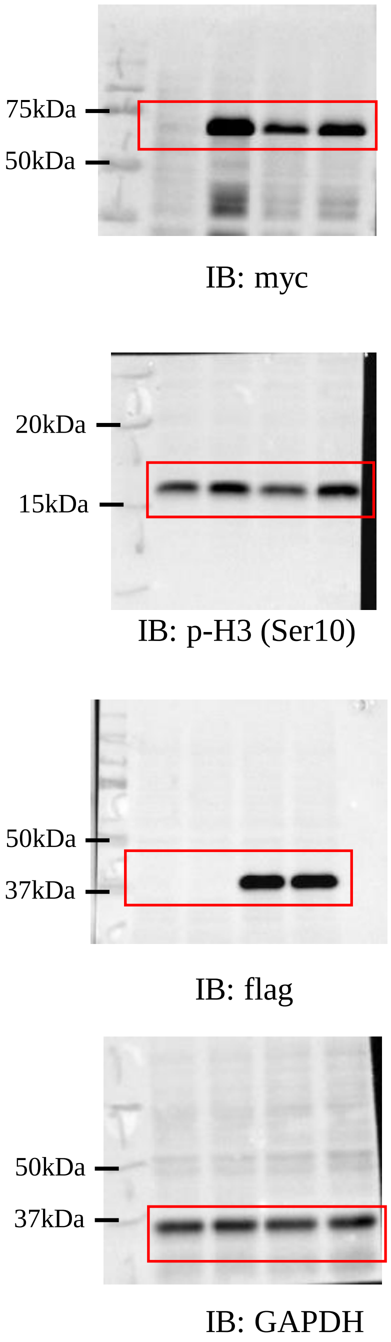

75kDa
50kDa
IB: myc
20kDa
15kDa
IB: p-H3 (Ser10)
50kDa
37kDa
IB: flag
50kDa
37kDa
IB: GAPDH

Figure 1

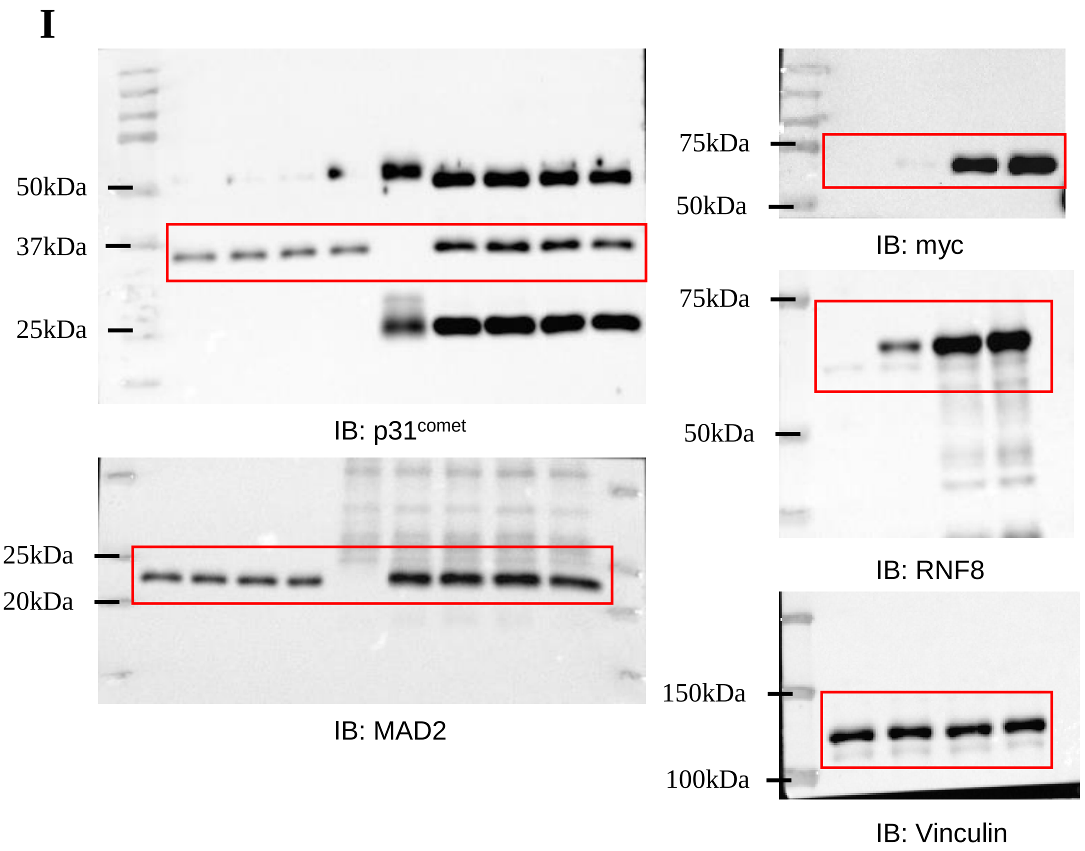
I
50kDa
37kDa
25kDa
IB: p31comet
25kDa
20kDa
IB: MAD2
75kDa
50kDa
IB: myc
75kDa
50kDa
IB: RNF8
150kDa
100kDa
IB: Vinculin

Figure 2

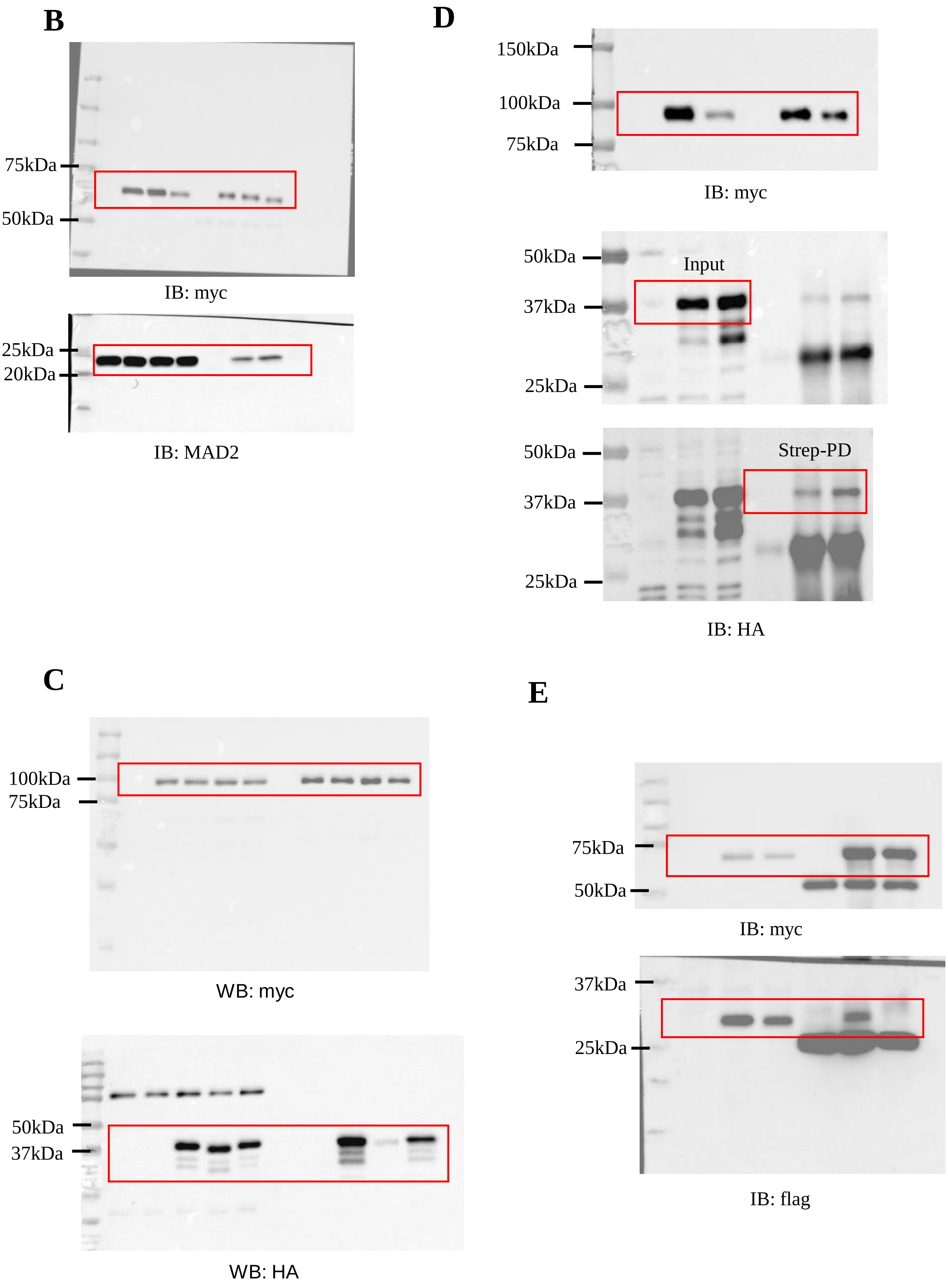
B
75kDa
50kDa
IB: myc
25kDa
20kDa
IB: MAD2
C
100kDa
75kDa
WB: myc
50kDa
37kDa
WB: HA
D
150kDa
100kDa
75kDa
IB: myc
50kDa
Input
37kDa
25kDa
50kDa
Strep-PD
37kDa
25kDa
IB: HA
E
75kDa
50kDa
IB: myc
37kDa
25kDa
IB: flag

Figure 2

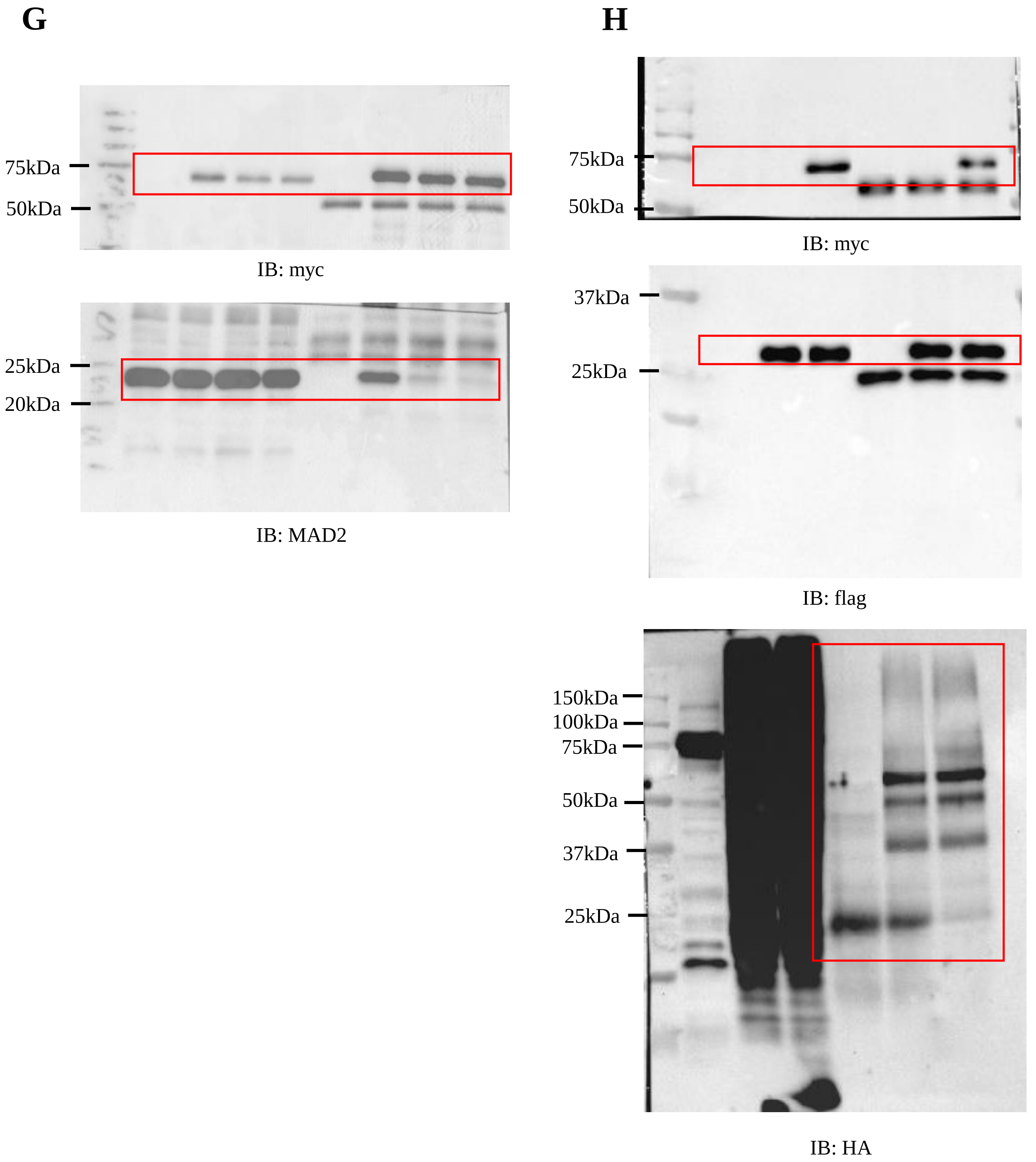
G
75kDa
50kDa
IB: myc
25kDa
20kDa
IB: MAD2
H
75kDa
50kDa
IB: myc
37kDa
25kDa
IB: flag
150kDa
100kDa
75kDa
50kDa
37kDa
25kDa
IB: HA

Figure 3

A

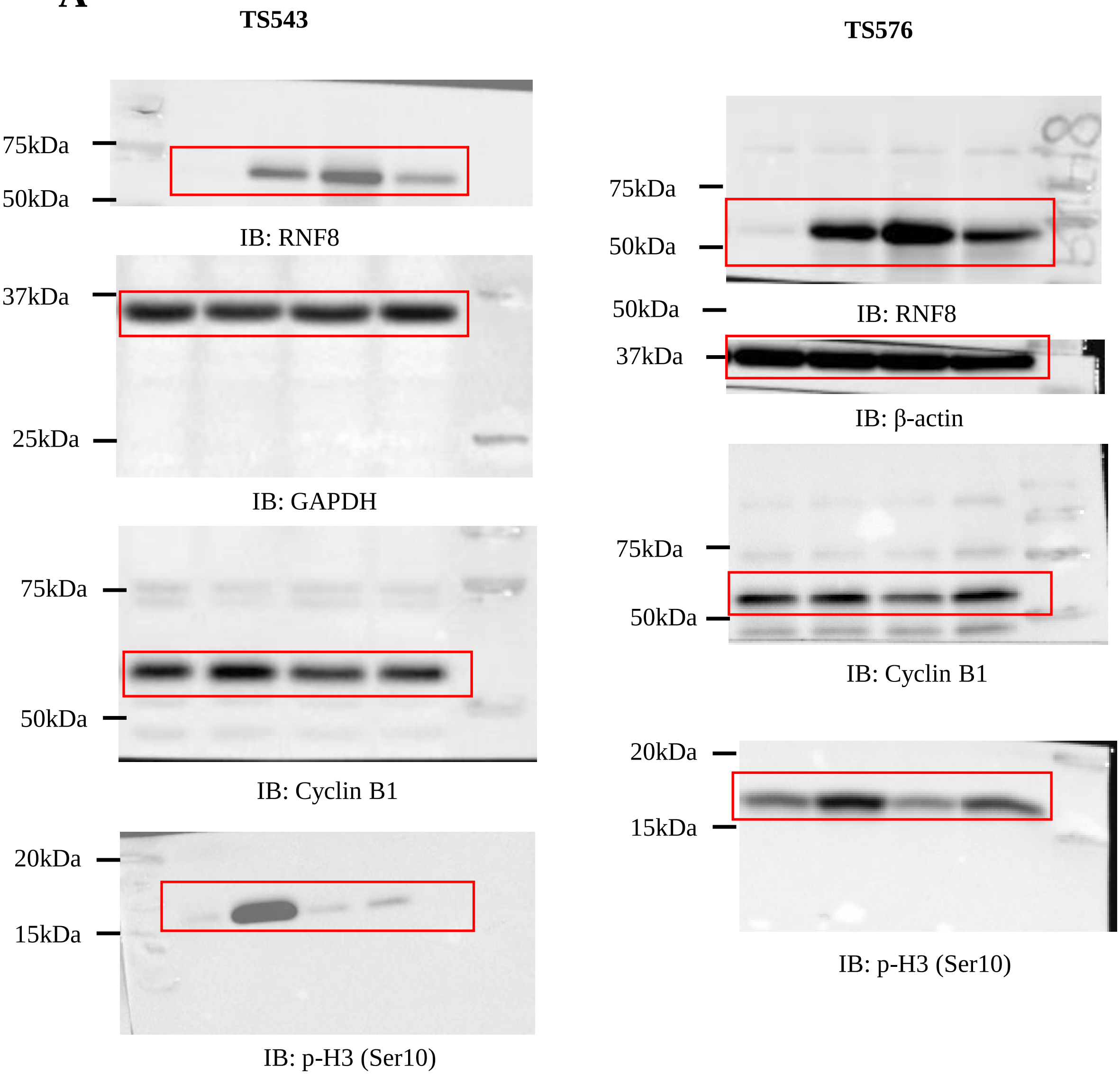
TS543
TS576
75kDa
50kDa
IB: RNF8
37kDa
25kDa
IB: GAPDH
75kDa
50kDa
IB: Cyclin B1
20kDa
15kDa
IB: p-H3 (Ser10)
75kDa
50kDa
IB: RNF8
50kDa
37kDa
IB: β-actin
75kDa
50kDa
IB: Cyclin B1
20kDa
15kDa
IB: p-H3 (Ser10)

F

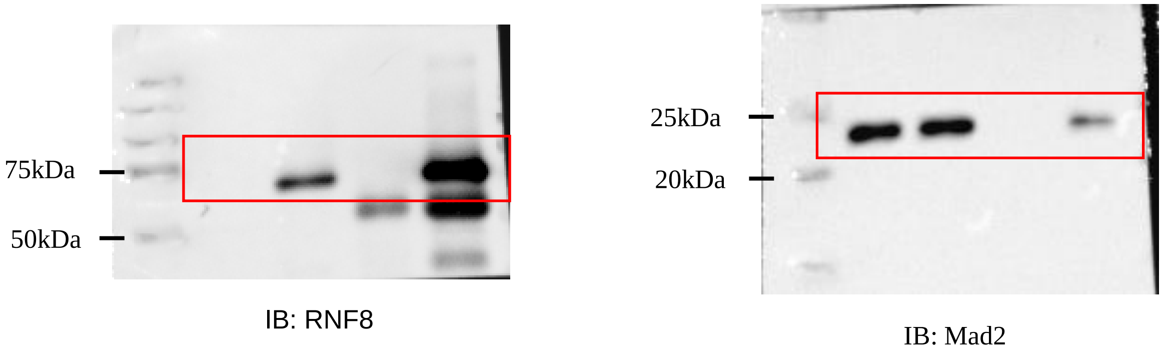
75kDa
50kDa
IB: RNF8
25kDa
20kDa
IB: Mad2

Figure 3

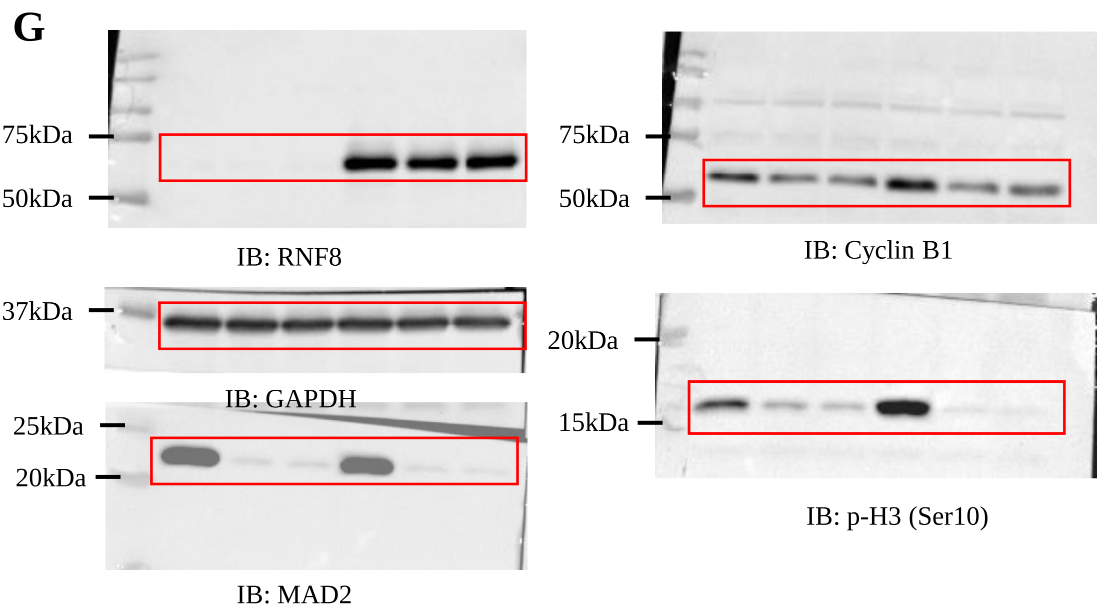
G
75kDa
50kDa
IB: RNF8
37kDa
IB: GAPDH
25kDa
20kDa
IB: MAD2
75kDa
50kDa
IB: Cyclin B1
20kDa
15kDa
IB: p-H3 (Ser10)

Figure 4

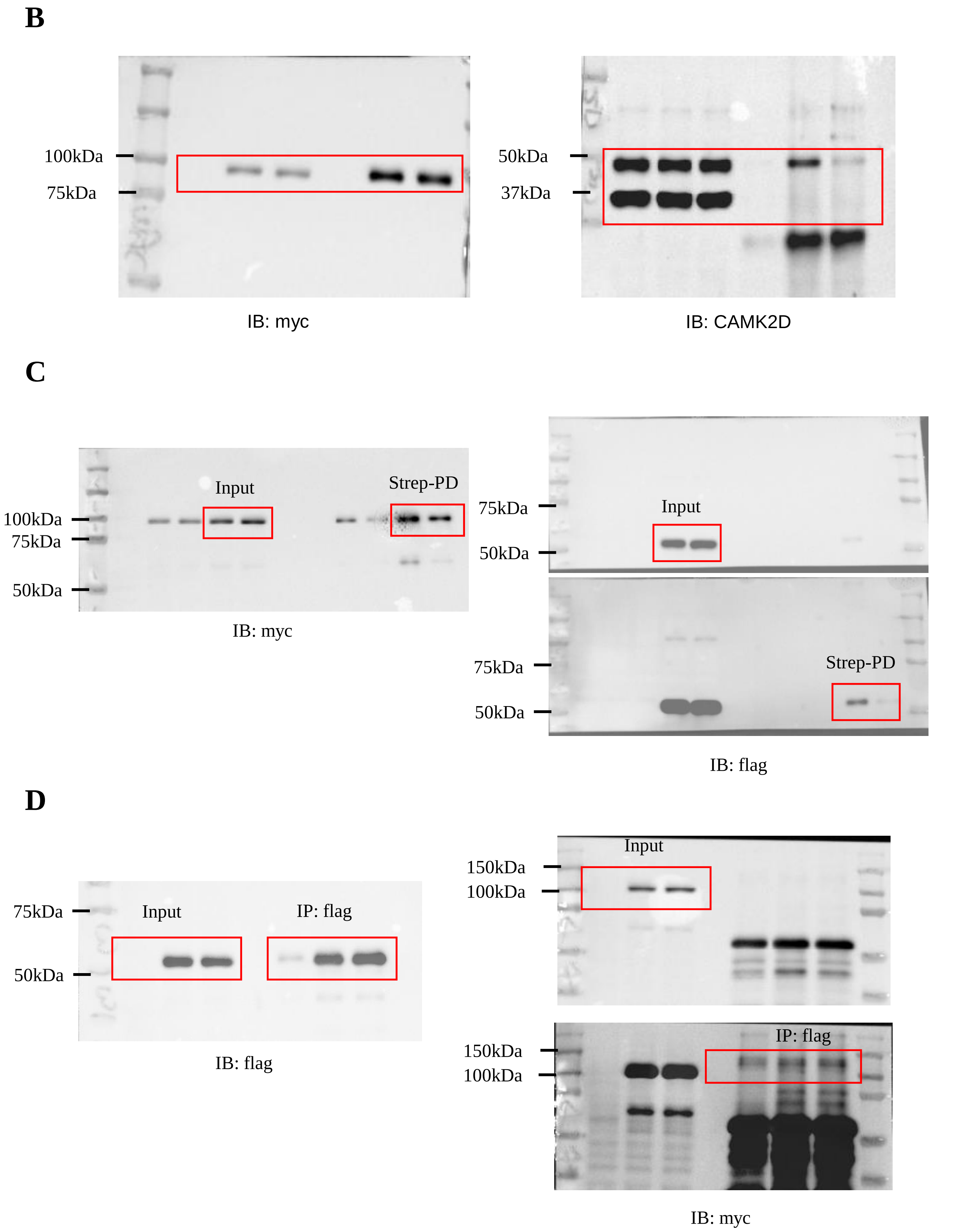
B
100kDa
75kDa
IB: myc
50kDa
37kDa
IB: CAMK2D
C
Input
Strep-PD
100kDa
75kDa
50kDa
IB: myc
75kDa
Input
50kDa
75kDa
Strep-PD
50kDa
IB: flag
D
75kDa
Input
IP: flag
50kDa
IB: flag
Input
150kDa
100kDa
IP: flag
150kDa
100kDa
IB: myc

Figure 4

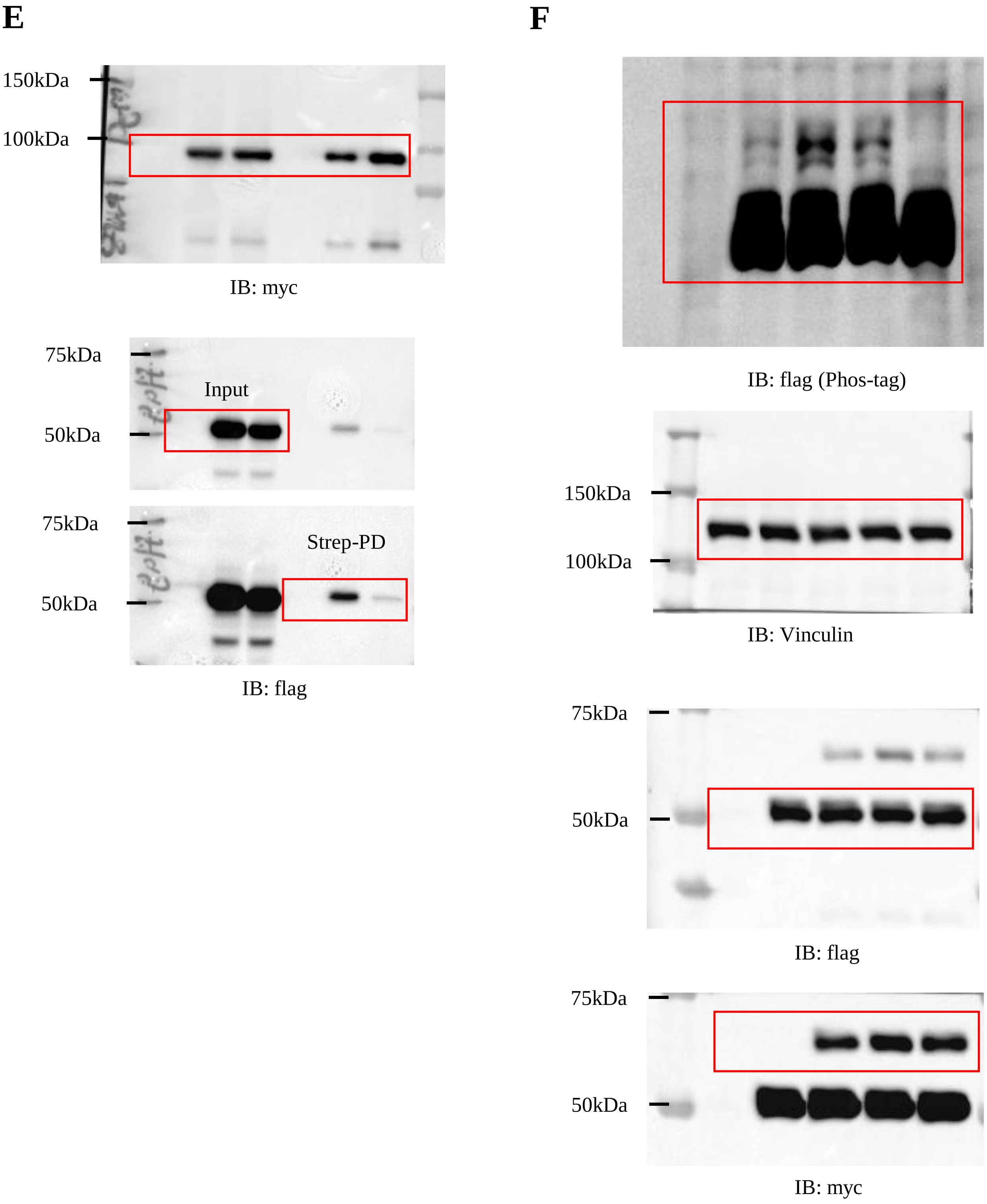
E
150kDa
100kDa
IB: myc
75kDa
Input
50kDa
75kDa
Strep-PD
50kDa
IB: flag
F
IB: flag (Phos-tag)
150kDa
100kDa
IB: Vinculin
75kDa
50kDa
IB: flag
75kDa
50kDa
IB: myc

Figure 4

G

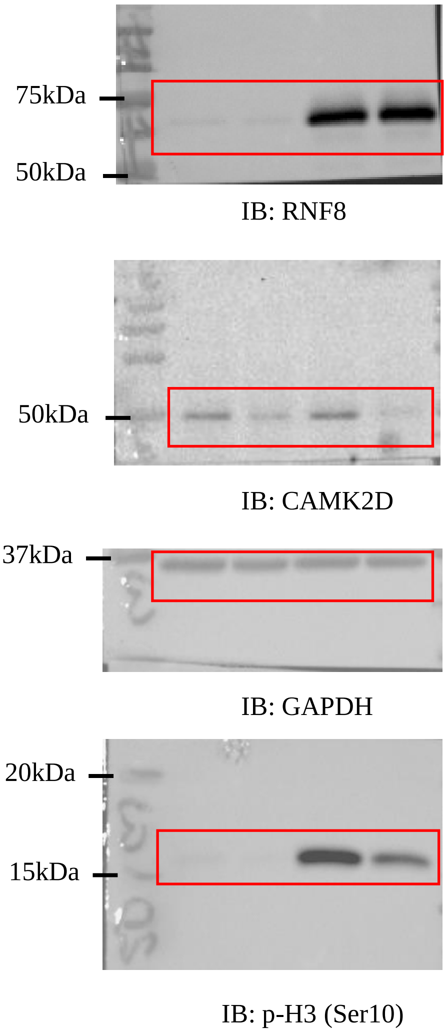

75kDa
50kDa
IB: RNF8
50kDa
IB: CAMK2D
37kDa
IB: GAPDH
20kDa
15kDa
IB: p-H3 (Ser10)

H

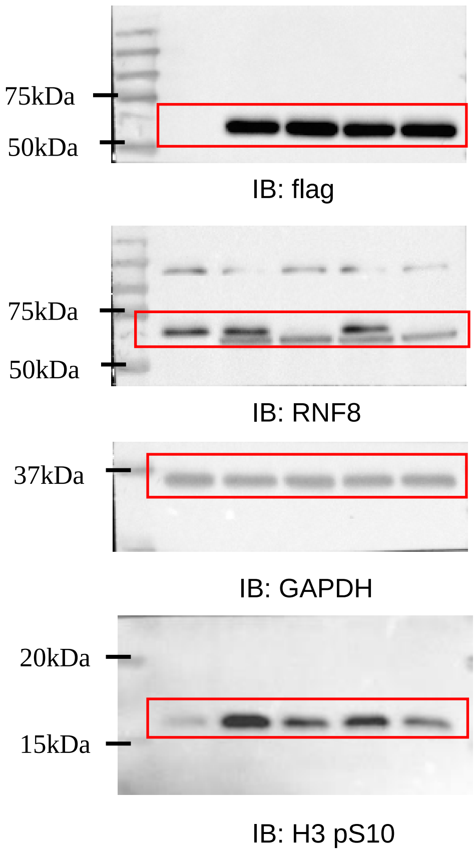

75kDa
50kDa
IB: flag
75kDa
50kDa
IB: RNF8
37kDa
IB: GAPDH
20kDa
15kDa
IB: H3 pS10

Figure 5

B

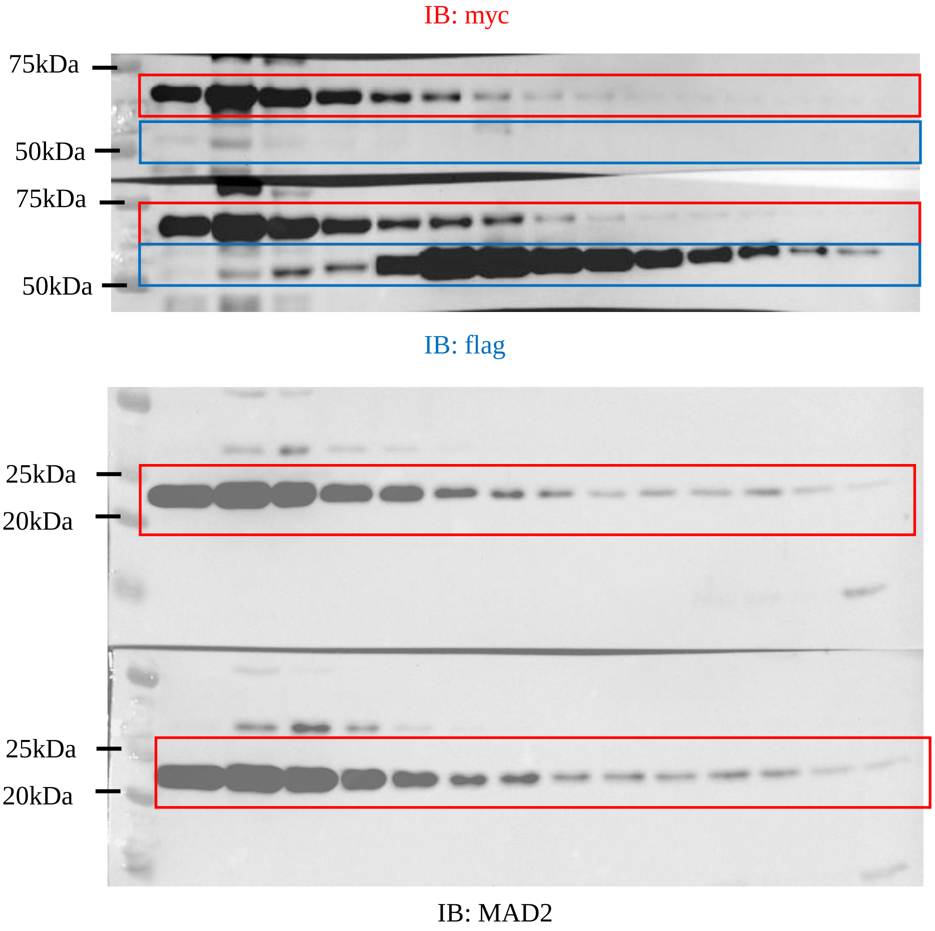
IB: myc
75kDa
50kDa
75kDa
50kDa
IB: flag
25kDa
20kDa
25kDa
20kDa
IB: MAD2

C

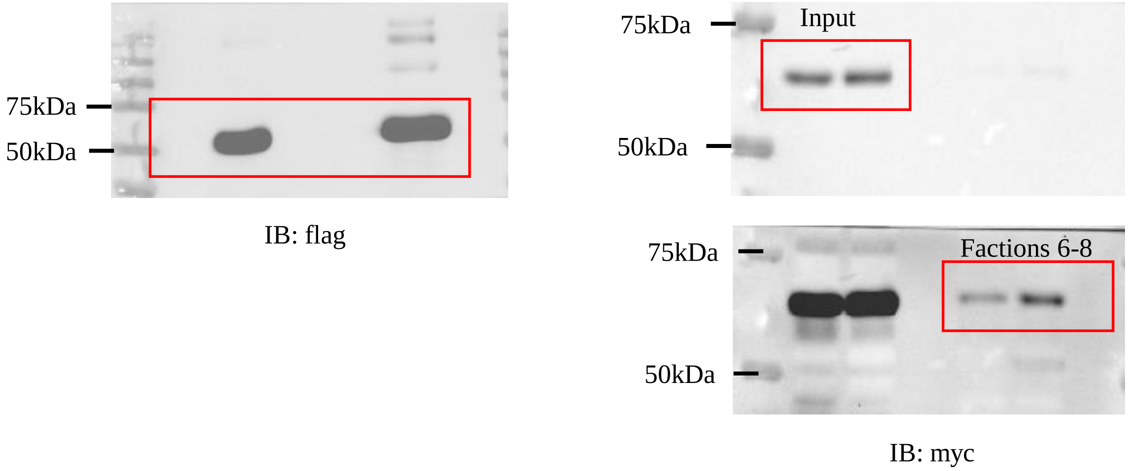
75kDa
50kDa
IB: flag
75kDa
Input
50kDa
75kDa
Factions 6-8
50kDa
IB: myc

D

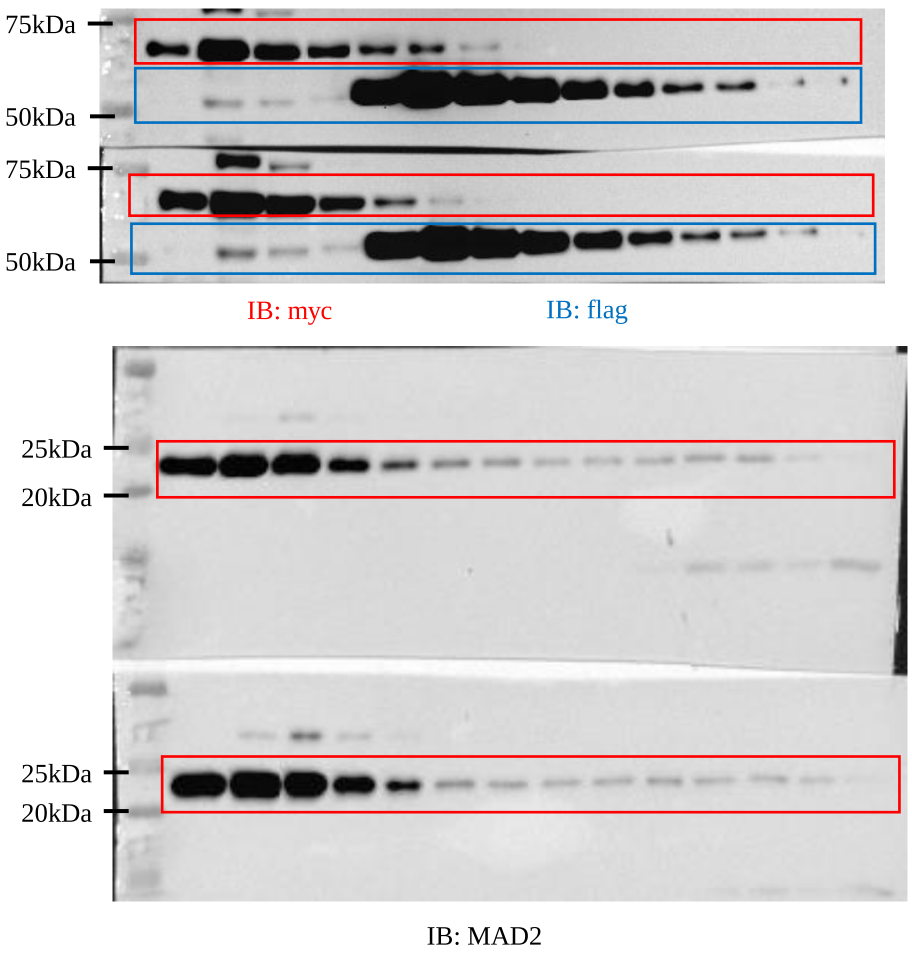
75kDa
50kDa
75kDa
50kDa
IB: myc
IB: flag
25kDa
20kDa
25kDa
20kDa
IB: MAD2

E

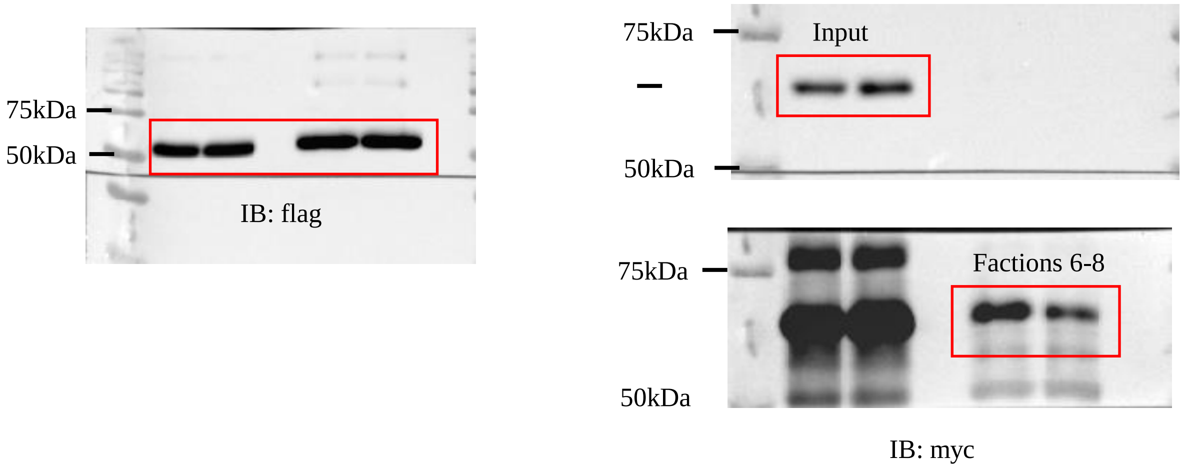
75kDa
50kDa
IB: flag
75kDa
Input
50kDa
75kDa
Factions 6-8
50kDa
IB: myc

Figure 5

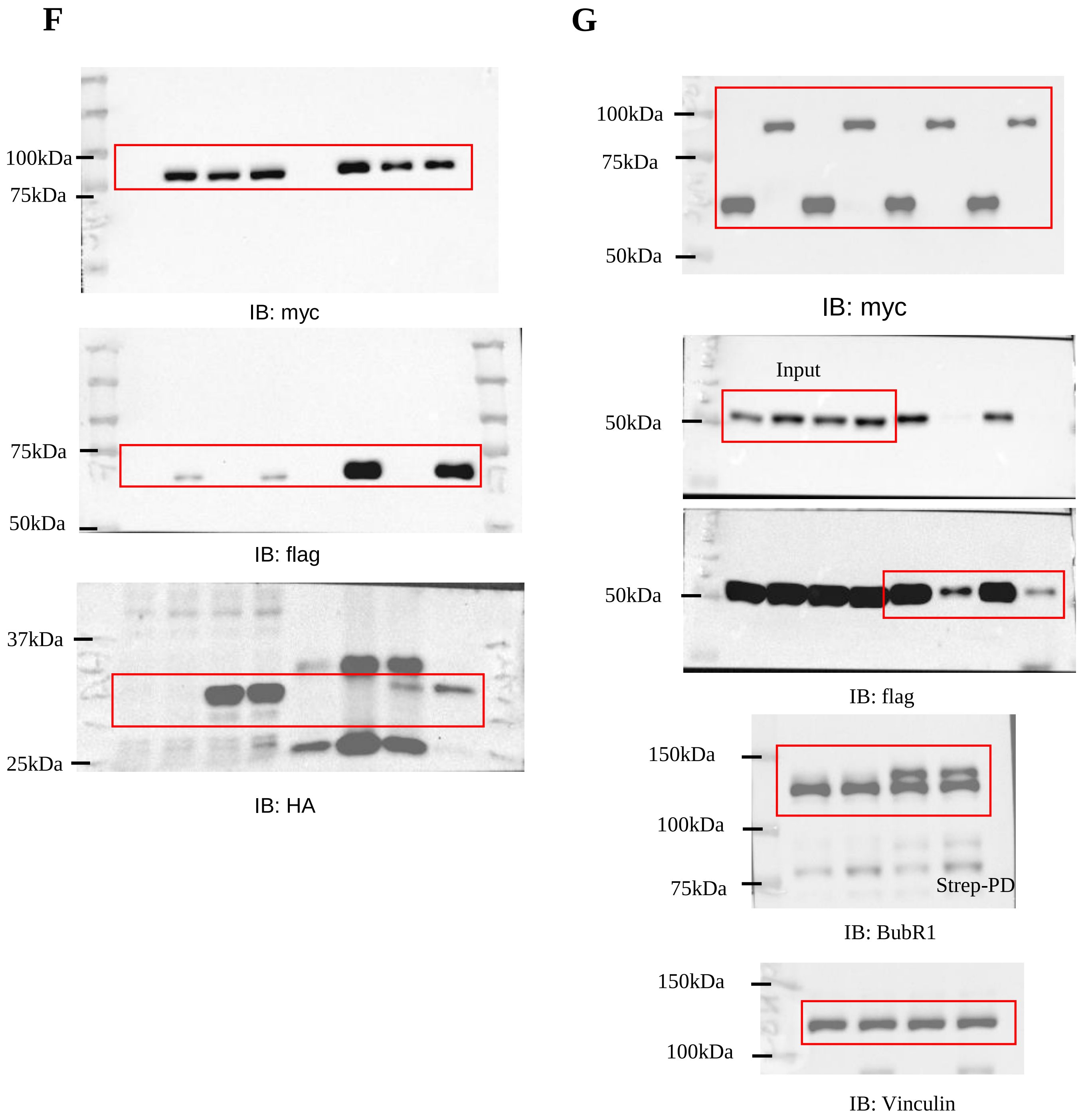
F
100kDa
75kDa
IB: myc
75kDa
50kDa
IB: flag
37kDa
25kDa
IB: HA
G
100kDa
75kDa
50kDa
IB: myc
Input
50kDa
50kDa
IB: flag
150kDa
100kDa
75kDa
Strep-PD
IB: BubR1
150kDa
100kDa
IB: Vinculin

Figure 7

E

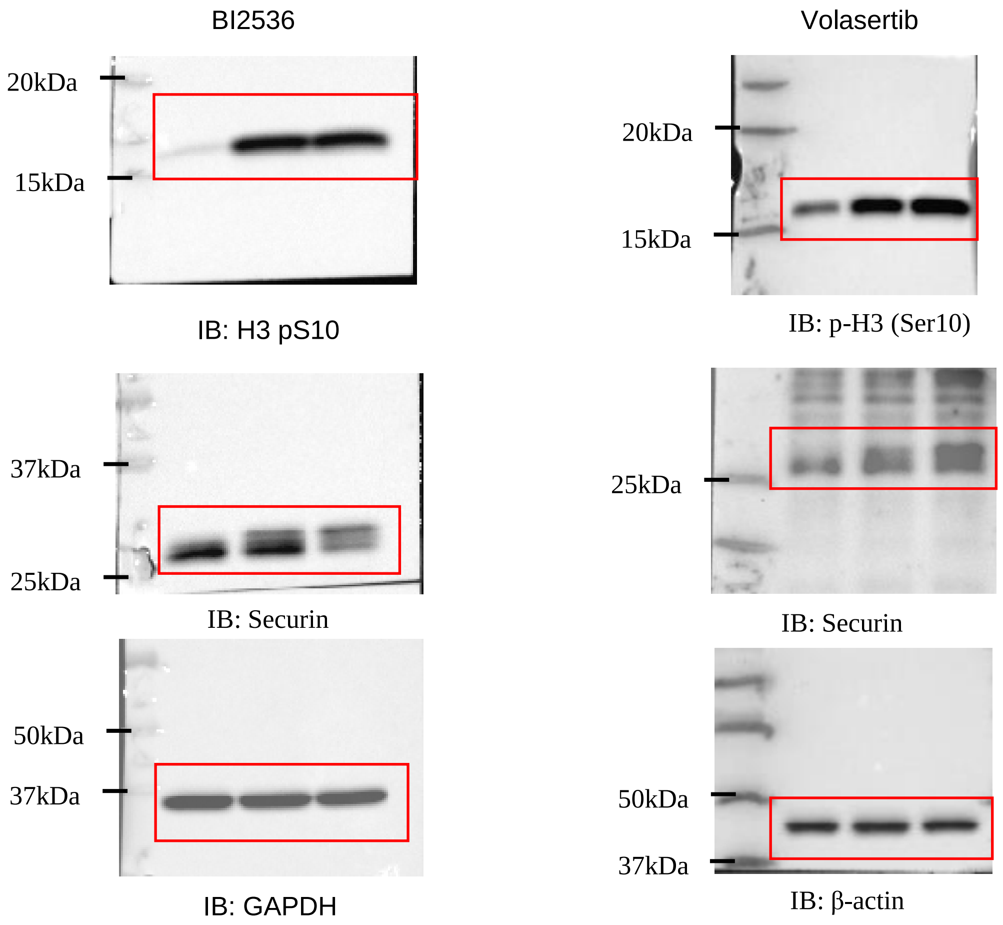
BI2536
Volasertib
20kDa
15kDa
IB: H3 pS10
20kDa
15kDa
IB: p-H3 (Ser10)
37kDa
25kDa
IB: Securin
25kDa
IB: Securin
50kDa
37kDa
IB: GAPDH
50kDa
37kDa
IB: β-actin

F

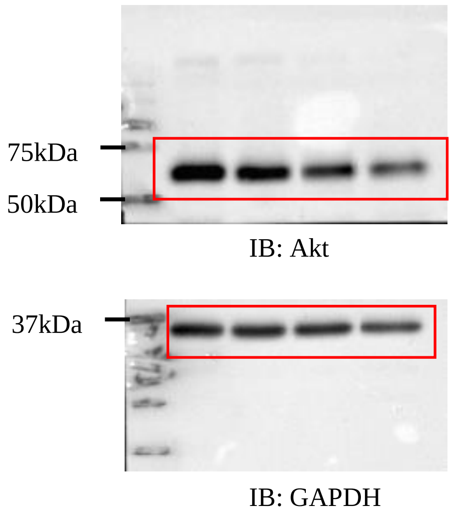
75kDa
50kDa
IB: Akt
37kDa
IB: GAPDH

Figure 7

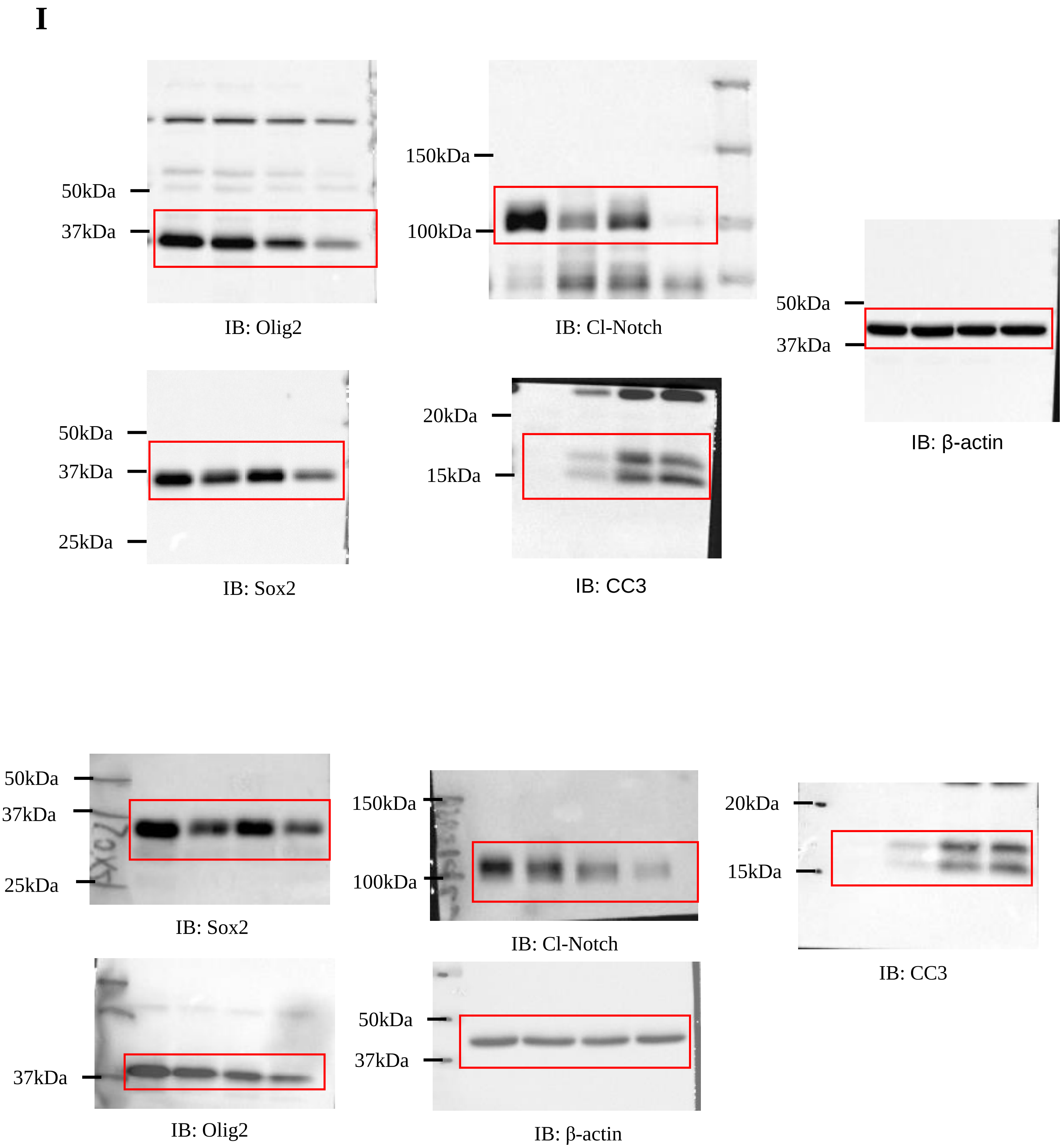
I
50kDa
37kDa
IB: Olig2
150kDa
100kDa
IB: Cl-Notch
50kDa
37kDa
IB: β-actin
50kDa
37kDa
25kDa
IB: Sox2
20kDa
15kDa
IB: CC3
50kDa
37kDa
25kDa
IB: Sox2
150kDa
100kDa
IB: Cl-Notch
20kDa
15kDa
IB: CC3
37kDa
IB: Olig2
50kDa
37kDa
IB: β-actin
