# Supplementary material for: CAMK2D serves as a molecular scaffold for RNF8-MAD2 complex to induce mitotic checkpoint in glioma
Source: Cell Death Differ. 2023 Jul 19;30(8):1973–87. doi: 10.1038/s41418-023-01192-3 (PMC10406836; doi:10.1038/s41418-023-01192-3)
Supplement: Supplementary file 2 — Supplementary Table [file 41418_2023_1192_MOESM2_ESM.docx]

**SUPPLEMENTARY TABLES**

**CAMK2D serves as a molecular scaffold for RNF8-MAD2 complex to induce mitotic checkpoint in glioma**

You Heng Chuah^1,2†^, Emmy Xue Yun Tay^1,2†^, Oleg V. Grinchuk^1,2†^, Jeehyun Yoon^1,2^, Jia Feng^1^, Srinivasaraghavan Kannan^3^, Matius Robert ^1,4^, Rekha Jakhar^1,4^, Yajing Liang^1^, Bernice Woon Li Lee^1,2^, Loo Chien Wang^5^, Yan Ting Lim^5^, Tianyun Zhao^5^, Radoslaw M Sobota^5^, Guang Lu^6^, Boon Chuan Low^7,8,9^, Karen Carmelina Crasta^1,2,4,10^, Chandra Shekhar Verma^3,8,11^, Zhewang Lin^8^, Derrick Sek Tong Ong^1,2,10,12^*

**Supplementary Table S1. Proteins which are enriched in BirA*-RNF8 vs -GFP interactomes.**

| **Gene name** | **Log_2_FC** | **Adjusted P-value** | **GFP_**  **rep#1** | **GFP_**  **rep#2** | **RNF8_**  **rep#1** | **RNF8_**  **rep#2** |
| --- | --- | --- | --- | --- | --- | --- |
| TRIM69 | 4.640713 | 0.002113 | 24.5 | 23.7 | 343.1 | 392.4 |
| RNF8 | 4.489807 | 6.54E-04 | 15734.5 | 18164.3 | 233244.7 | 222572.6 |
| CASK | 4.462742 | 0.003706 | 73.4 | 98.3 | 1511 | 825.4 |
| GOLGA7 | 4.017946 | 0.005211 | 55 | 69.8 | 311.3 | 1025.1 |
| NOTUM | 3.911187 | 0.004724 | 54.2 | 72 | 306.8 | 930.1 |
| ATIC | 3.526504 | 0.002113 | 145.8 | 155 | 973.3 | 1113.3 |
| MAD2L1BP | 3.027001 | 0.002538 | 25 | 33.2 | 124.2 | 163.8 |
| AMZ2 | 2.956114 | 0.002113 | 196 | 251.5 | 947.4 | 1131.8 |
| MAD2L1 | 2.301012 | 0.002113 | 2249 | 2459.5 | 6460.8 | 7464.8 |
| ATP5IF1 | 2.26557 | 0.003928 | 71.7 | 107.7 | 256.6 | 256.1 |
| HEMGN | 2.244432 | 0.003613 | 93.3 | 136.1 | 320 | 326.1 |
| BECN1 | 2.225646 | 0.01668 | 162.5 | 190.4 | 255.8 | 836.4 |
| ALB | 2.211604 | 0.021763 | 1461.4 | 1272.8 | 2180.9 | 6102.5 |
| C19orf48 | 2.03929 | 0.02125 | 268.2 | 250.2 | 369.8 | 983.2 |
| GRIP2 | 2.0268 | 0.007062 | 218.7 | 284.4 | 737.8 | 500.6 |
| TLR2 | 2.01659 | 0.003233 | 580.4 | 604.5 | 1289.5 | 1594.8 |
| IBTK | 1.994081 | 0.003783 | 58.5 | 60.4 | 129.1 | 157.1 |
| CENPI | 1.96605 | 0.008032 | 58.1 | 59.5 | 102.3 | 182.2 |
| RBM28 | 1.941769 | 0.008839 | 122.1 | 235.6 | 409.1 | 388.2 |
| SSRP1 | 1.898866 | 0.004943 | 2341.5 | 2657.4 | 4291.6 | 7115 |
| HAO2 | 1.725527 | 0.002906 | 407.2 | 440.3 | 821.9 | 858.6 |
| MDN1 | 1.714619 | 0.013527 | 1336.2 | 1411.4 | 1799 | 3876.5 |
| ITGA1 | 1.671922 | 0.003894 | 143 | 197.9 | 253.5 | 403.1 |
| THRAP3 | 1.629248 | 0.003457 | 6152.4 | 7547.3 | 13011.8 | 12300.9 |
| FAM76B | 1.628241 | 0.017548 | 44.2 | 52.3 | 56.1 | 133.2 |
| CCNT2 | 1.605106 | 0.004713 | 44.9 | 45.4 | 85.1 | 80.5 |
| CTTNBP2NL | 1.596968 | 0.005712 | 399.3 | 329.6 | 616.2 | 705 |
| NUCKS1 | 1.5784 | 0.025354 | 1306.6 | 2001.6 | 3769.5 | 2208.7 |
| TCOF1 | 1.550281 | 0.003613 | 14295.8 | 17325.1 | 29233.7 | 26161 |
| KCNH5 | 1.540426 | 0.007062 | 110.1 | 203.8 | 156.5 | 413.6 |
| RNPS1 | 1.532393 | 0.00504 | 382.3 | 440.6 | 583.4 | 854 |
| EIF2D | 1.524913 | 0.003639 | 237.1 | 259.7 | 394.2 | 464.1 |
| IGFBP4 | 1.524147 | 0.04454 | 502 | 634.9 | 1497.5 | 586.7 |
| WBP11 | 1.507745 | 0.007434 | 195.5 | 265.3 | 422.6 | 360.1 |
| MDC1 | 1.47307 | 0.002538 | 2229.5 | 2497.8 | 3735.4 | 4129.5 |
| PRKG1 | 1.461604 | 0.004415 | 106.4 | 122.9 | 184.3 | 194.3 |
| SYN2 | 1.453343 | 0.002906 | 922.7 | 1117.3 | 1542.6 | 1802.1 |
| MFAP1 | 1.438091 | 0.003457 | 642.7 | 720.3 | 1108.1 | 1104.3 |
| FER1L5 | 1.425299 | 0.005116 | 122.6 | 157.9 | 183.8 | 271.5 |
| BUD31 | 1.392582 | 0.00852 | 153 | 199.5 | 302.9 | 250.9 |
| FOXK1 | 1.375383 | 0.044864 | 1018.1 | 1153.5 | 2617.7 | 1021.6 |
| MYL6 | 1.372225 | 0.002906 | 3234.1 | 2629.3 | 4955.7 | 4142.9 |
| SET | 1.366371 | 0.002906 | 1613.4 | 2013.3 | 2639.6 | 2946.7 |
| ZMAT2 | 1.366247 | 0.005216 | 1380.9 | 1692.6 | 2533.9 | 2207.9 |
| FUS | 1.360919 | 0.004808 | 99.2 | 122.2 | 156.6 | 184.2 |
| CDC40 | 1.348634 | 0.025354 | 38.5 | 44.2 | 84.7 | 45.8 |
| CTR9 | 1.347445 | 0.013775 | 2138.9 | 2549.6 | 4460.9 | 2810.5 |
| ERH | 1.34702 | 0.002906 | 2312.2 | 2573.2 | 3608.9 | 3836.4 |
| PEX19 | 1.332327 | 0.005523 | 151.5 | 212.7 | 260.3 | 285.1 |
| CXorf56 | 1.32177 | 0.003457 | 3160.1 | 3772.4 | 5157.2 | 5201.7 |
| BCLAF1 | 1.31047 | 0.003233 | 3104.2 | 3644.2 | 4971.4 | 5039.3 |
| RNF20 | 1.310213 | 0.007853 | 818.3 | 1014.8 | 1520.8 | 1207.5 |
| CFDP1 | 1.296058 | 0.007934 | 882.4 | 1113 | 1625.2 | 1311.5 |
| CASKIN2 | 1.284034 | 0.004808 | 416.7 | 518.1 | 687.4 | 673.1 |
| TRIR | 1.282442 | 0.002906 | 2263.4 | 2657.4 | 3464.9 | 3696.8 |
| PRPF38A | 1.252266 | 0.003613 | 551.2 | 621.6 | 779.5 | 895.7 |
| PNISR | 1.246779 | 0.003613 | 3590.3 | 4311.6 | 5694 | 5513.9 |
| ZBTB10 | 1.228437 | 0.003715 | 711.6 | 827.1 | 964.6 | 1200.4 |
| TBC1D9 | 1.228339 | 0.035753 | 141.9 | 191.7 | 132 | 373.6 |
| HIKESHI | 1.217736 | 0.006274 | 94.6 | 138 | 147.2 | 173.9 |
| YAP1 | 1.215989 | 0.003613 | 524 | 644.9 | 740 | 885.8 |
| GMNN | 1.198678 | 0.009699 | 35.8 | 40 | 44.5 | 60.8 |
| RIPK1 | 1.188926 | 0.005523 | 113.8 | 122.2 | 161 | 161.8 |
| ZRANB2 | 1.183802 | 0.003588 | 1003.5 | 1063.6 | 1398.8 | 1416.2 |
| TAF15 | 1.177106 | 0.005116 | 1977.7 | 2142.2 | 2434.2 | 3179.2 |
| ESF1 | 1.167794 | 0.005624 | 116.8 | 142 | 163.1 | 185 |
| SRRM1 | 1.159806 | 0.004713 | 358 | 359.2 | 472.8 | 488.3 |
| ZNF749 | 1.141353 | 0.005687 | 135.7 | 154.4 | 185.1 | 198.4 |
| SMAP | 1.129811 | 0.003613 | 1507.7 | 1776 | 2126.5 | 2171.3 |
| CIART | 1.123587 | 0.003928 | 446.6 | 579.5 | 595.2 | 742.3 |
| MICALL1 | 1.111983 | 0.007351 | 107.1 | 116.3 | 130.3 | 160.4 |
| NASP | 1.107443 | 0.002906 | 10039.1 | 11461.4 | 13070.6 | 14688.1 |
| CHTOP | 1.106026 | 0.006062 | 126.2 | 161.9 | 166.7 | 204.5 |
| RARA | 1.100193 | 0.019165 | 400.4 | 635.3 | 692.2 | 620.2 |
| PHF8 | 1.096423 | 0.012531 | 36.4 | 50.5 | 55.6 | 55.1 |
| PSMB11 | 1.094611 | 0.006256 | 107.4 | 175.6 | 144.3 | 215.2 |
| GTF2F1 | 1.066616 | 0.003613 | 1916.5 | 1951.6 | 2389.6 | 2468.5 |
| SPDL1 | 1.061911 | 0.003465 | 2991.2 | 3386 | 3804.9 | 4170.7 |
| ACIN1 | 1.060482 | 0.005116 | 657.7 | 782.2 | 906.7 | 889.6 |
| TPM1 | 1.059997 | 0.004782 | 1127.9 | 1059.7 | 1344.5 | 1390 |
| PHAX | 1.050627 | 0.004415 | 630 | 720.2 | 776.7 | 900 |
| HERC2 | 1.050184 | 0.005395 | 329 | 390.4 | 396.5 | 497 |
| CEMIP2 | 1.045374 | 0.006085 | 199.5 | 257.7 | 261.4 | 302.4 |
| BCAS2 | 1.044926 | 0.008032 | 322.1 | 437 | 466.9 | 466.6 |
| HDGFL2 | 1.0448 | 0.003613 | 2228.7 | 2546.8 | 2880.3 | 3017.8 |
| CWC15 | 1.036106 | 0.003928 | 1194.6 | 1343.3 | 1442.8 | 1679.4 |
| C19orf25 | 1.026025 | 0.012059 | 59 | 68.2 | 64.7 | 91.9 |
| IK | 1.013927 | 0.003894 | 1428 | 1580.2 | 1707.9 | 1935.2 |
| TPM3 | 1.011597 | 0.003588 | 3051.9 | 3229 | 3815.2 | 3775.5 |
| CCNA2 | 1.011189 | 0.004563 | 861.3 | 952.6 | 1111.5 | 1079 |
| MNAT1 | 1.008728 | 0.007061 | 137.9 | 136.6 | 165.9 | 165.5 |
| OLA1 | 1.00612 | 0.008231 | 66.6 | 76.2 | 84.3 | 87.6 |
| SNRPA1 | 1.001581 | 0.009867 | 3797.8 | 3459.6 | 3912.2 | 4827.5 |

**Supplementary Table S2. Proteins which are depleted in BirA*-*FHA vs -WT interactomes.**

| **Gene name** | **Log_2_FC** | **Adjusted P-value** | **WT_**  **rep#1** | **WT_**  **rep#2** | ***FHA_**  **rep#1** | ***FHA_**  **rep#2** |
| --- | --- | --- | --- | --- | --- | --- |
| CAMK2D | -1.66882 | 0.028581 | 2457.9 | 2956.4 | 1018.9 | 761.7 |
| U2AF2 | -1.26309 | 0.028581 | 1574.7 | 1954.4 | 863.9 | 668.3 |
| ERH | -1.25589 | 0.028581 | 9105.8 | 10458.3 | 4584.9 | 3941.9 |
| BCLAF1 | -1.20834 | 0.028581 | 4363.5 | 5305.5 | 2520.3 | 1860.5 |
| THRAP3 | -1.16344 | 0.028581 | 9375.4 | 11252 | 5517.5 | 4123.4 |
| TCOF1 | -1.00224 | 0.028581 | 35637.5 | 42181.9 | 22971.9 | 17651.1 |

**Supplementary Table S3. The RNF8 overexpression query signature used for CMA.**

| **Upregulated genes** | | |
| --- | --- | --- |
| RNF8 | SERPINE1 | WDR62 |
| VGF | UNC13A | RRM2 |
| EEF1A2 | HIST1H2BH | C12orf75 |
| HIST1H2BG | PCDH8 | ALYREF |
| MDK | NGFR | CAMKV |
| HIST1H1B | NOTCH3 | STMN3 |
| TNC | DLG3 | DCT |
| CRABP2 | CCNA1 | CCNA2 |
| HIST1H2BJ | SYT7 | SNRPG |
| TMSB10 | TUBB3 | NID1 |
| HIST1H2AB | S100A4 | IDI1 |
| IFI30 | MYBL2 | FAM83D |
| RNASE1 | THSD1 | NBL1 |
| TNFAIP2 | DNAAF3 | PLK4 |
| HBA1 | HIST2H2AA3 | CCDC74A |
| EPPK1 | TUBA4A | RFC3 |
| ELAVL3 | TK1 | BIRC5 |
| CDKN1A | ITM2A | CDH24 |
| HIST1H2AH | CXCR4 | APOBEC3B |
| CNN2 | ANXA1 | CTSV |
| MT2A | COX7B | MAP1A |
| DNAH3 | HIST1H2BF | TXN |
| CD24 | TUBB6 | ANXA2 |
| HIST1H2AE | BAIAP3 | MKI67 |
| CRISPLD2 | CAMSAP3 | EMP2 |
| SLC30A3 | PCDH19 | NEMP1 |
| HIST1H4B | SDC1 | GINS2 |
| HIST2H3D | NPTXR | PCLAF |
| ASF1B | MCM4 | SMC4 |
| COL1A1 | PODXL | ANLN |
| SEPTIN3 | SCRT1 | HIST2H4A |
| HIST1H2AG | HIST1H4E | DBI |
| HIST4H4 | HSPE1 | PBK |
| HIST2H2BF | CCNB1 | H2AFX |
| CACNA1G | C1QL4 | ESCO2 |
| LIPG | ASB2 | KIF20A |
| JPH4 | HIST1H2BO | TUBA1C |
| KCNH2 | ACTL8 | TUBA1B |
| HIST2H3C | CKS2 | NCAPG2 |
| TMSB4X | UBL5 | B2M |
| HIST1H4H | HIST2H2AA4 | PPIA |
| DRAXIN | GNG4 | HMGB1 |
| SNHG10 | CTSA | CDC45 |
| CCN3 | DNMT3B | HIST1H4C |
| GCH1 | TM4SF1 | FZD7 |
| HIST1H1D | TUBB4B | C2orf78 |
| ACAT2 | DPYSL3 | INSIG1 |
| ATP1A3 | DCX | TAGLN2 |
| HIST1H2BD | MGP | NUSAP1 |
| EPHB2 | ITPR3 | PCNA |
| **Downregulated genes** | | |
| IGBP1 | PTPRJ | RPL3 |
| EPB41L4A-AS1 | ADGRL3 | APOC1 |
| LIFR | EIF4B | RHBDD1 |
| ARHGAP35 | GNG7 | PYCR1 |
| ARNT2 | KLF4 | BEST1 |
| CCDC50 | HSPA9 | GPR37L1 |
| MAPK8IP3 | PHKA1 | UBL3 |
| ZKSCAN1 | GAPDH | EXTL1 |
| NQO1 | ASS1 | COL11A2 |
| OGT | CTNNA3 | PRPSAP1 |
| SPTBN1 | CMTM5 | ITGA10 |
| MRPS18A | PRRX1 | CRTAP |
| UST | GPC2 | GATB |
| SEMA5A | CCNB1IP1 | DIPK1C |
| AP2A2 | IARS | OPHN1 |
| SOCS2-AS1 | ARHGAP20 | FIBIN |
| GRAMD1A | MOB3B | PCMTD2 |
| CLIP2 | COL4A5 | ZNF853 |
| EIF1 | ZNF37BP | PGLS |
| SOD3 | STK10 | BCAT1 |
| CIRBP | LSAMP | PKD1P5 |
| LPGAT1 | RNF187 | METRN |
| CDH19 | C16orf58 | SNHG8 |
| PCDHGC3 | ZCCHC24 | EPAS1 |
| AKT3 | EIF3A | LONP1 |
| H19 | KLF2 | MGLL |
| H6PD | XPOT | HIPK2 |
| SLC25A6 | EEF2 | CHL1 |
| SHC3 | SLC44A1 | APOE |
| NEU4 | DLGAP4 | CNDP1 |
| VIM | TCEA1 | AATK |
| PLEKHG1 | RACK1 | COL15A1 |
| ADGRB1 | GRID2 | ZFAS1 |
| ARHGAP31 | NCAM1 | PPP2R2B |
| MACROD1 | PTPRJ | RCAN1 |
| MARVELD1 | ADGRL3 | COL11A1 |
| PLD3 | EIF4B | ALDH2 |
| NOP53 | GNG7 | CNDP2 |
| CHPF | KLF4 | ERBB3 |
| ATP1A2 | HSPA9 | SNHG32 |
| GAS5 | PHKA1 | ANK2 |
| WSB1 | GAPDH | TRIM2 |
| CDC42BPA | ASS1 | SHMT2 |
| EHD3 | CTNNA3 | OBSL1 |
| YARS | CMTM5 | SH3D19 |
| RBMS3 | PRRX1 | COL20A1 |
| TKT | GPC2 | NIBAN1 |
| KIAA1755 | CCNB1IP1 | FKBP9 |
| GTPBP2 | IARS | ASTN2 |
| GPRC5B | ARHGAP20 | AZGP1 |

**Supplementary Table S4. Primers used for cloning.**

| **Primer names** | **Primer sequences (5’🡪 3’)** |
| --- | --- |
| myc-RNF8-EcoRI-F | AAAGAATTCATGGGGGAGCCCGGCTTCTTCG |
| myc-RNF8-XhoI-R | AAACTCGAGGAACAATCTCTTTGCTTTTCG |
| BirA*-RNF8-XhoI-F | AAACTCGAGGGGGAGCCCGGCTTCTTC |
| BirA*-RNF8-EcoRI-R | AAAGAATTCTCAGAACAATCTCTTTGCTTTTCG |
| flag-MAD2-EcoRI-F | AAAAGAATTCATGGCGCTGCAGCTCT |
| flag-MAD2-HindIII-R | AAAAAAGCTTGTCATTGACAGGAATTTTGTAGGCC |
| flag-CDC20-EcoRI-F | AAAGAATTCATGGCACAGTTCGCGTTCGAGAGTG |
| flag-CDC20-HindIII-R | AAAAAGCTTTCAGCGGATGCCTTGGTGGATGAGG |
| flag-p31-EcoRI-F | AAAGAATTCATGGCGGCGCCGGAGG |
| flag-p31-HindIII-R | AAAAAGCTTTCACTCGCGGAAGCCTTTAAATGTCACTG |
| p31-Q83A-F | AGCATATCATGTATGCACGCCAGCAGCTCC |
| p31-Q83A-R | GGAGCTGCTGGCGTGCATACATGATATGCT |
| p31-F191A-F | CTTGTTTGCGCCGTCTCGCCCGAGCCATATTCATGG |
| p31-F191A-R | CCATGAATATGGCTCGGGCGAGACGGCGCAAACAAG |
| HA-p31-HindIII-F | AAAAAGCTTATGGCGGCGCCGGAGG |
| HA-p31-XbaI-R | AAATCTAGACTCGCGGAAGCCTTTAAATGTCACTGG |
| p31-S102A-F | CTTTTACCGAAAACCTGCTCCCCAGGCAGAGG |
| p31-S102A-R | CCTCTGCCTGGGGAGCAGGTTTTCGGTAAAAG |
| flag-CAMK2D-EcoRI-F | AAAGAATTCATGGCTTCGACCACAACCTGCAC |
| flag-CAMK2D-XhoI-R | AAACTCGAGTTAGATGTTTTGCCACAAAGAGGTGCC |
| CAMK2D-T287A-F | TGATGCACAGACAGGAGGCTGTAGACTGCTTGAAG |
| CAMK2D-T287A-R | CTTCAAGCAGTCTACAGCCTCCTGTCTGTGCATCA |
| CAMK2D-K43R-F | TGGACAAGAATATGCTGCCAGAATTATCAACACCAAAAAGCT |
| CAMK2D-K43R-R | AGCTTTTTGGTGTTGATAATTCTGGCAGCATATTCTTGTCCAG |
| CAMK2D-D136N-F | ATGGCATAGTTCACAGGAACCTGAAGCCTGAGAAT |
| CAMK2D-D136N-R | ATTCTCAGGCTTCAGGTTCCTGTGAACTATGCCAT |
| RNF8-S157A-F | AATAAGGAATTGAGAACTAAAAGGAAATTCGCTTTGGATGAATTA GCAGGTC |
| RNF8-S157A-R | GACCTGCTAATTCATCCAAAGCGAATTTCCTTTTAGTTCTCAATTCCTTATT |
| RNF8-T198A-F | AAGGTGAAGTGGCCAGTGCACCCTCTGACAATTTG |
| RNF8-T198A-R | CAAATTGTCAGAGGGTGCACTGGCCACTTCACCTT |
| RNF8-R479A-R | AAACTCGAGGAACAATCTCTTTGCTTTTGCTTCCCTAATG |
| RNF8-3A-F | GAATTGTTCTCATTGCGGCT**GCA**AAAGCAAAGAG |
| RNF8-3A-R | CTCTTTGCTTTTGCAGCCGCAATGAGAACAATTC |
| RNF8-AgeI-R | AAAACCGGTATGCATATTCAGATCCTCTTCTGAGATGAGTTTTT |
| RNF8-K480R-R | AAACTCGAGGAACAATCTCTTTGCTGCTCGTTCCCTAATG |
| BirA*-GFP-EcoRI-F | AAAGAATTCATGGTGAGCAAGGGCGAGGAGC |
| BirA*-GFP-HindIII-R | AAAAAGCTTCTTGTACAGCTCGTCCATGCCGAGAG |
| HA-MAD2-HindIII-F | AAAAAGCTTATGGCGCTGCAGCTC |
| HA-MAD2-XbaI-R | AAATCTAGATCAGTCATTGACAGGAATTTTGTAGG |
| RNF8-Q5-S157A-F | AAGGAAATTCGCTTTGGATGAATTAGCAGGTC |
| RNF8-Q5-S157A-R | TTAGTTCTCAATTCCTTATTTTTTTC |
| MAD2-RQ-F | GAAATCCGTTCAGTGATCGAGGCGATCACAGCTACGGTGAC |
| MAD2-RQ-R | GTCACCGTAGCTGTGATCGCCTCGATCACTGAACGGATTTC |
| plenti-CAMK2D-AsiSI-F | AAAGCGATCGCATGGCTTCGACCACAACCTGC |
| plenti-CAMK2D-XhoI-R | AAACTCGAGGATGTTTTGCCACAAAGAGGTGC |
| BirA*-CAMK2D-EcoRI-F | AAAGAATTCATGGCTTCGACCACAACCTGC |
| BirA*-CAMK2D-BamHI-R | AAAGGATCCTTAGATGTTTTGCCACAAAGAGGTGCC |
| Flag-RNF8-EcoRI-F | AAAGAATTCATGGGGGAGCCCGGCTTCTTCG |
| Flag-RNF8-XhoI-R | AAACTCGAGGAACAATCTCTTTGCTTTTCG |
