# Supplementary figures and images for: CAMK2D serves as a molecular scaffold for RNF8-MAD2 complex to induce mitotic checkpoint in glioma
Source: Cell Death Differ. 2023 Jul 19;30(8):1973–87. doi: 10.1038/s41418-023-01192-3 (PMC10406836; doi:10.1038/s41418-023-01192-3)

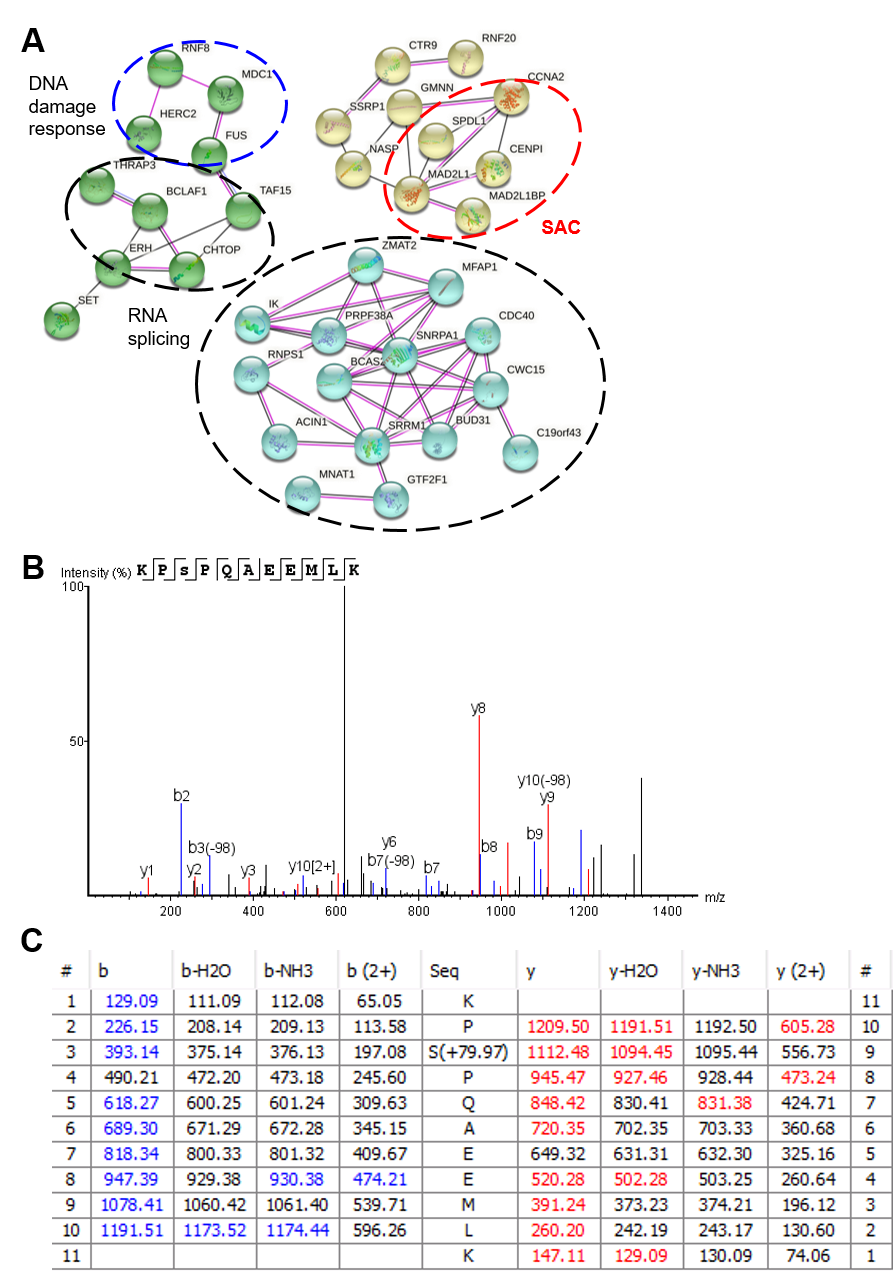

Supplement: Supplementary file 3 — Figure S1 [file 41418_2023_1192_MOESM3_ESM.tif]

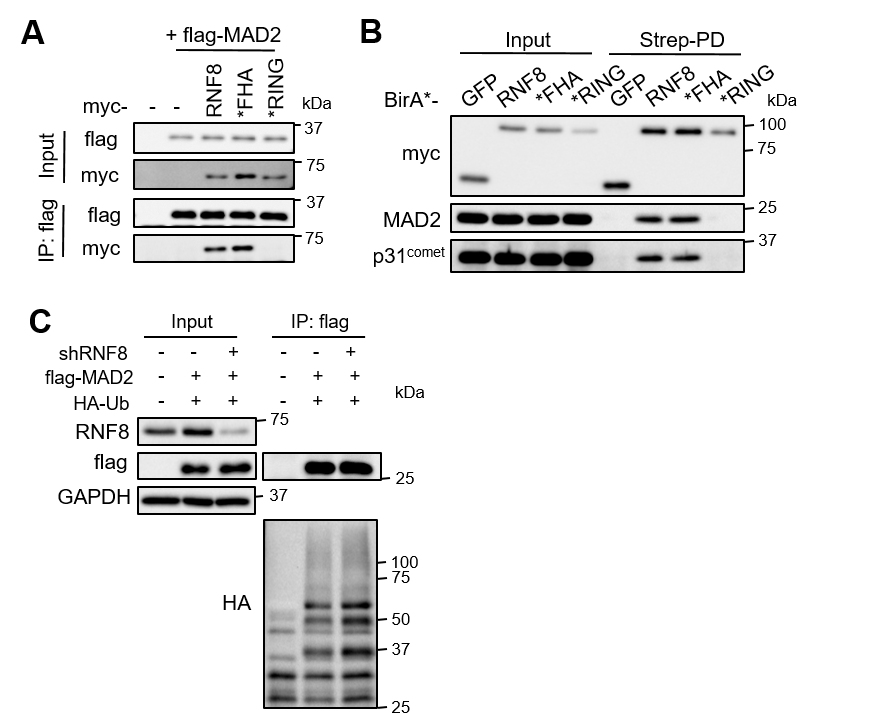

Supplement: Supplementary file 4 — Figure S2 [file 41418_2023_1192_MOESM4_ESM.tif]

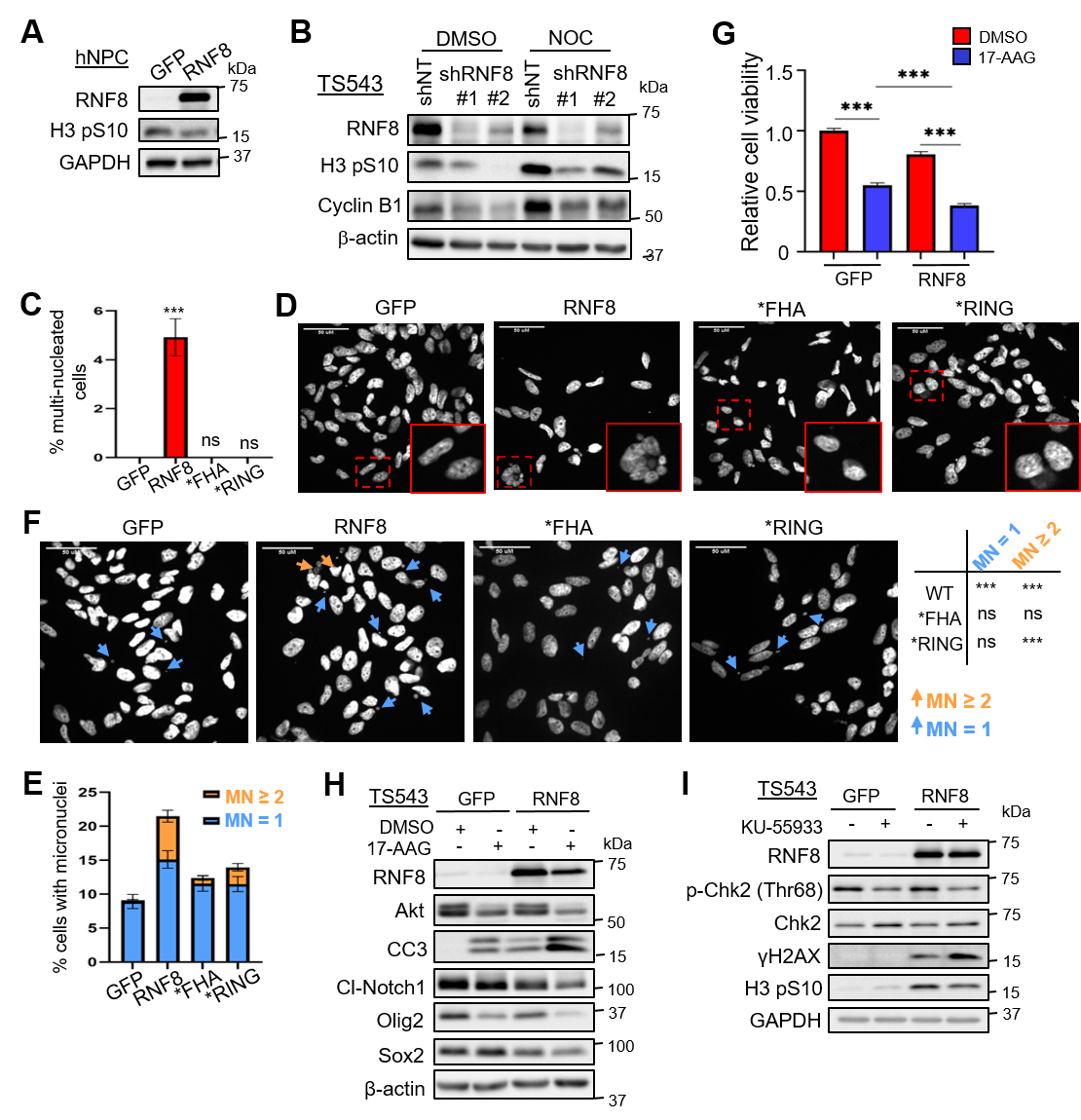

Supplement: Supplementary file 5 — Figure S3 [file 41418_2023_1192_MOESM5_ESM.tif]

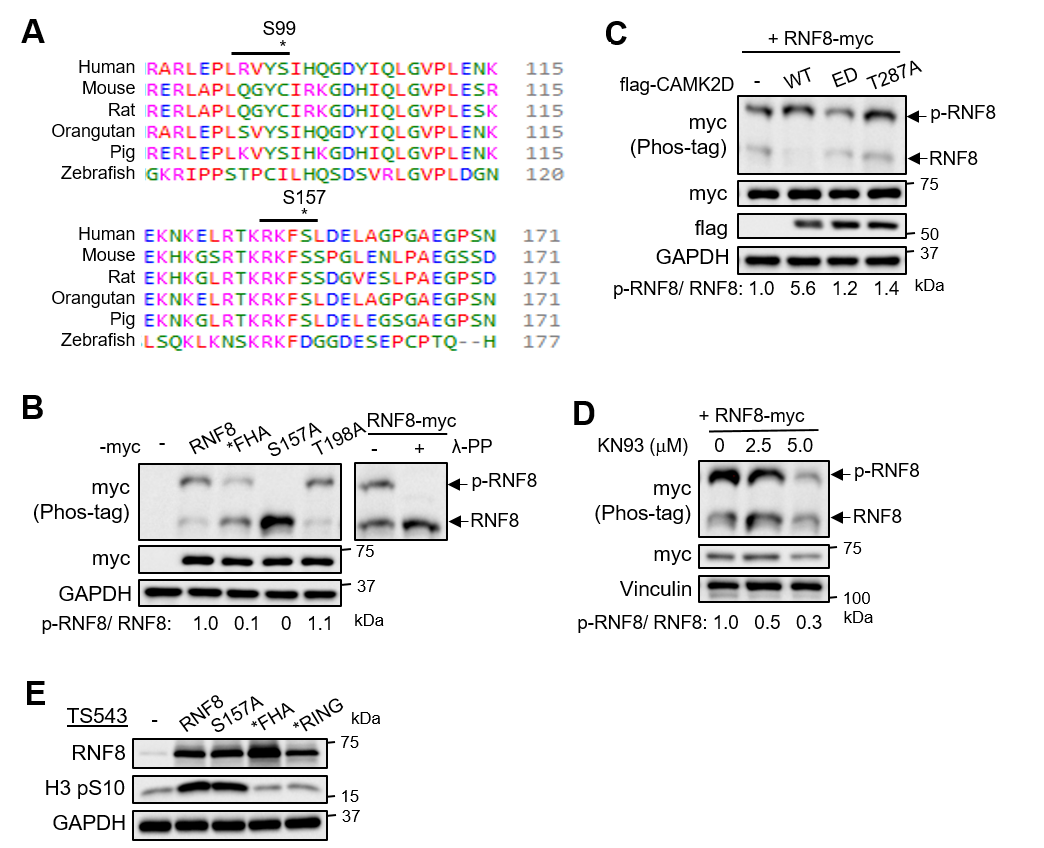

Supplement: Supplementary file 6 — Figure S4 [file 41418_2023_1192_MOESM6_ESM.tif]

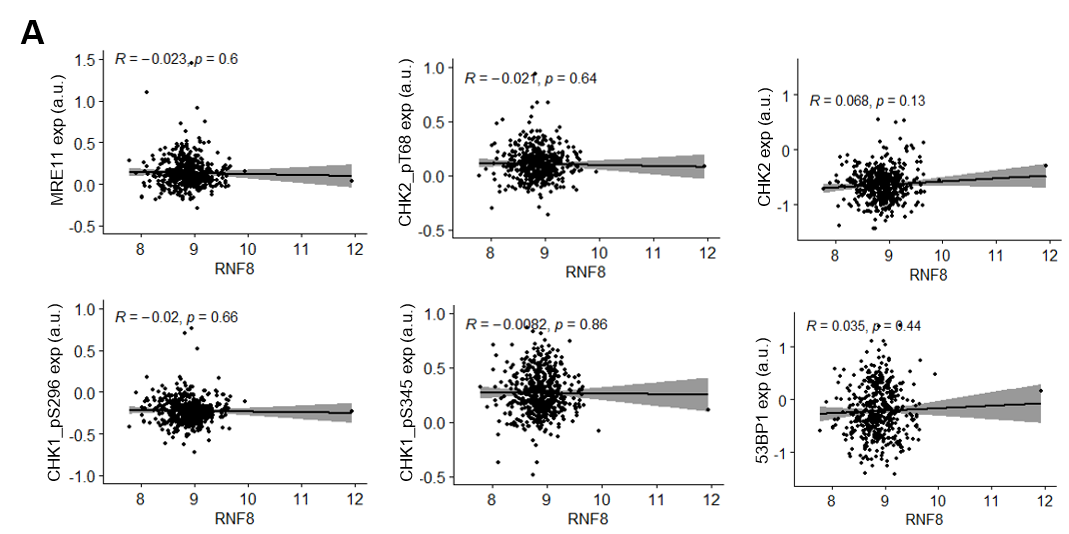

Supplement: Supplementary file 7 — Figure S5 [file 41418_2023_1192_MOESM7_ESM.tif]

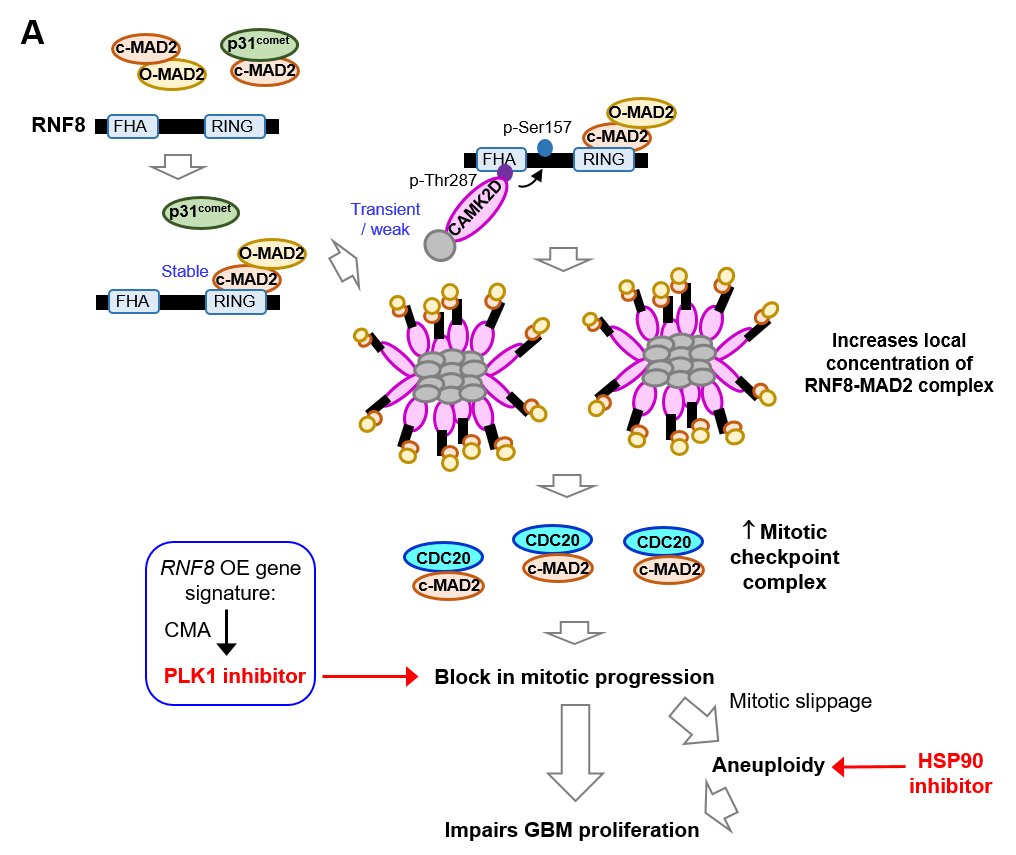

Supplement: Supplementary file 8 — Figure S6 [file 41418_2023_1192_MOESM8_ESM.tif]

**A**

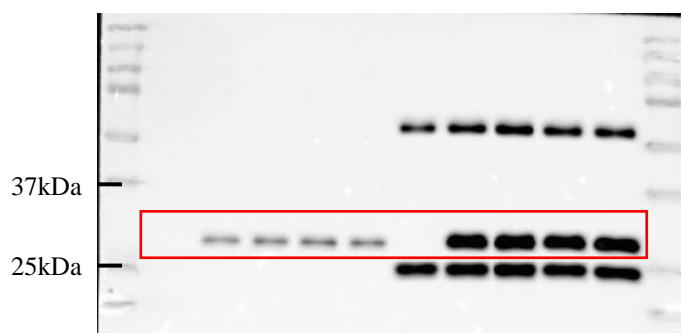

IB: flag

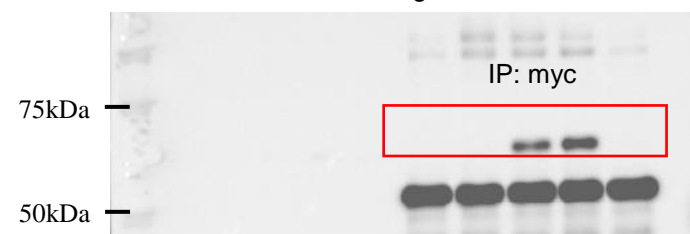

IP: myc

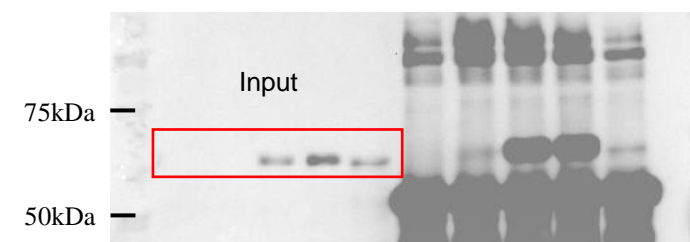

Input

IB: myc

**B**

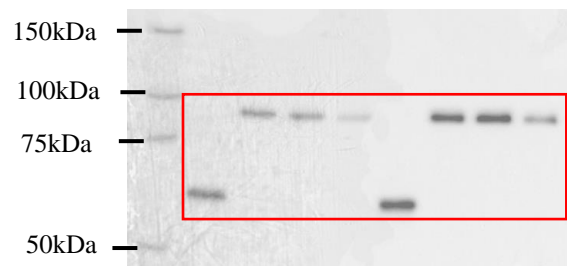

IB: myc

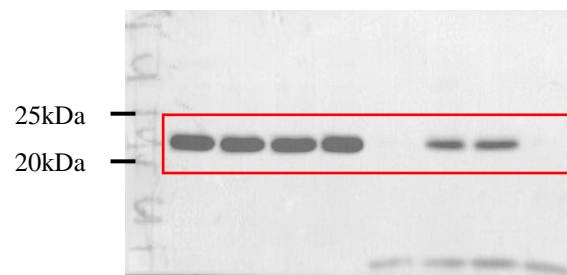

IB: MAD2

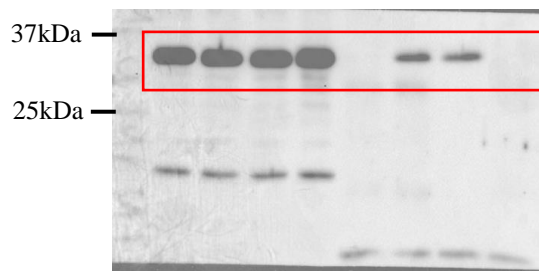

IB: p31<sup>comet</sup>

C

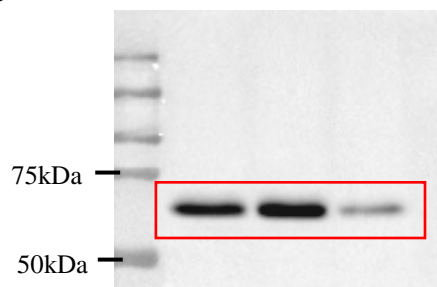

IB: RNF8

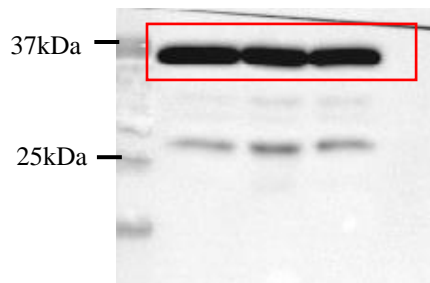

IB: GAPDH

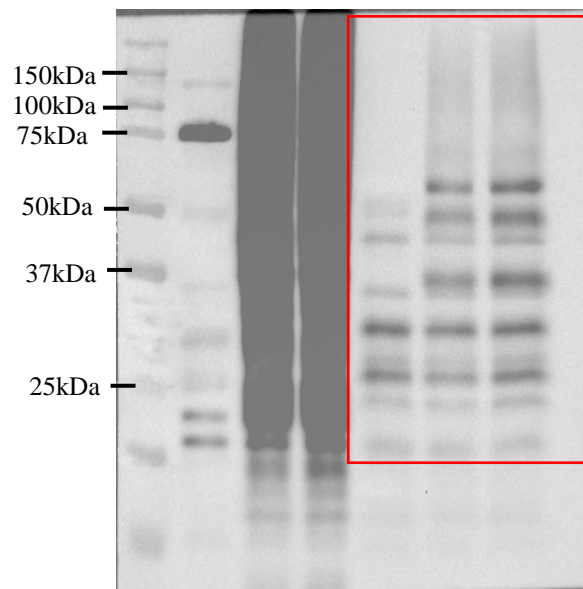

IB: HA

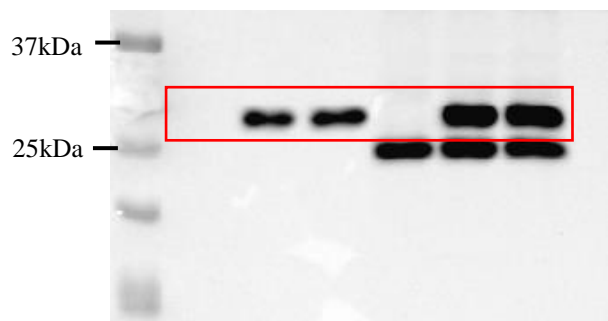

IB: flag

**A**

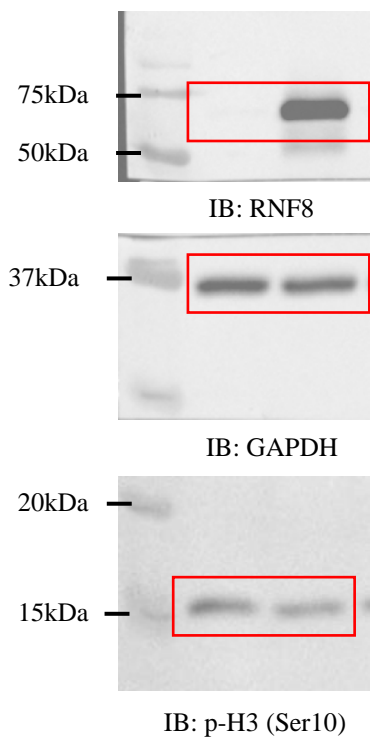

**B**

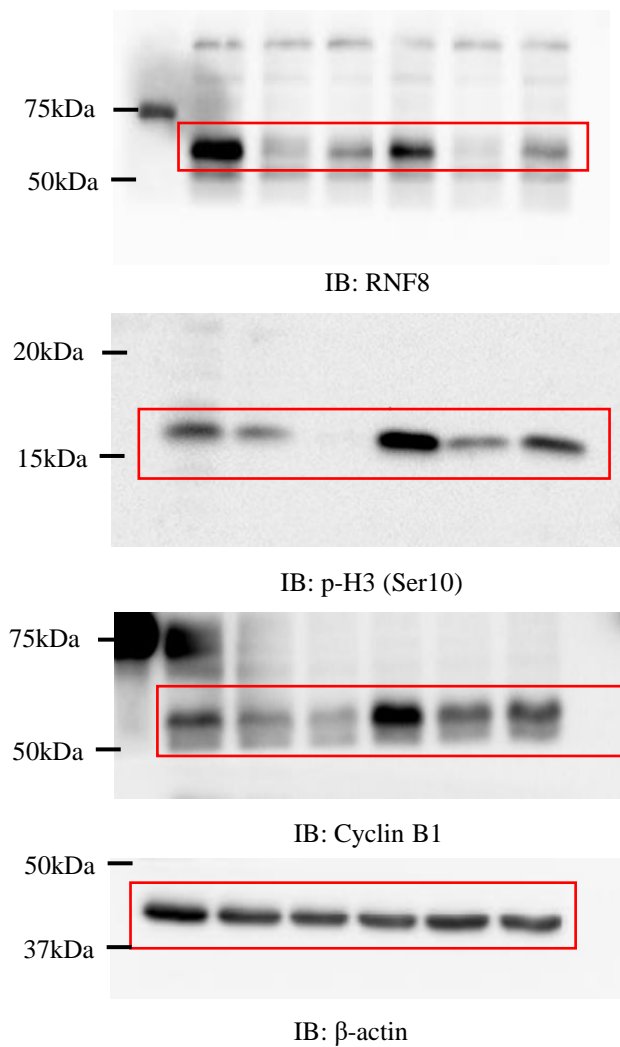

**H**

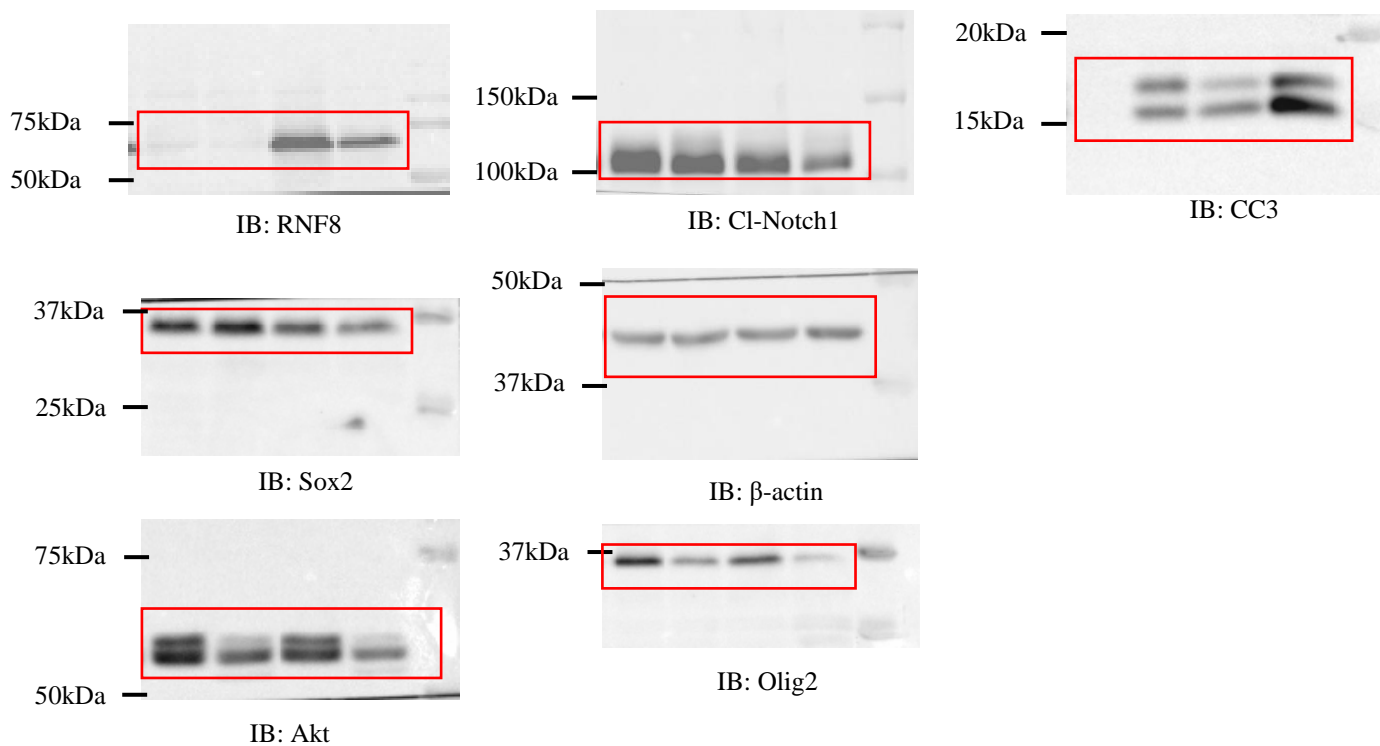

**I**

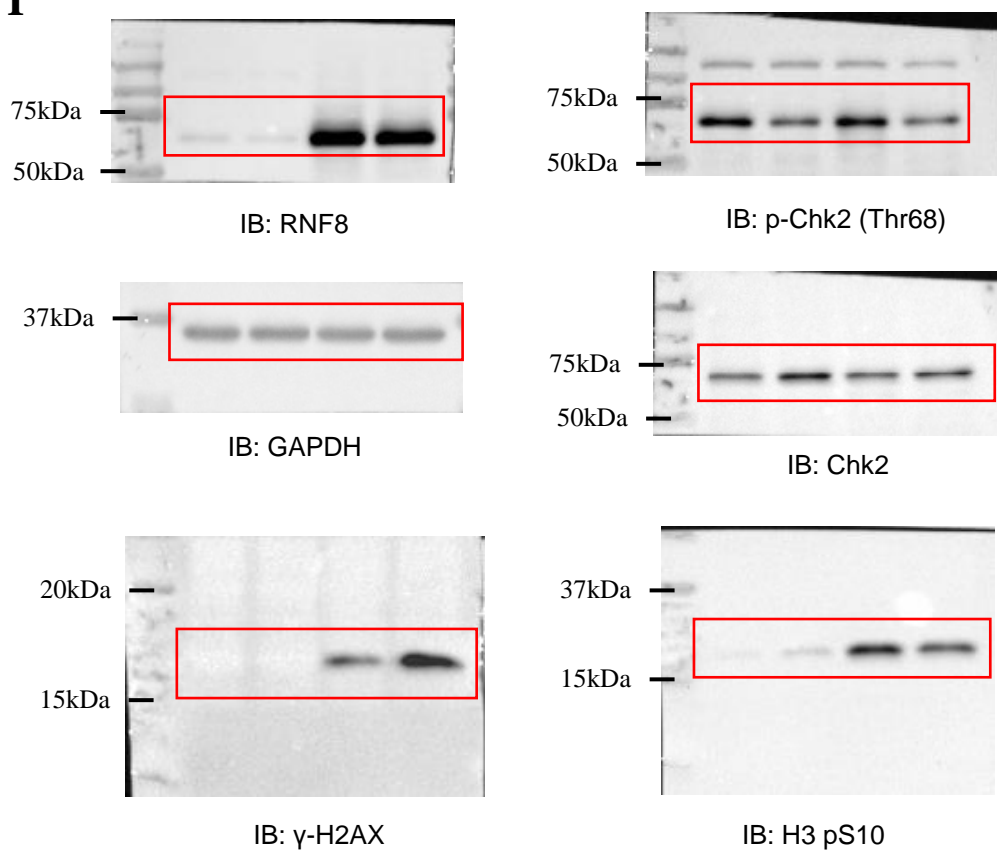

**B**

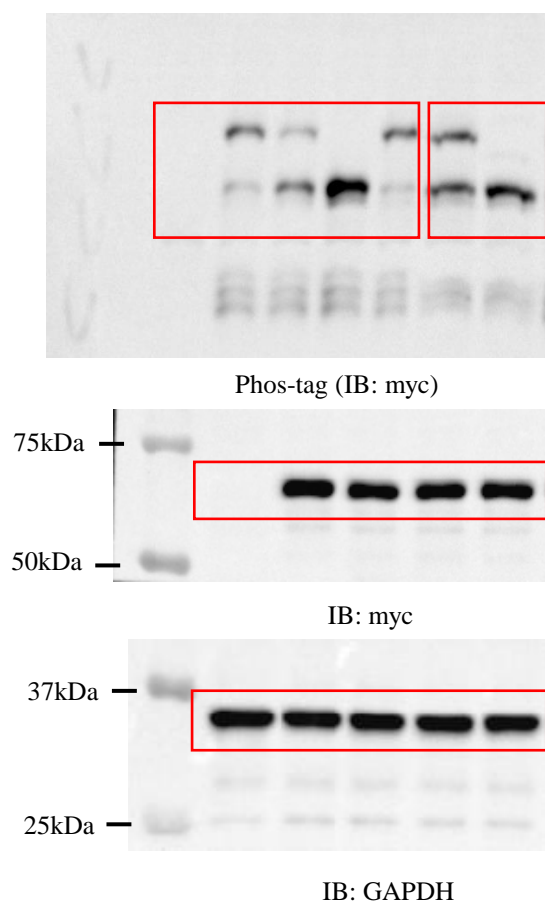

**C**

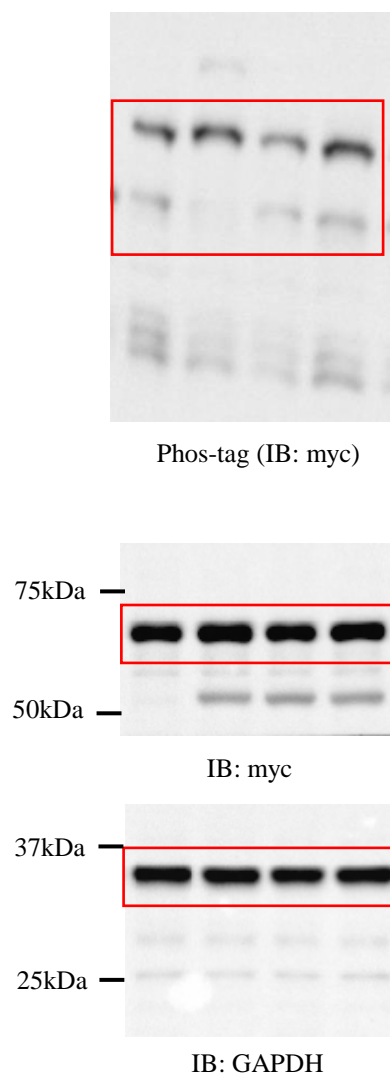

**D**

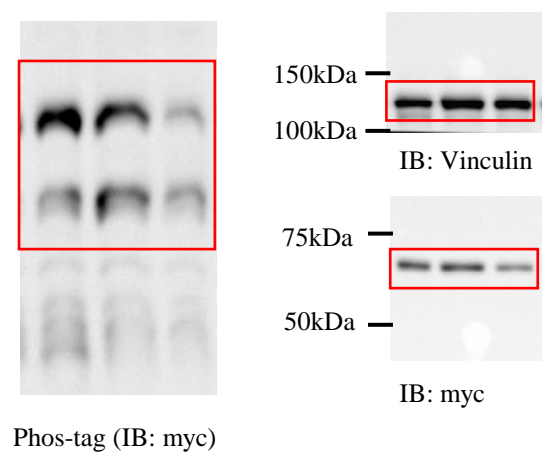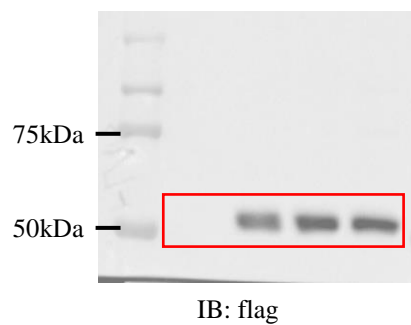

**E**

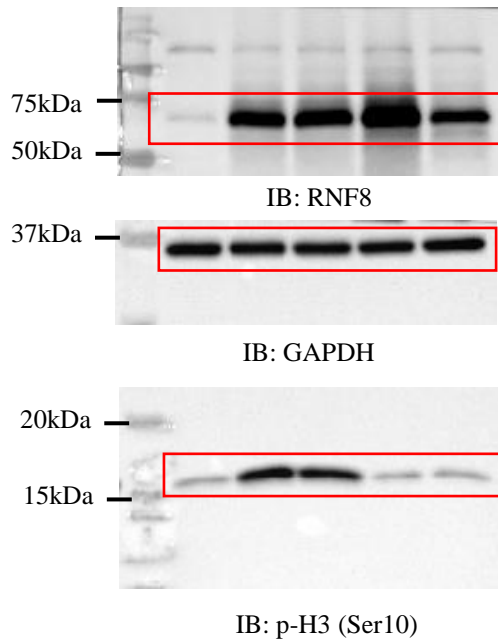

Supplement: Supplementary file 10 — Original Data File for Supp Figures [file 41418_2023_1192_MOESM10_ESM.pdf]
