# Supplementary material for: CAMK2D serves as a molecular scaffold for RNF8-MAD2 complex to induce mitotic checkpoint in glioma
Source: Cell Death Differ. 2023 Jul 19;30(8):1973–87. doi: 10.1038/s41418-023-01192-3 (PMC10406836; doi:10.1038/s41418-023-01192-3)
Supplement: Supplementary file 9 — Supplementary Materials [file 41418_2023_1192_MOESM9_ESM.docx]

**SUPPLEMENTARY INFORMATION**

**CAMK2D serves as a molecular scaffold for RNF8-MAD2 complex to induce mitotic checkpoint in glioma**

You Heng Chuah^1,2†^, Emmy Xue Yun Tay^1,2†^, Oleg V. Grinchuk^1,2†^, Jeehyun Yoon^1,2^, Jia Feng^1^, Srinivasaraghavan Kannan^3^, Matius Robert ^1,4^, Rekha Jakhar^1,4^, Yajing Liang^1^, Bernice Woon Li Lee^1,2^, Loo Chien Wang^5^, Yan Ting Lim^5^, Tianyun Zhao^5^, Radoslaw M Sobota^5^, Guang Lu^6^, Boon Chuan Low^7,8,9^, Karen Carmelina Crasta^1,2,4,10^, Chandra Shekhar Verma^3,8,11^, Zhewang Lin^8^, Derrick Sek Tong Ong^1,2,10,12^*

**Supplementary figure legends**

**Supplementary Fig. S1. RNF8 binds to MAD2 to promote MCC formation.** (**A**) STRING analysis of the significantly enriched BirA*-RNF8’s vs –GFP interactors (log_2_FC≥1, adj p value < 0.05). (**B**) LC-MS/MS analysis of flag-p31^comet^ tryptic digests showed that ^100^KPPQAEEMLK^110^ peptide contained phosphorylation at serine 102 residue. (**C**) Summary of peptide features detected by LC-MS/MS from (**B**).

**Supplementary Fig. S2. RNF8 associates with c-MAD2 stably via its RING domain without ubiquitinating MAD2.** (**A**) Western blot analysis of myc levels in flag IP from HEK293T cell lysates overexpressing myc-tagged RNF8, *FHA, *RING, or EV, along with flag-tagged MAD2. (**B**) Western blot analysis of MAD2 and p31^comet^ levels in the streptavidin pulldown lysates of HEK293T overexpressing BirA*-GFP, -RNF8, -*FHA or -*RING. (**C**) Western blot analysis of HA levels in flag IP from HEK293T cell lysates overexpressing flag-tagged MAD2 and HA-tagged Ub, with RNF8 knockdown.

**Supplementary Fig. S3. RNF8 overexpression impairs GBM mitotic progression that is dependent on its FHA and RING domains.** (**A**) Western blot analysis of H3 pS10 levels in human neural progenitor cells (hNPCs) overexpressing GFP or RNF8. RNF8 and GAPDH serve as positive and loading controls, respectively. (**B**) Western blot analysis of H3 pS10 and cyclin B1 levels in GSC TS543 with or without RNF8 knockdown, in the absence or presence of 50ng/ml NOC (16 h). RNF8 and β-actin serve as the positive and loading controls respectively. (**C, D**) Nuclear abnormality analysis of GSC TS543 overexpressing different RNF8 constructs (n=3) (mean ± SD). (**D**) Representative images of multinucleated cells from GSC TS543 overexpressing different RNF8 constructs from (**C**). Red boxes correspond to single or multinucleated cells. Scale bar: 50 µm. ****P* < 0.001. (**E, F**) Nuclear abnormality analysis of GFP, RNF8, *FHA, and *RING-overexpressed GSC TS543 (n=3) (mean ± SD). (**F**) Representative images of micronuclei from GSC TS543 overexpressing different RNF8 constructs (**E**). Coloured arrows correspond to single or multiple micronuclei bearing cells. Scale bar: 50 µm. ****P* < 0.001. (**G**) Cell viability assay of GFP or RNF8 overexpressing GSC TS543, with or without 10µM 17-AAG (24 h) treatment (n=6) (mean ± SD). ****P* < 0.001. (**H**) Western blot analysis of cleaved caspase-3 (CC3) and select GSC stemness marker levels in GFP or RNF8 overexpressing GSC TS543, with or without 10µM 17-AAG treatment (24hr). RNF8 and Akt serve as the positive controls, while β-actin serves as the loading control. (**I**) Western blot analysis of H3 pS10 and γH2AX levels in GFP or RNF8 overexpressing GSC TS543, with or without 5 µM KU-55933 treatment (24 h). RNF8 and p-CHK2 (T68) serve as the positive controls, while GAPDH serves as the loading control.

**Supplementary Fig. S4. CAMK2D phosphorylates RNF8 at S157, but this phosphorylation event is not sufficient to activate mitotic checkpoint.** (**A**) Multiple sequence alignment analysis of RNF8 protein sequences from different species using the Clustal Omega program. CAMKII phosphorylation motifs (RXXS/T) were predicted using the PhosphoMotif Finder software. (**B**) Phos-tag SDS-PAGE analysis of myc-tagged RNF8, *FHA, S157A and T198A overexpressing 293T lysates. The myc-tagged RNF8 was treated with lambda phosphatase (λ-PP) as a positive control. Relative phosphorylation of various RNF8 mutants is indicated by p-RNF8/RNF8 ratio, after normalization to RNF8. (**C, D**) Phos-tag SDS-PAGE analysis of myc-tagged RNF8, along with co-expression of CAMK2D, CAMK2D^ED^ or CAMK2D^T287A^ mutants (**C**), or 72 h of KN93 treatment (**D**). (**E**) Western blot analysis of H3 pS10 levels in GSC TS543 overexpressing RNF8, S157A, *FHA or *RING mutants. RNF8 and GAPDH serve as the positive and loading controls, respectively.

**Supplementary Fig. S5. RNF8 expression does not significantly correlate with DDR markers in glioma.** (**A**) Correlative analysis of *RNF8* mRNA levels with select DDR markers in gliomas using the TCGA RPPA dataset.

**Supplementary Fig. S6. Proposed model of a role of CAMK2D as a molecular scaffold for RNF8-MAD2 complex to generate mitotic checkpoint signal in glioma.** (**A**) RNF8 associates with MAD2 and CAMK2D via its RING and FHA domains, respectively to induce mitotic checkpoint in gliomas.
